# C5aR1 inhibition reprograms tumor associated macrophages and reverses PARP inhibitor resistance in breast cancer

**DOI:** 10.1038/s41467-024-48637-y

**Published:** 2024-05-27

**Authors:** Xi Li, Alfonso Poire, Kang Jin Jeong, Dong Zhang, Tugba Yildiran Ozmen, Gang Chen, Chaoyang Sun, Gordon B. Mills

**Affiliations:** 1 Division of Oncological Sciences Knight Cancer Institute, Oregon Health and Science University, Portland, OR USA; 2 Department of Obstetrics and Gynecology, Tongji Hospital, Tongji Medical College, Huazhong University of Science and Technology, Wuhan, China

**Keywords:** Cancer genomics, Cancer microenvironment, Immunoediting, Cancer microenvironment

## Abstract

Although Poly (ADP-ribose) polymerase (PARP) inhibitors (PARPi) have been approved in multiple diseases, including BRCA1/2 mutant breast cancer, responses are usually transient requiring the deployment of combination therapies for optimal efficacy. Here we thus explore mechanisms underlying sensitivity and resistance to PARPi using two intrinsically PARPi sensitive (T22) and resistant (T127) syngeneic murine breast cancer models in female mice. We demonstrate that tumor associated macrophages (TAM) potentially contribute to the differential sensitivity to PARPi. By single-cell RNA-sequencing, we identify a TAM_C3 cluster, expressing genes implicated in anti-inflammatory activity, that is enriched in PARPi resistant T127 tumors and markedly decreased by PARPi in T22 tumors. Rps19/C5aR1 signaling is selectively elevated in TAM_C3. C5aR1 inhibition or transferring C5aR1^hi^ cells increases and decreases PARPi sensitivity, respectively. High C5aR1 levels in human breast cancers are associated with poor responses to immune checkpoint blockade. Thus, targeting C5aR1 may selectively deplete pro-tumoral macrophages and engender sensitivity to PARPi and potentially other therapies.

## Abstract (web summary)

PARP inhibitors (PARPi) have been approved for the treatment of metastatic triple-negative breast cancer (BC), however resistance and recurrence are often observed. Here, in preclinical models of BRCA1/2 wild type and homologous recombination competent BC, the authors show that C5aR1-positive tumor associated macrophages are associated with PARPi-resistance, suggesting targeting C5aR1 as a therapeutic option.

## Introduction

Poly (ADP-ribose) polymerase (PARP) inhibitors (PARPi) have been extensively explored in the laboratory and the clinic based on synthetic lethality with defects in homologous recombination (HR) mediated by mutations in BRCA1/2 and other aberrations in the HR pathway. Although PARPi in combination with chemotherapy have been approved for management of BRCA-associated metastatic triple-negative breast cancer (TNBC)^1,2^, benefits are not durable with almost universal recurrence. The mechanisms underlying PARPi resistance have been explored extensively and include BRCA1/2 reversion mutations, hypomorphic BRCA1/2 alleles, loss of hypermethylation of BRCA1/2, loss of the shieldin complex and activation of pro-survival pathways such as the MAPK and PI3K pathways^3–7^. However, taken together, the identified mechanisms can only explain PARPi resistance in a subset of patients^8–10^. In terms of extending the utility of PARPi to a wider population of TNBC patients, multiple clinical studies have demonstrated that PARPi combinations are active in patients whose do not have BRCA1/2 mutations or other aberrations in the HR pathway providing an opportunity to provide benefit patients whose tumors do not have aberrations in BRCA1/2^9,11–19^. Thus, there is a high priority for understanding resistance mechanisms to PARPi in tumors with and without BRCA1/2 mutations or aberrations in the HR pathway and identifying and implementing novel combinations therapies that are effective across mutational subtypes and that prevent the emergence of resistance or abrogate PARPi resistance once it occurs^20,21^.

Macrophages, innate immune cells derived from the myeloid lineage, exhibit remarkable epigenomic and transcriptomic plasticity driven, in part, by changes in the microenvironment^22^. Tumor associated macrophages (TAMs) can contribute to tumor clearance or protection based on their polarization into macrophages that mediate anti-tumor/pro-inflammatory or pro-tumor effects, respectively^23^. Pro-inflammatory and pro-tumor TAMs mediate many of their pro and anti-tumor activities through production of mutually exclusive sets of cytokines. Pro-inflammatory TAMs express T_H_1 response-inducing cytokines such as IL1α, IL1β, IL12 and TNFα while pro-tumor TAMs express IL10, CCL8, and CCL22 that are tumor promoting^24^. However, single-cell RNA-sequencing (scRNA-seq) studies have demonstrated that the dichotomous anti-tumor/pro-inflammatory or pro-tumor macrophage polarization classification do not adequately capture the diversity and plasticity of macrophage states^25,26^. Different TAMs subtypes are frequently associated with a mixed expression of genes with angiogenesis, phagocytosis, pro-inflammatory, proliferation, metastasis, checkpoint inhibition and metabolism activities rather than solely one functional gene expression group^25,26^.

We and others have demonstrated that PARPi can induce a functional STING response with resultant production of interferons (IFNs) and immune engagement with subsequent sensitization to immune checkpoint blockade (ICB) in both BRCA1/2 mutant and wild type cells^3,27,28^. Furthermore, STING-induced immune activation appears to contribute to the activity of PARPi combination therapy with ICB in clinical trials including in patients without demonstrable abnormalities in BRCA1/2^15,29,30^. Interestingly, STING agonists can promote PARPi sensitivity by reprogramming TAMs to a dominant pro-inflammatory phenotype in BRCA1-deficient breast cancer models^31,32^. However, despite robust activity in preclinical models and evidence for engagement of STING responses and the immune system, combinations of PARPi and ICB have generally been disappointing in clinical trials, potentially due to a lack of understanding of the underlying mechanisms and a paucity of predictive biomarkers^33^.

Here, we use two mouse-derived syngeneic transplant (MDST) models developed from transgenic mice expressing the lysophosphatidic acid1 (LPA1) receptor in the mammary epithelium that are intrinsically sensitive (LPA1-T22 (T22)) or resistant (LPA1-T127 (T127)) to PARPi^34^ to explore mechanisms underlying intrinsic PARPi sensitivity and resistance. The two models are BRCA1/2 wild type and HR competent suggesting that results from these studies could be applicable beyond BRCA1/2 mutant HR deficient (HRD) tumors (Supplementary Fig. 1A)^27,33^. Single cell RNA analysis identifies 4 tumor associated macrophage (TAM) subtypes: TAM_C0, TAM_C1, TAM_C3 and TAM_C4 in the T22 and T127 models. TAM_C0 that has a mixed expression of genes associated pro- and anti-inflammatory states^35^ is present at elevated levels in the T22 intrinsically PARPi tumors. TAM_C3 that mainly expresses genes associated with an anti-inflammatory/protumor state^35^ is elevated in T22. TAM_C3 is also lower in the intrinsically PARPi sensitive T22 than in the PARPi resistant T127 and is markedly decreased by PARPi in T22. TAM_C3 that are associated with PARPi resistance could be further subdivided into C5aR1 high and C5aR1 low subpopulations. The C5aR1 high subset of TAM_C3 expresses genes associated with anti-inflammatory activity, whereas the C5aR1 low does not express significant levels of genes implicated in anti- or pro- inflammatory activity. To mimic metastatic states, we implant T22 and T127 in opposite mammary fat pads. This results in the intrinsically PARPi sensitive T22 becoming PARPi resistant. C5aR1 positive macrophages are sufficient to render T22 resistant to PARPi. Strikingly targeting C5aR1 decreases CD206^high^ macrophages in the PARPi-resistant models while sparing MHCII^high^ macrophages leading to both models becoming sensitive to PARPi.

## Results

### Co-transplantation of PARPi-resistant LPA1-T127 with PARPi-sensitive LPA1-T22 renders LPA1-T22 resistant to PARPi

**Fig. 1:**
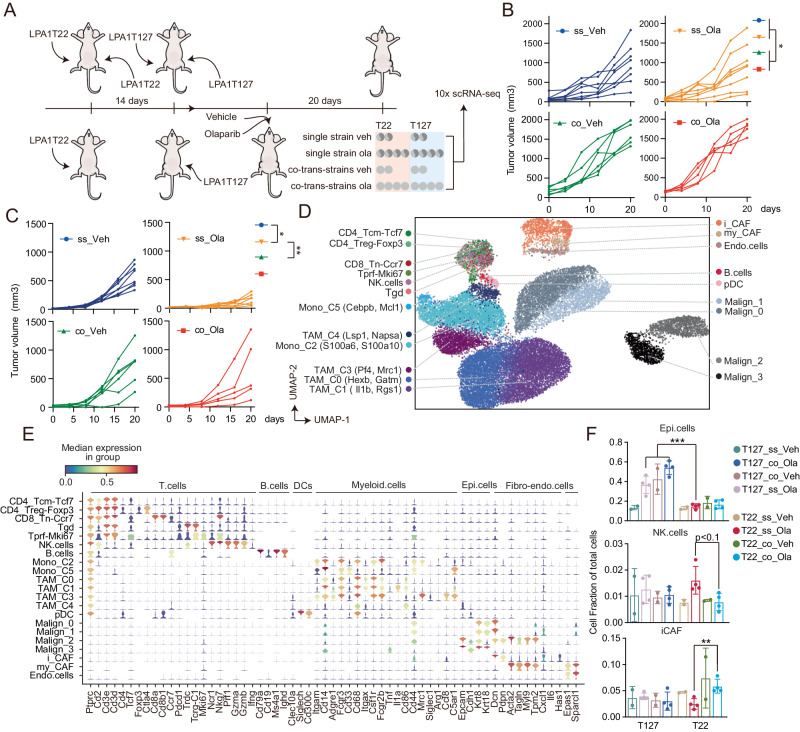
Growth and immune component analysis in T22, T127 and co-transplantation models. **A** Design of the study in T127 and T22 single strain (upper) and co-transplanted T22 and T127 (lower) MDST models receiving olaparib for 20 days. Bilateral tumors from a single mouse are represented by dark gray and light gray respectively). **B** Growth curve of T127 models with olaparib for 20 days. Tumor size of single strain transplantation models represent the average of both sides in a single strain mouse. ss: single strain transplantation models. co: T127 side of co-transplantation models. For single strain models, *n* = 8, co-transplantation treated with vehicle, *n* = 6 and co-transplantation treated with olaparib, *n* = 5. **C** Growth curve of T22 models with olaparib for 20 days. Tumor size of single strain transplantation models represent the average of both sides in a single strain mouse. ss: single strain transplantation models. co: T22 side of co-transplantation model. For single strain treated with vehicle, *n* = 8, single strain treated with olaparib, *n* = 7; co-transplantation treated with vehicle, *n* = 6 and co-transplantation treated with olaparib, *n* = 5. **D** Stratification and cell-type identification of T22 and T127 tumors from single strain and co-transplantation tumors. Malignant cells from T127 (Malign_0, Malign_1) and T22 (Malign_2, Malign_3) show distinct cell clusters. Tcm: central memory T cells, Treg: regulator T cells, Tn: naive T cells, Tgd: γδT cells, Tprf: proliferative T cells, my_CAF: myofibroblastic cancer associated fibroblast cells, i_CAF: inflammatory cancer associated fibroblast cells, pDC: plasmacytoid dendritic cells. For vehicle treated, *n* = 2 and olaparib treated, *n* = 4. **E** Violin plot of markers representing each cell lineage in Fig. 1D. **F** Bar plot showing relative numbers of epithelial (high CNV cells), NK cells and i_CAF (in %) defined by cell lineage markers listed in **E** of each model. For vehicle treated, *n* = 2 and olaparib treated, *n* = 4. Statistical significance was assessed only among olaparib treated groups. All data are presented as mean values ± SD. *p* values are from one-way ANOVA. * *p* < 0.05, ** *P* < 0.01, *** *p* < 0.001. Source data and exact *p* values are provided as a Source Data file. Source data are provided as a Source Data file. Veh: vehicle. Ola: olaparib.

LPA1-T22 (T22) and LPA1-T127 (T127), murine-derived syngeneic transplant (MDST) tumors, are intrinsically sensitive and resistant to PARPi, respectively^27^. T22 has a modest response to anti-PDL1 and a marked response to the combination of a PARPi (olaparib) and anti-PDL1 in immunocompetent mice^27^. In contrast, T127 is PARPi and anti-PDL1 resistant and furthermore does not respond to the combination^27^. As PARPi resistance is a major emerging challenge in breast and other cancers, we explored mechanisms mediating intrinsic PARPi resistance in T127 and sensitivity in T22. Furthermore, to mimic the potential effects of tumor subclones in different distant sites (or within different locations in a tumor) on PARPi resistance, we explored whether PARPi resistance could be transferred systemically from T127 to T22 by transplanting T22 and T127 tumors bilaterally in the left and right mammary fat pads of the same mouse (co-transplantation or co) (Fig. 1A). As T22 becomes established more slowly than T127, T22 was transplanted two weeks before T127. As a control, T22 and T127 were transplanted bilaterally (single strain or ss) at the same time as they were introduced in the co-transplantation model (co-transplantation or co) (Fig. 1A). T22_ss transplanted bilaterally into the same mouse (T22 single strain model) remained sensitive to olaparib and T127_ss (T127 single strain model) remained resistant to olaparib (Fig. 1B and S1B). In contrast, when T22 and T127 were co-transplanted into the same mouse, T22 was rendered resistant to olaparib (Fig. 1C and S1B). For as yet unknown reasons, T127 appeared to grow faster when co-transplanted with T22 independent of whether they were treated with olaparib or vehicle (Fig. 1B and S1B).

We subsequently performed single cell RNA sequencing (scRNA-seq) analysis of the models resulting in more than 30,000 cells available across the samples (See Supplementary Fig. 1D for a list of individual sample assessed). Malignant cells were identified by CNV calling using CaSpER^36^ (Supplementary Fig. 1C). Cell lineage assignment was done according to the markers listed in Fig. 1E^37–40^. Two distinct T22 malignant cell clusters (Malignant 2 and 3) and two distinct T127 malignant cell clusters (Malign_0 and Malign_1) were captured in both single strain models and co-transplantation models (Fig. 1D, E and S1D, E, see Fig. 1E for markers used to defined cell subtypes)^35^. Malign_2 and Malign_3 from T22 had higher levels of Epcam and Cldn1 than Malig_0 and Malign_1 from T127. Malign_2 expressed a number of markers associated with the fibroblast lineage suggesting that this tumor subtype has undergone epithelial mesenchymal transition (EMT), whereas, Malign_3 expressed elevated levels of Krt8 and 18 consistent with epithelial differentiation. The malignant cell cluster distribution in T22 and T127 was not significantly altered by olaparib therapy (Supplementary Fig. 1D, E). As expected from the growth curves, the fraction of epithelial tumor cells in the T127 model was increased by olaparib and also by co-transplantation with T22 (Fig. 1F). The fraction of malignant epithelial cells in the T22 model was not significantly altered by treatment (note that the normalization to 1 partially removes the effects of therapy on tumor size) (Fig. 1F). As indicated in Supplementary Fig. 1D there were few if any malignant cells from T127 (Malign_0 and Malign_1) found in T22 (Malign_2 and Malign_3) and vice versa in the co-transplantation model eliminating the possibility that PARPi resistance in co-transplanted T22 was due to overgrowth of metastatic T127 in the T22 tumor. Importantly, in contrast to the malignant cells that were model specific, the hematopoietic, fibroblasts and endothelial cells from both T22 and T127 tumors with and without olaparib treatment co-clustered (Fig. 1D, Supplementary Fig. 1D, E). There were no statistically significant changes in T cell or B cell subtypes between single strain and co-transplantation models and treated and untreated samples (Fig. 1D and S1E) (see Fig. 1E for the markers used to define each cluster). Olaparib induced an increase in NK cells in the T22_ss model and not in T22_co (T22 side of co-transplantation model) (Fig. 1F). In contrast, olaparib induced a mild but significant increase in cancer associated fibroblast cells (CAF) in the T22_co model but not in T22_ss model. The consequence of the change in NK cells and CAF remains to be elucidated.

### Co-transplantation of T127 with T22 alters myeloid subtypes in T22 after PARPi treatment

The most striking difference in immune contexture between the models was in fractions of different TAM subclusters based on re-clustering of cells identified as monocyte and macrophage populations in the original clusters. Unsupervised re-clustering of the monocyte and macrophage populations (Supplementary Fig. 1F) identified two monocyte populations (Mono_C2 and Mono_C5 with higher Cd14 expression) and 4 macrophage populations (TAM_C0, TAM_C1, TAM_C3 and TAM_C4 with higher Cd68 expression) characterized by expression of a number of markers associated with monocyte and macrophage function (Supplementary Fig. 1F, G see Supplementary Data 1 for a list of genes expressed in each population)^41^. The monocyte and TAM clusters were annotated as unsupervised Leiden clusters, projected onto Fig. 1D and then designated based on the top DEGs for each of the Leiden clusters. The monocyte and TAM populations were subsequently evaluated for expression of characteristic monocyte and macrophage markers defined in previous publications^38–40^. TAM_C0, characterized by Hexb and Gatm DEGs expressed a mixture of genes associated with pro-inflammatory, anti-inflammatory, phagocytosis and checkpoint activity (Supplementary Fig. 1H, I). TAM_C1, characterized by Il1b and Rgs1 DEGs, clustered close to TAM_C0 and was enriched for a series of markers associated with pro-inflammatory activity^38^ as well as KEGG necroptosis and GO interferon gamma related pathway activity (Supplementary Fig. 1H, I and Supplementary Fig. 2A). TAM_C3, characterized by Mrc1 and Pf4 DEGs, was enriched for expression genes associated with pro-tumor activity including anti-inflammatory, phagocytosis and checkpoint activities^35^ as well as KEGG lipid related pathway and GO type I interferon-related pathway activity (Supplementary Fig. 1H, I and Supplementary Fig. 2A). TAM_C4, characterized by Lsp and Napsa DEGs, like TAM_C1, was most clearly associated with pro-inflammatory markers and its relative low level of Adgre1 suggests a potential relationship to macrophage progenitors^42^(Supplementary Fig. 1H, I). Mono_C2, characterized by S100a6 and S100a10 DEGs, and Mono_C5, characterized by Cebpb and Mcl1 DEGs, both expressed genes associated with angiogenesis with Mono_C5 also expressed genes associated with checkpoint activity (Supplementary Fig. 1H, I).

Label transfer was then used to further characterize the TAM subtypes (See methods) in comparison to previously identified subtypes from a large scale analysis of pan-cancer infiltrating myeloid cells^26^. TAM_C1 had a score predicted to associate Macro_IL1B and Macro_ISG15 subtypes, which were reported to relate with pro-inflammatory activities and also had a relatively higher prediction score of Macro_GPNMB, which was previously reported as an anti-inflammatory and metabolism related subtype^43,44^ (Supplementary Fig. 1J). TAM_C3 was highest in Macro_LYVE1 and Macro_C1QC macrophage subtypes that have been reported to be associated with an anti-inflammatory subtype (Supplementary Fig. 1J). TAM_C0 was similar to TAM_C1 and was also associated with a Macro_GPNMB phenotype but had lower Macro_IL1B and Macro_ISG15 signatures than TAM_C1. TAM_C4 had scores related cDC cell scores and would require further exploration. For further clarification of macrophage phenotypes, a reverse annotation was done by scGPT with MDST datasets as a training model and a large human myeloid dataset as a test data^45^ (See Methods). Similarly to the label transfer results, the majority of the human pro-inflammatory Macro_IL1B macrophages were annotated as TAM_C1 while more than half of the anti-inflammatory Macro_LYVE1 and Macro_C1QC macrophages were annotated as TAM_C3 (Supplementary Fig. 1K). As indicated in other studies with different TAM subsets could express a mixture of genes associated with pro-and anti-inflammatory subtypes^44,46,47^ and also found in our study, TAM_C0, TAM_C1 and TAM_C4 were comprised a mixture of pro- and anti-inflammatory phenotypes. However, the strong association of TAM_C1 with expression of genes associated with pro-inflammatory activity and TAM_C3 with expression of genes associated with pro-tumorigenic/anti-inflammatory activity suggested that they were likely contributors to the relative responsiveness of T22 and T127 to olaparib.

As different TAM subtypes had been associated with outcomes in cancer patients, we explored the contributions of the TAM subtypes identified in our study with outcomes in the TCGA basal breast cancer cohort. For this purpose we calculated cell signature rank of each cell lineage with UCell followed by multivariate survival analysis (see methods, Supplementary Fig. 2B). Consistently with the gene expression patterns described above, a higher TAM_C3 signature was significantly correlated with a poor outcome. A higher NK cell score was significantly correlated with a favorable outcome. None of the other signature scores were associated with outcomes in TCGA basal breast cancers.

**Fig. 2:**
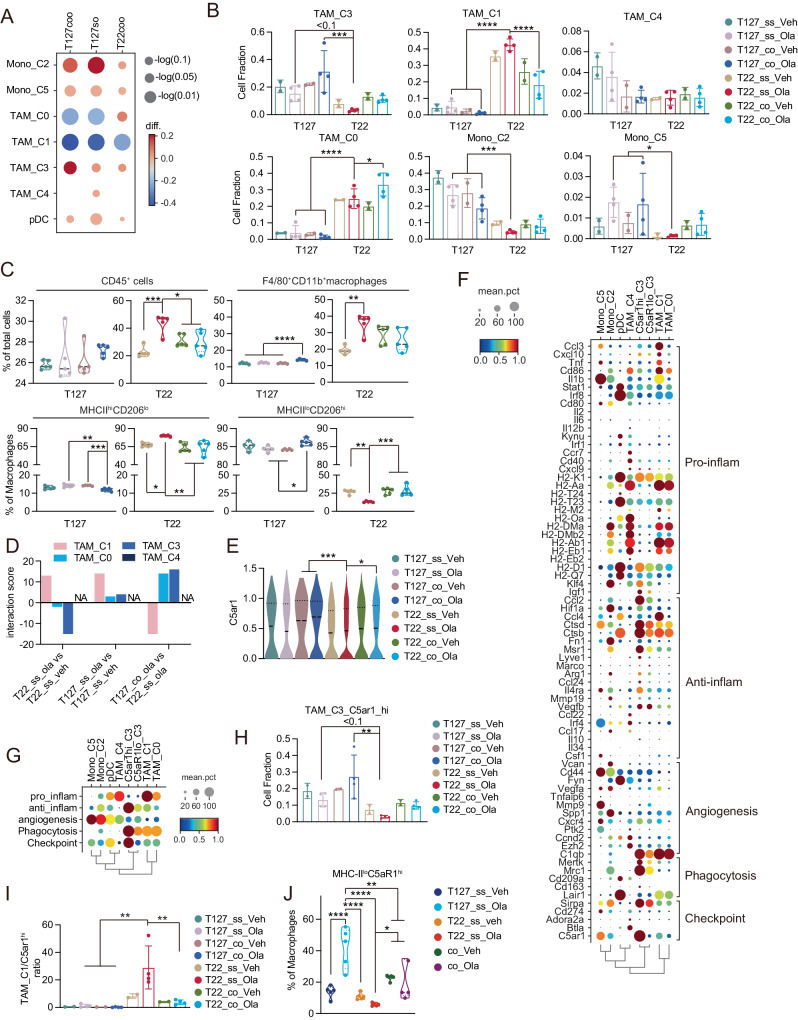
Macrophage subtyping in T22, T127 and co-transplantation models. **A** Dot plot showing relative differences of indicated cell types of each indicated model compared with that cell types in T22 single transplanted model in scRNAseq datasets from cells in Fig. 1. *n* = 4. **B** Cell fractions of indicated cell types in each model in scRNAseq datasets from cells in Fig. 1. Statistical significance was assessed only among olaparib treated groups. **C** Cell fractions of CD45^+^ cells, total macrophages (CD45^+^F4/80^+^CD11b^+^), pro-inflammatory (CD45^+^F4/80^+^CD11b^+^MHCII^hi^CD206^lo^), pro-tumor (CD45^+^F4/80^+^CD11b^+^MHCII^lo^CD206^hi^) TAMs in each model assessed by flow cytometry. *n* = 5. **D** Bar plot showing decreased ligand-receptor pair interaction count estimate between malignant cells and indicated TAM cells from indicated comparison pairs. **E** Violin plot shows relative expression level of C5ar1 in TAM cells from indicated groups. **F** Dot plot of markers representing each pathway of indicated TAM clusters. **G** Dot plot of summarized expression level of each pathway of indicated TAM clusters. **H** Bar chart shows ratio of TAM_C3_C5ar1_hi cells in each model from the scRNAseq datasets of Fig. 1. Statistical significance was assessed among only in olaparib treated groups. **I** Bar chart shows ratio of TAM_C1 cells and TAM_C3_C5ar1_hi cells in each model from the scRNAseq datasets of Fig. 1. Statistical significance was assessed only in olaparib treated groups. **J** Fraction of MHC-II^lo^C5aR1^hi^ cells of total macrophages (see Supplementary Fig. 2 for gating strategy) from peripheral blood from each indicated treatment group assessed by flow cytometry. *n* = 5. Statistical significance was assessed only in olaparib treated groups. For **B**–**E** and **H**–**I**, all data are presented as mean values±SD, vehicle treated, *n* = 2 and olaparib treated, *n* = 4. For all data, *p* values are from one-way ANOVA. * *p* < 0.05, ** *P* < 0.01, *** *p* < 0.001, **** *p* < 0.0001. Source data and exact *p* values are provided as a Source Data file. Source data are provided as a Source Data file.

As compared to the olaparib-treated PARPi sensitive T22 single strain model, the olaparib-treated PARPi resistant T127 (both models) and T22 co-transplantation model showed marked differences in TAM and monocyte populations (Fig. 2A, B). Whereas TAM_C3 was markedly decreased by PARPi in the T22 single strain model, this decrease was abrogated in the T22 co-transplantation model indicating that the PARPi resistance in the co-transplantation model was associated with a change in effect of PARPi on TAM_C3 that was dominantly associated with expression of genes with anti-inflammatory activity (Supplementary Fig. 1H, I) and associated with a worse outcome in TCGA basal breast cancer (Supplementary Fig. 2A, B). In contrast, olaparib did not decrease TAM_C3 in either T127 model. As compared to the olaparib-treated T22 single strain model, the olaparib-treated T22 co-transplantation model showed a decrease in TAM_C1 and an increase in the TAM_C0, population (Fig. 2A, B). Interestingly, TAM_C1 which was increased in the T22 single strain model was decreased in T22 tumors in the co-transplantation model and further decreased by olaparib treatment. TAM_C0 was higher in both T22 single strain and T22 co-transplantation models than in the T127 models with the elevated levels maintained or slightly increased in the presence of olaparib. Mono_C2 was higher in both T127 single strain and T127 co-transplantation models than in the T22 models (Fig. 2B). Interestingly, olaparib slightly decreased Mono_C2 in all models. Mono_C5 was increased by olaparib in both T127 models but in contrast was either unchanged or slightly decreased in both T22 models (Fig. 2B). Taken together, based on the gene expression patterns associated with TAM_C1 and TAM_C3 suggest that a decrease in TAM_C1 and an increase in TAM_C3 in the T22 co-transplantation model represent the most likely TAM populations to contribute to the effect of co-transplantation of T127 on the decreased sensitivity of T22 to PARPi.

Mrc1, encoding CD206, was highly expressed in TAM_C3 and was very low in TAM_C0, TAM_C1, and TAM_C4 (Supplementary Fig. 2C). In contrast, MHCII, which is required for anti-tumor T cell response^48^, was also highly expressed by TAM_C0, TAM_C1 and TAM_C4, but is very low in TAM_C3 (Supplementary Fig. 2C). In flow cytometry, (F4/80^+^MHCII^lo^/CD206^hi^) macrophages would represent TAM_C3 macrophages and (F4/80^+^MHCII^hi^/CD206^lo^) would represent TAM_C0 and TAM_C1 macrophages. Flow cytometry showed that total CD45^+^ hematopoietic cells as well as CD45, F4/80 and CD11b positive macrophages were increased in T22 single strain and T22 co-transplantation models compared to T127 (Fig. 2C and Supplementary Fig. 2D–F). Olaparib induced an increase in CD45+ hematopoietic cells as well as F4/80^+^CD11b^+^ macrophages in the T22 single strain models that was abrogated in the T22 co-transplantation model. Strikingly, T22 had markedly elevated levels of CD45^+^, F4/80^+^CD11b^+^, MHCII^high^/CD206^low^ macrophages that represented TAM_C0, TAM_C1, and TAM_C4 macrophages that were increased by olaparib in the T22 single strain model with the olaparib induced increase being abrogated in the T22 co-transplantation model. The converse was true for CD45^+^, F4/80^+^CD11b^+^, MHCII^lo^/CD206^hi^ macrophages that represented TAM_C3 macrophages with the levels being markedly higher in the T127 models. Olaparib induced a further decrease in CD45^+^, F4/80^+^CD11b^+^, MHCII^lo^/CD206^hi^ macrophages in the T22 single strain model that was abrogated in the T22 co-transplantation model (Fig. 2C).

This again supported the contention that the effect of T127 on the sensitivity of T22 to olaparib in the co-transplantation model could be dependent on the relative levels of TAM_C3 compared to the TAM_C0, TAM_C1 and TAM_C4 macrophage populations in T22 single strain to T22 co-transplantation models with those effects being accentuated in the presence of olaparib.

We thus explored mechanisms that could contribute to the marked differences between the TAM populations and the differential effects of olaparib on the TAM populations in the T127 and T22 models including the effects in co-transplanted tumors. CellChat, which infers cell-state specific signaling communications within scRNA-seq datasets^49^, was used to visualize ligand-receptor interactions between tumor cells and TAMs (Supplementary Fig. 2G–I, Supplementary Fig. 3A–C). Known interactions between macrophages and tumors such as Lgals9-Cd45, Csf1-Csf1r, Cx3cl1-Cx3Cr1, Ccl7-Ccr2 were captured by CellChat. We then sought ligand-receptor pairs that mediated communication between tumor cells and TAMs that were up-regulated in olaparib resistant models but not in olaparib sensitive models (Supplementary Fig. 3D). We then created an interaction count estimate by summing the interactions that were increased in the T22 co-transplantation model in the presence of olaparib compared to the T22 single strain model in the presence of olaparib and subtracting those that were decreased (see Methods, Supplementary Fig. 3E). Based on the interaction count estimate in the co-transplantation model only a Rps19-C5ar1 interaction between TAM_C3 and malignant cells demonstrated up-regulation in the olaparib resistant models but down-regulation in the olaparib sensitive models (Fig. 2D). Of the receptor transcripts, C5ar1 was markedly upregulated in TAMs by olaparib in both the T127 and T22 co-transplantation models but not in T22 single strain model (Fig. 2E). Based on this, we explored C5ar1 as a potential mediator underlying the ability of co-transplantation with T127 to render T22 resistant to olaparib. Accordingly, TAM_C3 were re-grouped based on their C5ar1 expression.

After re-grouping TAM_C3 into the C5ar1_high and C5ar1_low populations, expression of genes associated with anti-inflammatory activity was exclusively expressed by the C5ar1_hi_C3 group and the C5ar1 high subset lacked expression of genes associated with pro-inflammatory activity (Fig. 2F, G). In contrast, the C5ar1_lo_C3 subgroup did not express significant levels of genes associated with pro-inflammatory activity or with anti-inflammatory activity. Consistently with TAM_C3, C5ar1_hi_C3 was dominantly associated with a worse outcome in TCGA basal breast cancer (Supplementary Fig. 3F) and when assessing contributions of cluster top DEG to outcomes in TCGA basal breast cancer by multivariate analysis, C5AR1 was associated with poor outcomes independently (Supplementary Fig. 3G). In the olaparib sensitive model (T22_ss) but not the resistant models (both T127 models and T22_co), C5aR1_hi_C3 macrophages were decreased (Fig. 2H). The differential effect was most clearly apparent when the ratio of TAM_C1 to C5ar1_hi_C3 TAM was plotted (Fig. 2I).

One potential explanation for the change in numbers and phenotypes of TAMs in the T22 co-transplantation model was transit of TAMs from T127 to T22. In support of this contention, flow cytometry of peripheral blood cells demonstrated higher levels of MHC-II^lo^C5aR1^hi^ macrophages in blood from both T127 models and T22 co-transplantation model compared to blood from T22 single strain model (Fig. 2J and Supplementary Fig. 3H). The numbers of MHC-II^lo^C5aR1^hi^ macrophages present in blood were further increased by olaparib in both the T127 single strain and the T127 co-transplantation model (Fig. 2J and Supplementary Fig. 3H) with a modest decrease in the T22 single strain model.

Together, the identification of elevated levels of MHC-II^lo^C5aR1^hi^ macrophages in blood from both the T127 single strain and the T127 co-transplantation models and the further increase with olaparib was consistent with the transit of MHC-II^lo^C5aR1^hi^ macrophages from T127 to T22 in the co-transplantation model rendering the normally olaparib-sensitive T22 model olaparib-resistant.

### Olaparib induces rapid changes in macrophage subsets and communication in T22 single strain model

**Fig. 3:**
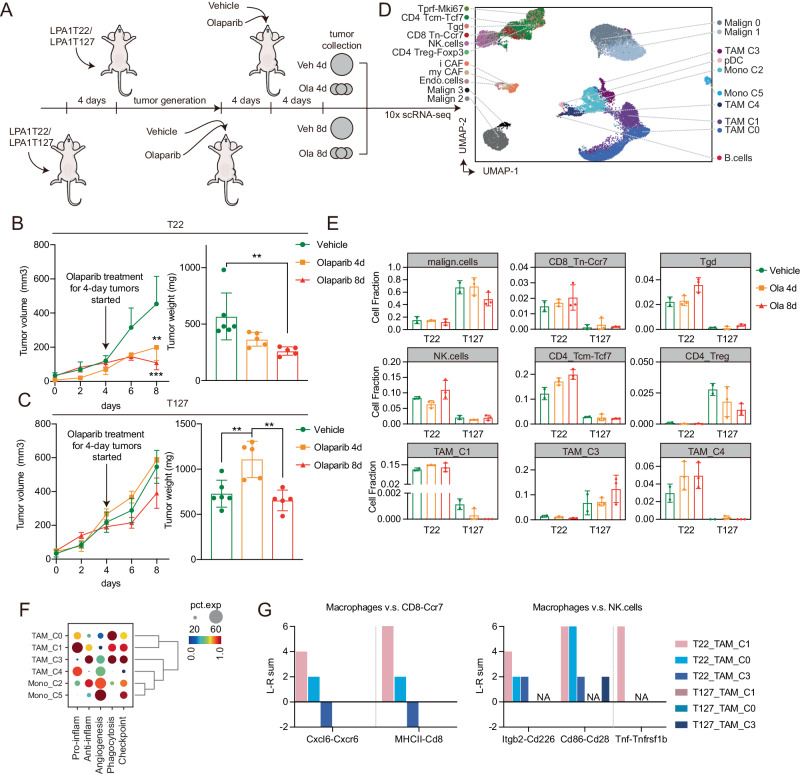
Effects of short-term olaparib treatment on tumor growth and immune cell contexture. **A** Design of the study in T22 and T127 models for olaparib treatment for 4 days or 8 days. 4d: 4 days, 8d: 8 days, **B** Growth curve (left) and weight of tumors at end of study (right) in T22 treated with olaparib (50 mg/kg, po, daily) for 4 days and 8 days. For vehicle treated, *n* = 6, and vehicle treated, *n* = 5. Data are presented as mean values±SD. *p* values are from one-way ANOVA. ** *P* < 0.01. **C** Growth curve (left) and weight of tumors at end of study (right) of T127 treated with olaparib for 4 days or 8 days. For vehicle treated, *n* = 6 and olaparib treated, *n* = 5. Data are presented as mean values±SD. *p* values are from one-way ANOVA. ** *P* < 0.01. **D** Stratification and cell-type identification of T22 and T127 tumors from mouse models indicated in **A**. Malignant cells from T127 (Malign_0, Malign_1) and T22 (Malign_2, Malign_3) show distinct cell clusters. Tcm: central memory T cells, Treg: regulator T cells, Tn: naive T cells, Tgd: γδT cells, Tprf: proliferative T cells, my_CAF: myofibroblastic cancer associated fibroblast cells, i_CAF: inflammatory cancer associated fibroblast cells, pDC: plasmacytoid dendritic cells. For vehicle treated, *n* = 2 and olaparib treated, *n* = 3. **E** Bar plot showing relative numbers of epithelial (high CNV cells), NK cells and i_CAF (in %) defined by cell lineage markers listed in (Fig. 1E) from the scRNAseq datasets in **D**. Data are presented as mean values ± SD. Statistical significance was not applicable due to the small size of each group. **F** Dot plot of summarized expression level of each pathway of indicated TAM clusters. Gene lists used for functional pathway scores are listed in Fig. 2F. **G** Ligand-receptor interaction count changes estimated by CellChat between each TAM cluster and CD8 naive T cells (left) or NK cells (right and defined by cell lineage markers indicated in Fig. 1E) after olaparib treatment. No interactions were noted in T127 likely due to the low number of T cells in T127. Source data and exact *p* values are provided as a Source Data file. Source data are provided as a Source Data file.

To capture effects of olaparib on the tumor microenvironment at earlier time points, we assessed T22 and T127 after 4 and 8 days of treatment with olaparib (Fig. 3A). To decrease batch effects, all tumors were harvested at the same time by transplanting the “4-day tumors” 4 days after the 8-day tumors (Fig. 3A). Tumors were allowed to establish for 14 days and then treated with olaparib and all tumors harvested on the same day for scRNA-seq analysis (Fig. 3A). More than 20,000 cells were available for further analysis following stringent cell selection of scRNA-seq data. Even the short treatment of T22 but not T127 with olaparib was sufficient to decrease tumor growth and tumor weight (Fig. 3B, C).

Using the cell lineage definition approach from Figs. 1 and 2, malignant T127 (Malign_0, Malign_1) and T22 (Malign2, Malign_3) once again formed separate clusters, whereas non-malignant cell types from the two tumor models merged (Fig. 3D and Supplementary Fig. 4A). Immune cells comprised a greater portion of the tumor at early (4 and 8 days, Fig. 3D) compared to late timepoints (see Fig. 1). NK cells and T cells were almost exclusively present in T22 tumors (Fig. 3E and Supplementary Fig. 4B, C). CD4 Treg cells were exclusively present in T127 tumors but not enriched after olaparib treatment (Fig. 3E). There were no statistically significant changes in B cell or CAF subtypes between olaparib treated and untreated samples of T22 or T127 tumors.

Consistently with long-term treatment, in T22 olaparib increased TAM_C1 cells by day 4 of olaparib treatment that persisted to day 8 with a modest decrease in TAM_C3 cells (Fig. 3E, Supplementary Fig. 4B, C) by day 4 of olaparib treatment that persisted to day 8 Moreover, TAM_C1 in short-term models, as in the long-term model, expressed genes associated with a pro-inflammatory phenotype while TAM_C3, again similar to the long-term model expressed genes associated with anti-inflammatory, phagocytosis and checkpoint inhibition characteristics (Fig. 3F and Supplementary Fig. 4D). Based on CellChat, Cell-cell communication among malignant cells, TAM_C1, NK cells, i_CAF, CD8_Tn.CCR7 and CD4_Tcm.Tcf7 cells was markedly increased by olaparib in the T22 models but not in the T127 models (Supplementary Fig. 5A, B). In the T127 model, communications between malignant cells and TAM_C3 was apparent and increased by olaparib throughout the treatment period (Supplementary Fig. 5A, B).

To quantify effects of olaparib on interactions between cell types, changes in ligand-receptor interaction count estimate induced by olaparib (see Methods) compared to vehicle were then visualized. The Rps19-C5ar1 interaction between malignant cells and TAM_C3 was transiently increased by 4-days of olaparib treatment in T22 models but decreased by day 8. In contrast, in T127 models, the Rps19-C5ar1 interaction between malignant cells and TAM_C3 was increased by olaparib across the timepoints (Supplementary Fig. 5C). In T22, olaparib increased Cxcl16-Cxcr6, MHCII-Cd8 interaction count estimate between TAM_C1 and CD8_Tn-Ccr7 cells but not between TAM_C3 and CD8_Tn-Ccr7 cells on both day 4 and day 8 (Fig. 3G and Supplementary Fig. 5D). Olaparib also induced an increase in interactions of Itgb2-Cd226, Cd86-Cd28 and Tnf-Tnfrsf1b between NK cells and TAM_C1 in the T22 but not in the T127 model (Fig. 3G and Supplementary Fig. 5E)^50^.

Together, these data are consistent with olaparib inducing a rapid increase in expression of genes associated with pro-inflammatory characteristics in TAM_C1 from T22 as well as increased communication between TAM_C1 macrophages and naive T cells and NK cells in T22 but not in T127.

### C5aR1 inhibition sensitizes co-transplanted T22 as well as intrinsically PARPi resistant T127 to olaparib

**Fig. 4:**
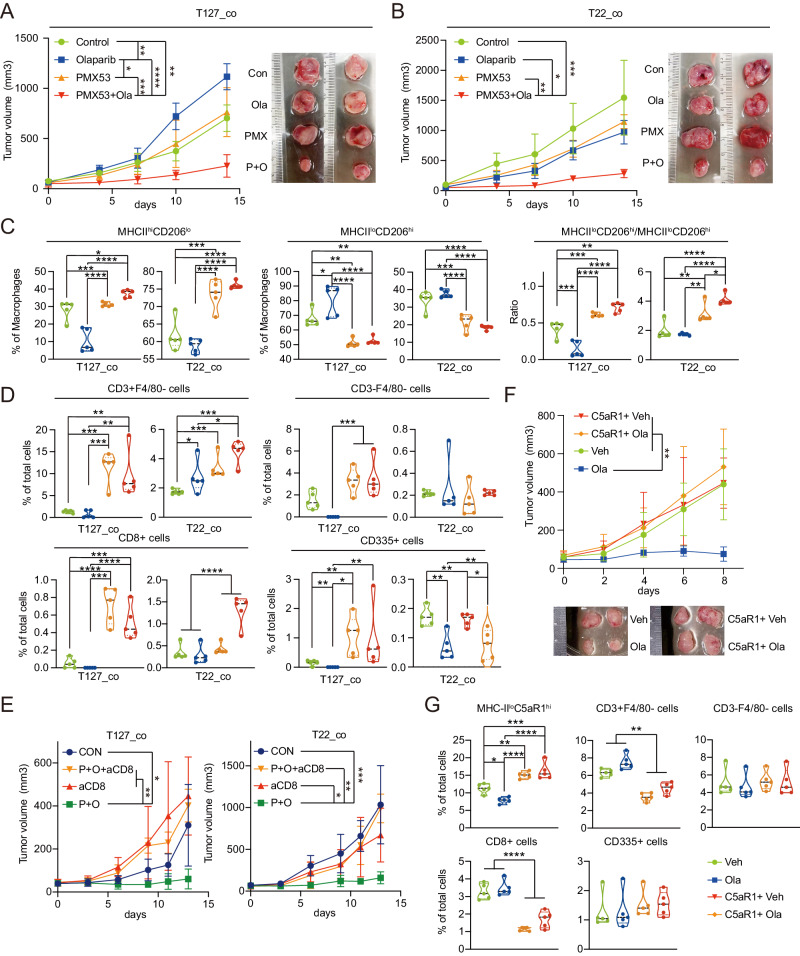
C5aR1 antagonist increases anti-tumor responses in co-transplantation model. **A** Growth curve of T127 (left) and images of representative T127 tumors (right) from co-transplanted T22 and T127 tumor models treated with control, olaparib, PMX53 or the combination for 14 days. *n* = 5. **B** Growth curve of T22 (left) and images of representative tumors of T22 tumors (right) from co-transplanted T22 and T127 tumor models treated with control, olaparib, PMX53 or the combination for 14 days. *n* = 5. (C) Fraction of pro-inflammatory (MHCII^hi^CD206^lo^) TAMs (left), pro-tumor (MHCII^lo^CD206^hi^) TAMs (center see gating strategy in Supplementary Fig. 2B–D) out of total macrophages (F4/80^+^CD11b^+^) and ratio of pro-inflammatory and pro-tumor TAMs (right) in each indicated treatment group assessed by flow cytometry. *n* = 5. **D** Fraction of CD3^+^F4/80^−^ cells (upper left), CD3^−^F4/80^−^ cells (upper right), CD3^+^CD8^+^ cells (upper right) and CD3^−^CD335^+^ cells (lower right) in each indicated group assessed by flow cytometry. NK cells were gated as CD3^−^F4/80^−^CD335^+^ population. *n* = 5. **E** Tumor growth curve of T127 (left) and T22 (right) of co-transplanted T22 and T127 tumor models treated with control, anti-CD8 (5 mg/kg, i.p., q.o.d.), PMX53 (3 mg/kg, s.c., q.o.d.) combined with anti-CD8 or the combination of anti-CD8, PMX53 and olaparib (50 mg/kg, o.g., q.d.) for 14 days. aCD8: anti-CD8. *P* + O: PMX53 combined with olaparib. *n* = 5. **F** Tumor growth curve of T22 in mice receiving (5×10^5^) C5aR1^hi^ cells treated with vehicle or olaparib for 8 days. Treatment started on the day after transfer of C5aR1^hi^ cells. *n* = 5. **G** Fraction of MHC-II^lo^C5aR1^hi^, CD3^+^F4/80^−^ cells, CD3^−^F4/80^−^ cells, CD3^+^CD8^+^ cells and CD3^−^CD335^+^ cells in each treatment group from **E** assessed by flow cytometry. *n* = 5. All data are presented as mean values± SD. *p* values are from one-way ANOVA. * *p* < 0.05, ** *P* < 0.01, *** *p* < 0.001, **** *p* < 0.0001. Source data and exact *p* values are provided as a Source Data file. Source data are provided as a Source Data file.

Our data suggested that C5aR1 was present at high levels in TAM_C3 macrophages and CellChat suggested that it was involved in communication between TAM_C3 macrophages and tumor cells. To determine whether C5aR1 could contribute to the intrinsic T127 PARPi resistance and the PARPi resistance conferred on T22 in the co-transplantation model, we inhibited C5aR1 in the presence and absence of olaparib in co-transplanted T22 and T127. C5aR1 was inhibited with PMX53 (a synthetic peptidic active complement C5a receptor antagonist with an IC50 of 20 nM)^51^ in murine models^31^. Strikingly, while olaparib and PMX53 monotherapy had limited activity, the combination of olaparib and PMX53 not only re-sensitized T22 to olaparib in the co-transplantation model but also controlled the intrinsically olaparib-resistant T127 both in terms of growth curves and tumor weight with no obvious change in body weight (Fig. 4A, B, Supplementary Fig. 6A–C).

In order to extend our finding to another syngeneic model and to the PARP1 selective AZD5305^52^, we treated the KPCA ovarian transplantation model^53^ with PMX53, olaparib, or AZD5305 and the combination of PMX53 and PARPi. Similar to the breast cancer models, significant growth control was observed with the combination treatment with no significant effects of monotherapies (Supplementary Fig. 6D).

In the T22 co-transplantation model, PMX53 increased the number of MHCII^hi^CD206^lo^ macrophages (that reflect TAM_C0, TaM_C1 and TAM_C4 see above) which was further increased by the combination of PMX53 and olaparib (Fig. 4C). Concurrently, PMX53 decreased the number of MHCII^lo^CD206^hi^ macrophages (that reflect TAM_C3) with the combination having a slight additional effect in the T22 co-transplantation model. PMX53 induced a marked and statistically significant increase in MHCII^hi^CD206^lo^/MHCII^lo^CD206^hi^ ratios that was further increased by the combination (Fig. 4C). In the T127 co-transplantation model, olaparib decreased the number of MHCII^hi^CD206^lo^ macrophages as seen previously (Fig. 4C, Supplementary Fig. 6E). PMX-53 alone had only modest effects on MHCII^hi^CD206^lo^ macrophages in T127, however, PMX53 reversed the decrease in MHCII^hi^CD206^lo^ macrophages induced by olaparib alone and indeed resulted in an increase in the number of MHCII^hi^CD206^lo^ macrophages. In T127, the effects on MHCII^lo^CD206^hi^ macrophages were the inverse of the effects on MHCII^hi^CD206^lo^ macrophages (Fig. 4C, Supplementary Fig. 6E). While olaparib increased the number of MHCII^hi^CD206^lo^ macrophages and PMX53 alone had little effect, the combination resulted in a marked decrease in the number of MHCII^lo^CD206^hi^ macrophages. The combination of effects on MHCII^hi^CD206^lo^ and MHCII^lo^CD206^hi^ macrophages resulted in a marked increase in the MHCII^hi^CD206^lo^/MHCII^lo^CD206^hi^ ratio in T127 that likely contributed to the response to the PMX53 and olaparib combination.

The effects on MHCII^hi^CD206^lo^ and MHCII^lo^CD206^hi^ macrophages were promulgated to changes in CD8 T cells and NK cells (Fig. 4D, Supplementary Fig. 6F). In the T22 co-transplantation model, the combination of PMX53 resulted in a statistically significant increase in CD3 and CD8 T cells with modest if any effects on NK cell numbers (Fig. 4D). In the T127 co-transplantation model that has very low levels of T cells without therapy, PMX53 induced an increase in CD3^+^ T cells that was maintained with the combination. The decreased NK cell levels in T127 after olaparib treatment was reversed by PMX53 (Fig. 4D). In support of the involvement of CD8 T cells in the response to the combination of PMX53 with olaparib, the efficacy of combination therapy was significantly mitigated by anti-CD8 treatment (Fig. 4E).

To directly test whether C5aR1 macrophages from T127 were involved in the transfer of resistance to T22 in the co-transplantation model, we isolated C5aR1^hi^ cells (that are mainly TAM_C3 from the blood of T127 tumor-bearing mice and transferred them to T22 tumor-bearing mice (Supplementary Fig. 6G). Remarkably, the C5aR1^hi^ cells from the blood of T127 tumor-bearing mice were sufficient to render T22 resistant to olaparib (Fig. 4F, Supplementary Fig. 6H, I). As expected, flow cytometry of T22 tumors demonstrated higher levels of MHC-II^lo^C5aR1^hi^ macrophages following C5aR1^hi^ cell transfer. Consistent with the scRNA-seq data of 8-day treated T22 tumors, a slight decrease of MHC-II^lo^C5aR1^hi^ pro-tumor macrophages after olaparib treatment was only observed in non-transferred models but not in C5aR1^hi^ cell transferred models. The proportion of CD3 and CD8 in T22 was significantly decreased by C5aR1^hi^ cell transplantation (Fig. 4G). In contrast, transfer of C5aR1^hi^ cells did not alter NK cell levels.

Together these data are consistent with C5aR1 playing a major role in the elevated MHCII^lo^CD206^hi^ macrophage fraction in T127 and T22 co-transplantation models. The decreased MHCII^lo^CD206^hi^ macrophage fraction in PMX53 and olaparib treated mice as well as an increase in CD8 T cells likely contributed to the tumor growth inhibition induced by the combination of PMX53 and olaparib in both T127 and T22 in co-transplantation models.

### C5aR1^hi^ macrophages educated with olaparib treated T127 alter T cell activity ex vivo

**Fig. 5:**
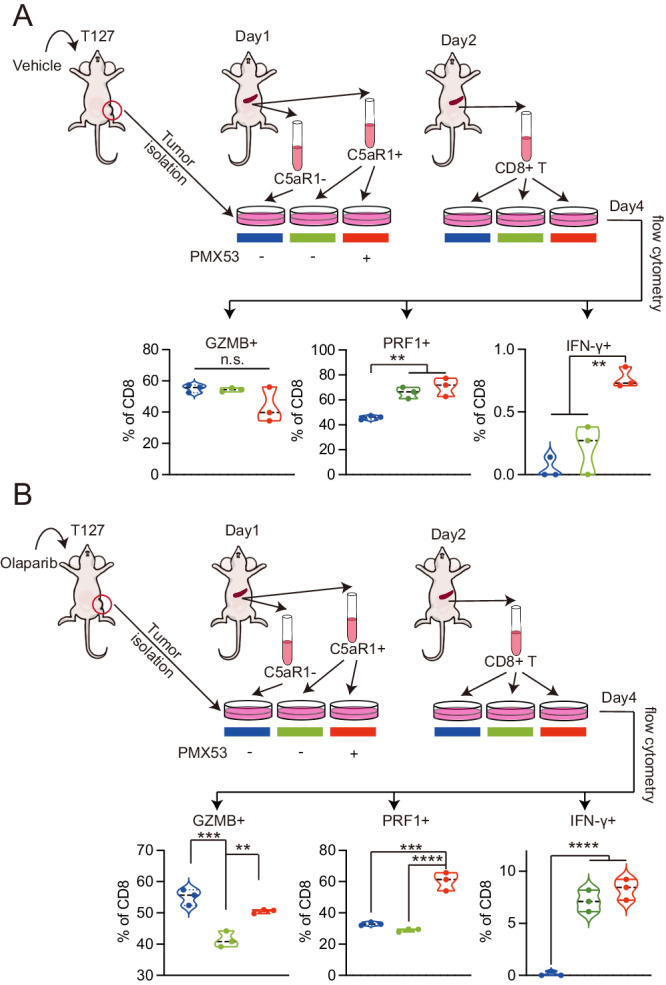
C5aR1 antagonist treated macrophages activate T cell cytotoxicity ex vivo. **A** Work flow of T127 tumor cells purified from vehicle control mice as described in Fig. 1, CD11b^+^C5aR1^−/+^ macrophages isolated from tumor naive mice and T cells from tumor naive mice ex vivo co-culture assay (see Methods for details). Briefly, on day 1, 1 × 10^6^ T127 tumor cells from vehicle treated mice were co-cultured with 5 × 10^5^ CD11b^+^C5aR1^−/+^ macrophages with and without PMX53 (40 nM). On day 2, CD11b^+^C5aR1^−/+^ macrophages were isolated and then co-cultured with 5 × 10^5^ isolated CD8 T cells. CD11b^+^C5aR1^−/+^ macrophages were isolated from CD11b^+^ positive splenocytes as described in Methods. Fraction of GZMB^+^, PRF1^+^, IFNγ^+^ CD8^+^ T cells in each indicated group from left assessed by flow cytometry. *n* = 3. Data are presented as mean values±SD. Comparison was done by one-way ANNOVA. * *p* < 0.05, ** *P* < 0.01, *** *p* < 0.001, **** *p* < 0.0001. **B** Work flow of T127 tumor cells purified from mice treated with olaparib as described in Fig. 1, CD11b^+^C5aR1^−/+^ macrophages isolated from tumor naive mice and T cells from tumor naïve mice ex vivo co-culture assay (see Methods for details). Briefly, on day 1, 1 × 10^6^ T127 tumor cells from olaparib treated mice were co-cultured with 5 × 10^5^ CD11b^+^C5aR1^−/+^ macrophages. On day 2, CD11b^+^C5aR1^−/+^ macrophages were isolated and then co-cultured with 5 × 10^5^ isolated CD8 T cells. CD11b^+^C5aR1^−/+^ macrophages were isolated from CD11b^+^ positive splenocytes. Fraction of GZMB^+^, PRF1^+^, IFNγ^+^ cells of CD8^+^ T cells in each indicated group from left assessed by flow cytometry. *n* = 3. Data are presented as mean values ± SD. Comparison was done by one-way ANNOVA. * *p* < 0.05, ** *P* < 0.01, *** *p* < 0.001, **** *p* < 0.0001. Source data and exact *p* values are provided as a Source Data file. Source data are provided as a Source Data file.

To investigate the effect of T127 educated C5aR1^hi^ macrophages on T cell function ex vivo, we first isolated T127 tumor cells from vehicle or olaparib-treated mice as described in Fig. 1. The purified T127 tumor cells were then incubated with CD11b^+^C5aR1^lo^ or CD11b^+^C5aR1^hi^ macrophages isolated from tumor naive mouse spleenocytes with and without PMX53 (40 nM) in a transwell system for 24 h (see Methods for details). We then co-cultured the pre-incubated macrophages (without PMX53) with CD8 + T cells isolated from tumor naïve mouse spleenocytes. C5aR1^lo^ and C5aR1^hi^ macrophages pre-incubated with T127 cells from vehicle treated mice did not induced marked differences in CD8 activation as indicated by granzyme B or IFNγ expression, albeit with a modest increase in perforin (PRF1) (Fig. 5A and Supplementary Fig. 6J). PMX53 did not alter granzyme or perforin expression but it did slightly increase IFNγ expression (Fig. 5A). In contrast, granzyme B (GZMB) and perforin expression was decreased by C5aR1^hi^ macrophages pre-incubated with olaparib-treated T127 tumor cells (Fig. 5B). For as yet unclear reasons, there was an increase in IFNγ expression in the CD8 T cells. The effects of the C5aR1^hi^ macrophages pre-incubated with olaparib treated T127 tumor cells on granzyme B and perforin expression was reversed by C5aR1 inhibition during the co-culture of T127 from olaparib-treated mice and the C5aR1^hi^ macrophages with no further induction of IFNγ compared to pre-incubation with olaparib-treated T127 tumor cells (Fig. 5B).

Next, we expanded our findings to the PARP1 selective AZD5305 (Supplementary Fig. 6K). We isolated T127 tumor cells from treatment naive mice. Purified T127 tumor cells were then treated with DMSO or AZD5305 for 96 h and then incubated with CD11b^+^C5aR1^hi^ macrophages isolated from tumor naive mouse spleenocytes with and without PMX53 (40 nM) in a transwell system for 24 h as described above. We then co-cultured the pre-incubated macrophages (without PMX53) with CD8^+^ T cells isolated from tumor naive mouse spleenocytes (Supplementary Fig. 6K). T127 tumor cells treated in vitro with AZD5305 and then cocultured in a transwell system with C5aR1 macrophages resulted in C5aR1 macrophages that suppressed T cell activation as assessed by positivity for GMZB, PRF1 or IFNγ (Supplementary Fig. 6K). This effect was reversed by PMX53 treatment. Thus treatment of T127 tumor cells with AZD5305 creates an immunosuppressive cell phenotype that is reversed by PMX53 treatment.

### C5aR1 contributes to TAM_C3 polarization

**Fig. 6:**
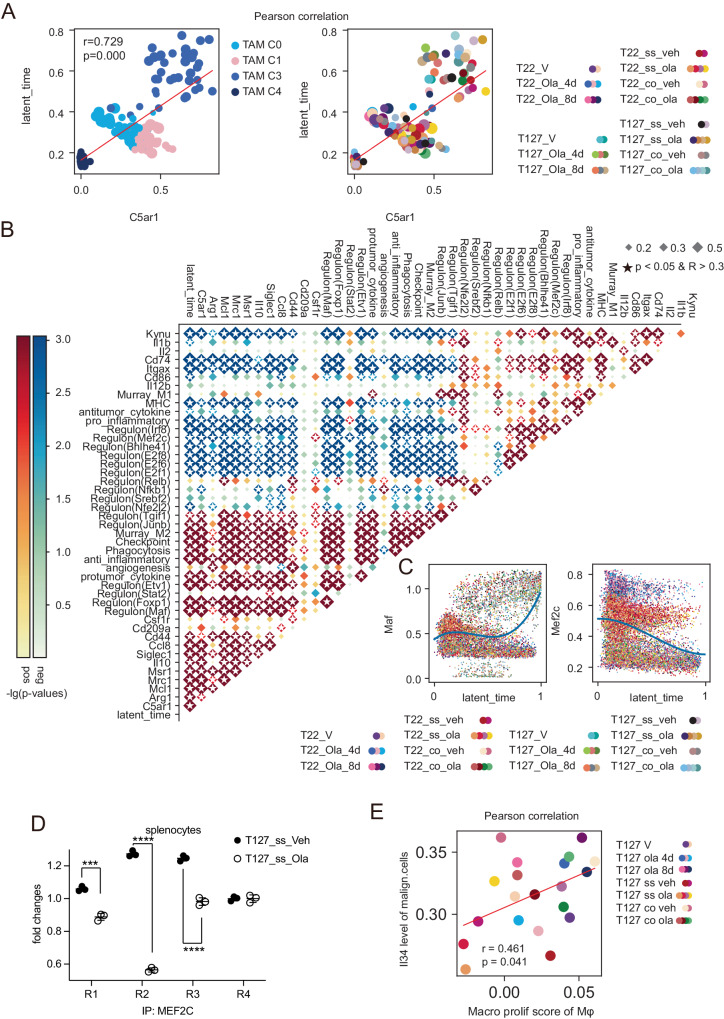
C5aR1 contributes to TAM_C3 polarization. **A** Correlation between latent time and C5ar1 expression in pooled macrophages including each sub-cluster of macrophages classified by reported markers from all tumors from Figs. 1 and  2. Cells are grouped in each sub-cluster in each treatment as indicated in the box to the right. Dot size indicates cell numbers in each group. ss: single strain transplantation models. co: co-transplantation models. veh: vehicle. ola: olaparib. *p* value was from Pearson analysis. **B** Heatmap showing correlation between macrophage functional markers, pathways and regulons enriched by SCENIC among the pooled macrophages from (**A**) by pseudo-bulk analysis. Gene lists used for functional pathway scores are listed in Supplementary Table 2. **C** Correlation between AUC of Maf mRNA relative expression versus latent time (left) and AUC of Mef2c mRNA relative expression versus latent time (right) in the population macrophages from **B**. Dots were colored by treatment as indicated in lower panel. ss: single strain transplantation models. co: co-transplantation models. Veh: vehicle. Ola: olaparib. **D** Splenocytes from T127 single strain models treated with vehicle or olaparib (50 mg/kg, po daily) as described in Fig. 1 were subjected to ChIP with anti-rabbit IgG or anti-MEF2C. Fold changes of targets were calculated by normalization to β-actin expression level after subtracting the background binding detected with normal rabbit IgG antibodies. *n* = 3. Data are presented as mean values±SD. Comparison between groups was done by two-sided Student’s *t* test. ** *P* < 0.01. **E** Correlation between Il34 mRNA expression of malignant cells versus the macrophage proliferative scores from the same tumors. Dots were colored by sample and subtype as indicated in right panel. ss: single strain transplantation models. co: co-transplantation models. V: tumors treated with vehicle for short term. veh: vehicle. ola: olaparib. *p* value was from Pearson analysis. Source data and exact *p* values are provided as a Source Data file. Source data are provided as a Source Data file.

In order to visualize the progression of cellular differentiation in context of transcriptome remodeling, we used scVelo RNA velocity densMAP analysis^54^ to develop a latent-time based model consistent with transition from TAM_C4 through TAM_C0 to TAM_C1 or TAM_C3 macrophages (Supplementary Fig. 7A). DensMAP uses cell density to develop trajectories and provides a stronger representation of cell state than UMAPs^55^. This is consistent with TAM phenotypes being relatively plastic^56–58^. C5ar1 expression correlated with the polarization latent-time axis (Fig. 6A). This suggests that C5ar1 may contribute to TAM_C3 polarization.

Subsequently, correlations between latent-time and macrophage subtype functional markers including C5ar1 were analyzed. Mrc1, Msr1, Ccl8, Il10, Siglec1 that have been associated with pro-tumor activity, pro-tumor cytokine production, anti-inflammatory, phagocytosis and checkpoint activities^59,60^ were significantly positively correlated with C5ar1 expression and latent-time as were the Msr1, Mrc1, Mcl1, Ccl8, Il10 and Siglec1 pro-tumor markers with protumor cytokine production, anti-inflammatory, phagocytosis and checkpoint activities^61^ (Fig. 6B). C5ar1 and latent-time were inversely correlated with Kyun, Cd74, MHCII, and genes associated with pro-inflammatory activity^62^ (Fig. 6B). Il1b was highly correlated with genes associated with antitumor cytokine production (Fig. 6B). This is consistent with C5ar1 inhibiting expression of genes associated with inflammatory and anti-tumor activities. To identify transcription factor(s) (TF) that could contribute to macrophage polarization, we applied Single-Cell Regulatory Network Inference and Clustering (SCENIC)^63^ and identified Mef2c, Junb, Tgif1 and Nfe2l2 as candidate transcription factors contributing to TAM_C1 polarization^43,64–69^, whereas Maf, Foxp1, Stat2 and Etv1 were candidate transcription factors contributing to TAM_C3 polarization (Supplementary Fig. 7B)^70–75^. C5ar1 expression was correlated with AUC of Maf, Foxp1 and Etv1 TAM_C3 macrophage regulon candidates but was inversely correlated with the AUC of each of four regulons that appear to contribute to TAM_C1 macrophage polarization. Furthermore, AUC of the proposed TAM_C1 macrophage regulons co-varied but were inversely correlated with the TAM_C3 macrophage regulons (Fig. 6B). C5ar1 was highly correlated with transcriptomes associated with protumor cytokine production. In support, the mRNA level of Maf gradually increased while Mef2c decreased along the latent-time axis (Fig. 6C).

MEF2C that has been implicated as a pro-tumor repressor showed the highest binding potential by JASPAR^76^. We thus explored whether Mef2c could contribute to C5ar1 expression. Multiple Mef2c consensus binding sequences are present in the C5ar1 promoter region (Supplementary Fig. 7C, D). ChIP-qPCR with MEF2C antibodies and C5ar1 promoter region primers in splenocytes from the T127 single strain models showed a significant decrease in binding of MEF2C in 3 out of 4 C5ar1 promoter regions in the presence of olaparib (Fig. 6D). This is consistent with the increase in C5ar1 in TAM_C3 macrophages and furthermore in the increase in TAM_C3 macrophages in olaparib-treated T127.

Both IL34 and CSF1 have reported to promote macrophage proliferation. Since proliferating TAM_C0 macrophages appeared to be a transitional step to TMA_C1 and TAM_C3 macrophages (Supplementary Fig. 7A), we then determined whether the proliferative TAM fraction was associated with Il34 and Csf1 mRNA levels in malignant cells. Interestingly, malignant cell Il34 but not Csf1 expression in T127 correlated with the higher macrophage proliferative score (Fig. 6E and Supplementary Fig. 7E). In support of a role for IL34 in the transition of TAM_C0 macrophages to TAM_C3 macrophages, IL34 up-regulated C5aR1 expression in bone marrow derived myeloid cells (Supplementary Fig. 7F).

Together, C5aR1 is associated with activation of the TAM_C3 transcriptional program that appears to be increased by loss of MEF2C repression.

### C5aR1 is associated with T cell dysfunction in human TNBC tumors

**Fig. 7:**
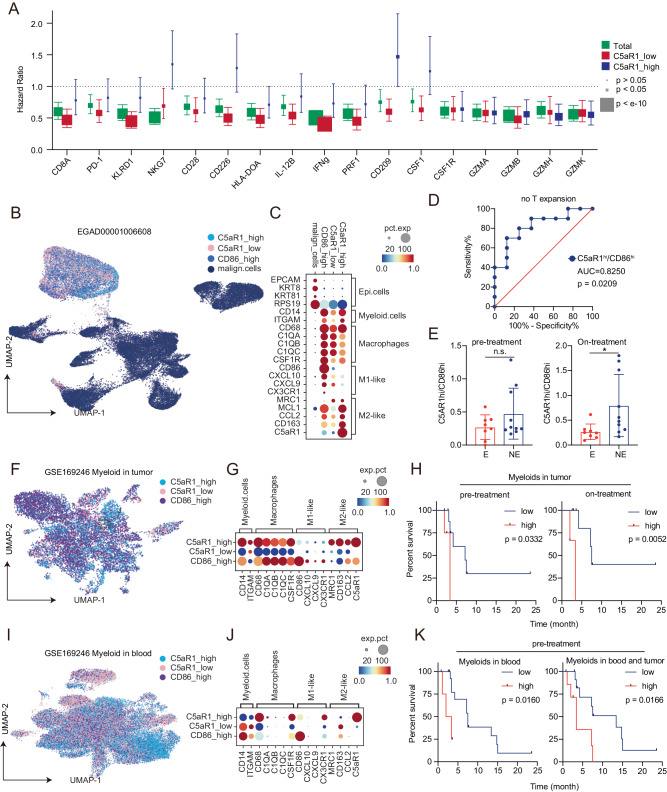
Correlation of C5aR1 and anti-tumor immune response in human TNBCs. **A** Dot chart of Hazard Ratio of indicated genes in a TNBC database (953 patients, version 2022.07) (green)^77^, C5aR1^hi^ cohort (blue) or C5aR1^lo^ cohort (red). Cutoff of C5aR1 cohorts were set as median expression of C5aR1. *p* value are from Log-rank test. In the dot plots, the center points show the hazard ratio (HR) and lines represent 95% confidence interval (CI). **B** Stratification and cell-type identification of human tumor cells and TAMs from EGAD00001006608^46^. Malignant cell annotation was adopted from the original publication. High= top 25%. *n* = 18. **C** Dot plot showing gene expression of cell-type specific marker and functional genes. (**D**) ROC curve demonstrating the ability of C5aR1^hi^/CD86^hi^ ratio to predict patients who did not achieve T cell expansion after anti-PD1 treatment. *p* values are from Receiver operating characteristic curve (ROC). *n* = 18. (**E**) Bar chart of the C5aR1^hi^/CD86^hi^ ratio in pre and on therapy biopsies of patients who did **E** or did not (NE) show T cell expansion. Data are presented as mean values±SD. *p* values are from two-sided Student’s *t* test. * p < 0.05. n_E_ = 8, n_NE_ = 10. **F** Stratification and cell-type identification of human TNBC TAMs in tumors from GSE169246^44^. Cell type was classified as indicated in **B**. *n* = 14. **G** Dot plot showing gene expression of lineage specific markers in tumor myeloid cells. **H** Kaplan-Meier relapse-free survival curves of GSE169246^46^ patients based on C5aR1^hi^/CD86^hi^ ratio in pre- (left) and post- (right) treatment tumor cells. High= top 25%. Survival information was from the original publication^44,86^. *p* values are from Mann-Whitney test. *n* = 14. **I** Stratification and cell-type identification of human TNBC blood myeloid cells from GSE169246. Cell type was defined as indicated in **B**. *n* = 20. **J** Dot plot showing gene expression of lineage specific markers in blood myeloid cells. **K** Kaplan-Meier relapse-free survival curves of patients from GSE169246 based on C5aR1^hi^/CD86^hi^ ratio in pre-treatment blood (left, high= top 25%) and in either blood or tumor (right) myeloid cells. Survival information was from the original publication^44^. *n* = 20. *p* values are from two-sided Mann-Whitney test. Source data and exact *p* values are provided as a Source Data file. Source data are provided as a Source Data file.

To determine whether C5aR1 could predict outcomes in TNBC patients, we assessed the association of markers of immune activation with outcomes in human TNBC tumors with high or low C5aR1 levels. Strikingly, CD8A, PD-1, KLRD1, NKG7, CD226, CD96, HLA-DOA, IL-12B, IFNG and PRF1, which have been associated with immune infiltration and improved outcomes in TNBC^77^, predicted improved outcomes in a C5aR1^lo^ cohort but not in a C5aR1^hi^ cohort (Fig. 7A). This suggests that high C5aR1 levels limit the ability of immune cells to contribute to efficient anti-tumor activity. CD209 and CSF1, which mediate macrophage phagocytosis and activation^78^, also correlated with better outcomes in the C5aR1^lo^ cohort but not in the C5aR1^hi^ cohort (Fig. 7A). Interestingly, granzymes (GZMs) that contribute to anti-tumor effects by inducing cell death^79^ predicted outcome independent of C5aR1 levels consistent with C5aR1 playing a role in the development of effector T cells rather than in their activity (Fig. 7A). CSF1R levels were predictive of a good patient outcome independent of C5aR1 levels for reasons that remain to be determined. These data are consistent with a model wherein C5aR1 limits the efficacy of anti-tumor immune activity.

ICB targeting of immunoregulatory pathways leads to re-activation of immune response in a number of tumor lineages resulting in improved patient outcomes^80^. However, ICB has shown modest activity in TNBC clinical studies^81–83^. To determine whether C5aR1 expression on macrophages has the potential to alter response to ICB, we assessed correlations between C5aR1 expression in a breast cancer scRNA-seq dataset of patients receiving anti-PD-1 where treatment response was estimated by T cell expansion^46^. Myeloid cells were annotated as C5aR1^hi^ and CD86^hi^ (based on high expression of CD86 and no expression of C5aR1, CD163, MRC1) and “C5aR1^lo^” that did not express high levels of C5aR1 or CD86 (Fig. 7B, C). Strikingly a high C5aR1^hi^/ CD86^hi^ ratio of the on-treatment sample predicted a lack of T cell expansion in response to anti-PD-1 (AUC of 0.8250, p = 0.0209) (Fig. 7D, E). As expected, a high CD163^hi^/ CD86^hi^ ratio or CD163^hi^/ CD86^hi^ ratio did not show similar prediction power (Supplementary Fig. 8A).

We analyzed an additional scRNA data set from patients treated with chemotherapy with or without atezolizumab^44^ (Fig. 7F–H). In 14 TNBC patients with tumor samples available, a high tumor C5aR1^hi^/CD86^hi^ ratio was significantly correlated with a worsened progression free survival in both pre- (PFS, *p* = 0.0332) and post- (PFS, *p* = 0.0052) treatment samples (Fig. 7H). Consistent with our observation that C5aR1 positive macrophages are elevated in blood from mice with intrinsically PARPi resistant T127 tumors, an elevated C5aR1^hi^/CD86^hi^ ratio in blood of 20 patients was also significantly associated with a worsened PFS (*p* = 0.0160) but only in pre-treatment samples (Fig. 7J, K). This was further supported by the observation that patients with a high C5aR1^hi^/CD86^hi^ ratio in either pre-treatment blood or tumor cells was significantly associated with a worsened PFS (*p* = 0.0166) (Fig. 7L). Again, the similar prediction power was not observed with a high CD163^hi^/ CD86^hi^ ratio or CD163^hi^/ CD86^hi^ ratio (Supplementary Fig. 8B–E).

Taken together, these data indicate that elevated C5aR1 levels and C5aR1 on macrophages is associated with ICB resistance in human cancer patients.

## Discussion

We demonstrate that C5aR1 high macrophages mediate resistance to PARPi and further that a high C5aR1 high TAM fraction predicts resistance to PARPi. C5aR1-positive macrophage-mediated PARPi resistance can be transferred to distant sites suggesting that the ability of a tumor subclone to generate C5aR1 high macrophages could mediate resistance of normally PARPi-sensitive subclones at disparate sites in a solid tumor or potentially at metastatic tumor sites.

Several studies have identified correlations between pro-tumor macrophages and pro-inflammatory/pro-tumor macrophage ratios and patient prognosis as well as resistance to a number of therapies including PARPi in BRCA mutant breast cancer models^31,32,84,85^. However, this is potentially simplistic as macrophages are plastic and macrophage subpopulations recently identified in scRNA Seq analysis express a mixture of gene expression patterns associated with both pro-inflammatory and anti-inflammatory/pro-tumorigenic activity^25,26^. A number of clinical trials have targeted macrophage function through inhibition of CSF1R activation and signaling^31,86–91^. In contrast to CSF1R studies, our data suggests that targeting C5aR1 selectively attenuates pro-tumor function of macrophages while retaining macrophage populations with dominant gene expression patterns associated with a pro-inflammatory functions thus resulting in a high pro-inflammatory macrophage/pro-tumor macrophage ratio. This is associated with a CD8 T cell dependent decrease in tumor growth in PARPi-resistant tumors. C5aR1 has previously been implicated in mediating pro-tumor macrophage polarization and in creating a pro-tumor microenvironment^92^. C5aR1^hi^ macrophages have been suggested to increase tumor progression as well as suppress T cell cytotoxicity in squamous carcinoma^93^, colon cancer^94^, cervical cancer^95^ and breast cancer^96^. Our data supports the contention that blocking C5aR1 would target pro-tumor macrophages while sparing pro-inflammatory function of macrophages suggesting that targeting C5aR1 could benefit patients receiving PARPi and potentially other therapies. In support of this contention, high C5aR1 levels as well as C5aR1 positive macrophages in human patients are associated with resistance to immune checkpoint blockade.

A recent study suggested that C5aR1^+^ neutrophils contribute to poor breast cancer outcomes by promoting glycolytic capacity through secretion of IL1β and TNFα^97^. This is similar to our study, where we identified a correlation between C5aR1 levels and an increase in pro-tumor cytokine gene expression. However, no Fut4 expression which encodes CD15 or Cd177 was detected in our datasets indicating of lack of capture of neutrophils in our scRNA Seq analysis likely due to their fragility during the tumor isolation process.

The mechanisms regulating C5aR1 expression in macrophages have not previously been elucidated. Using RNA velocity trajectory analysis, transcriptomic profiling and ChIP-PCR, we have implicated MEF2C in suppressing C5aR1 expression. MEF2C has previously been implicated in repressing pro-tumor macrophage function while promoting pro-inflammatory macrophage polarization^64,98^. This is consistent with our trajectory analysis that implicates C5aR1 in the transition whereby macrophages express genes associated with pro-tumor functions. The association of C5aR1 and MEF2C expression with the expression of a number of other TFs suggests that they may also contribute to the regulation of pro-tumor macrophage function potentially through C5aR1.

We and others have demonstrated that PARPi can activate STING leading to interferon production and functional adaptive immune responses^27,32,99–101^. Interestingly, STING agonists can polarize TAMs to a more pro-inflammatory state with a consequent improvement in responses to PARPi^32^. This would be compatible with PARPi inducing STING activation in T22 but not in T127. Indeed, we have previously demonstrated that the KRAS mutation that is present in T127 limits PARPi induced STING activation through inhibition of accumulation of double stranded DNA (dsDNA) in the cytosol and further that inhibition of MEKi institutes accumulation of cytosolic dsDNA, STING activation and production of an effective immune response^27^. Our previous studies implicated a population of MDSC/macrophages in PARPi resistance in T127. The current studies extends these observations to demonstrated that a C5aR1 high macrophage population not only mediates PARPi resistance in T127 but is able to transfer PARPi resistance to the normally T22 PARPi-sensitive tumor at a distance.

In summary our studies have implicated C5aR1 high macrophages in mediating both local and distant therapy resistance to PARPi and likely other therapeutic mediators including ICB. Our studies further credential C5aR1 as a potential therapeutic target that provides an approach to selectively deplete pro-tumor macrophage function while sparing anti-tumor macrophage function resulting in CD8 T mediated inhibition of tumor growth. Moreover, our studies suggest that inhibition of C5aR1 as a strategy to deplete pro-tumor macrophage function to sensitize tumors to PARPi as well as with a broader range of therapeutic agents warrants exploration in the clinic.

## Methods

### Murine derived syngeneic transplantable (MDST) mouse tumor models

For MDST assays, female FVB mice at 6 weeks of age were purchased from the Jackson Laboratory. Animal experiments were performed in the AALAC approved Animal Facility, Knight cancer Institute, Oregon Health & Science University under IACUC protocol: Combination Therapy Targets Adaptive Resistance in Cancer (TR01_IP00002062). Mice were housed 5 to a cage with ad libitum access to food and water in 20 °C ambient temperatures, 40–50% humidity and a 12-h light/12-h dark cycle. LPA1-T22 and LPA1-T27 have been extensively characterized previously. Both are homologous recombination competent based on not demonstrating mutations of 33 genes involved in homologous recombination and by the ability to form RAD51 foci in response to DNA damaging agents^3,34,102^ (Supplementary Fig. 1A). 25mm^3^ LPA1-T22 (T22) or LPA1-T127 (T127) tumor chunks were orthotopically transplanted into the mammary fat pad. 10 and 14 days after tumor implantation for T127 and T22 respectively, mice were first assigned with individual numbers and then assigned to treatment groups (*n* > 5) using an online random number generator (http://www.graphpad.com/quickcalcs/randomize1/). For co-transplantation models, due to differences in growth rates, T127 tumors were transplanted in the opposite mammary fat pad when the T22 tumor reached 20mm^3^ (~14 days after T22 transplantation) as demonstration in Fig. 1A. For short term treatment assays, mice were treated with vehicle or olaparib (50 mg/kg, oral gavage, daily, Selleckchem, #S7110) for 4 or 8 days. For long-term treatment assays, with the C5aR1 antagonist, mice were treated with vehicle with PMX53C (3 mg/kg, subcutaneously, every other day, starting 7 days before olaparib treatment and continuing throughout the treatment protocol, 5697, Toris) or olaparib (50 mg/kg, oral gavage, daily, Selleckchem, #S7110) or PMX53 (3 mg/kg, subcutaneously, every second day, starting 7 days before olaparib treatment and continuing throughout the treatment protocol, HY-106178, MedChemExpress) or combination of olaparib and PMX53 for 20 days. For CD8 depletion assays, mice were treated with vehicle, with PMX53C (3 mg/kg, subcutaneously, every second day, starting 7 days before olaparib treatment and continuing throughout the treatment protocol, 5697, Toris) or IgG (1 mg/kg, intra-peritoneal, every second day, starting 7 days before olaparib treatment and continuing throughout the treatment protocol, BE0089, BioXcell) or combination of olaparib (50 mg/kg, oral gavage, daily, Selleckchem, #S7110) and PMX53 (3 mg/kg, subcutaneously, every second day, starting 7 days before olaparib treatment and continuing throughout the treatment protocol, HY-106178, MedChemExpress) or anti-CD8 (5 mg/kg, intraperitoneal, every second day, starting 7 days before olaparib treatment and continuing throughout the treatment protocol, BE0004-1, BioXcell) for 13 days. Tumors were measured by calipers every 2 or 4 days. Tumor volume was calculated according to a modified ellipsoid formula V = 1/2 × (Length × Width^2^). Mice were euthanized before reaching maximum tumor size of 2000 mm^3^ according to IACUC guidelines. Mice were isoflurane anesthetized and tumors were collected freshly for single cell RNA sequencing library construction, or cryopreserved as single cell suspension, or fixed overnight in 10% formalin, stored in 70% ethanol followed by paraffin embedding for future analysis.

### KPCA mouse tumor models

The KPCA syngeneic mouse tumor cell line was a gift Dr Weinberg^53^. Female C57BL/6 mice at 6 weeks of age were purchased and housed the same as indicated above under IACUC protocol. The KPCA cell line was cultured as described previously^53^ and 1 × 10^7^ cells were resuspended in 50ul of Matrigel mixed with PBS (1:1) and then injected subcutaneously in the flank of 48 C57BL/6 mice. After 7 days of tumor growth, mice were assigned to treatment groups (*n* > 5) using an online random number generator (http://www.graphpad.com/quickcalcs/randomize1/). Mice were treated with vehicle with PMX53C (3 mg/kg, subcutaneously, every other day), starting 7 days before olaparib and continuing throughout the treatment protocol or AZD5305 (Toris cat #5697) or olaparib (50 mg/kg, oral gavage, daily, Selleckchem, cat #S7110) or AZD5305 (0.4 mg/kg, o.g., daily) or PMX53 (MedChemExpress # HY-106178 3 mg/kg, subcutaneously, every second day), starting 7 days before olaparib treatment and continuing throughout the treatment protocol) or combination of PMX53 and the two PARP inhibitors respectively. Tumor volume was calculated according to a modified ellipsoid formula V = 1/2 × (Length × Width^2^). Mice were euthanized before reaching maximum tumor size of 2000 mm^3^ according to IACUC guidelines. Mice were isoflurane anesthetized and tumors were cryopreserved as single cell suspension, or fixed overnight in 10% formalin, stored in 70% ethanol followed by paraffin embedding for future analysis.

### Murine bone marrow-derived macrophage cell isolation

Mouse bone marrow-derived macrophage cells were derived from bone marrows isolated from FVB female mouse (8–10 weeks old) thighbone according to an established protocol^103^.

### Flow cytometry assays

Cells were isolated with gentleMACS kits (Miltenyi Biotec, #130-096-730). Cells were washed with cold staining buffer (Biolegend, #420201), followed by resuspension of 10^6^ cells in 100 μl cold staining buffer (Biolegend, #420201) with PI (Biolegend, #421301, 1:20) or Calcein Violet AM (Invitrogen, C34858, 0.1 μg) as live/dead cell indicators. Cells were stained with CD45-BV711 (Biolegend, 103147, 0.125 μg), CD3-APC (Biolegend, 100236, 0.125 μg), CD8-AF532 (Invitrogen, 58-0081-80, 0.125 μg), CD335-PerCP-efluor710 (Invitrogen, 46-3351-82, 0.125 μg), F4/80-BUV563 (Invitrogen, 365-4801-82, 0.25 μg), I-A/I-E-APC-Cy7 (Biolegend, 107627, 0.0625 μg), CD206-PE-eFluor610 (Thermo, 61-2061-82, .0625 μg) antibody, secondary goat anti-rat antibody conjugated with Alexa Fluor 555 (Thermo, A-21434, 1:500) and C5aR1 (Santa Cruz, sc-271949, 0.5 μg) for 30 min on ice. For CD8 T cell activation assays, after incubation of PI, CD45, CD3 and CD8 as indicated above, cells were permeabilized (eBioscience, 00-8333-56) and then incubated with the following antibodies GZMB-AlexaFluor594 (Fisher, IC2906T100UG, 0.5 μg), PRF1-PE (eBioscience, 12-9392-82, 0.5 μg) and IFNγ-BUV395 (eBioscience, 363-73311-82, 0.5 μg). After 3 washes with cold staining buffer, cells were analyzed by Aurora Cytek Analyzer. Values were reported as relative fluorescence intensity subtracted by IgG signal as background. Cells were gated by FSC-H vs FSC-A and SSC-H vs SSC-A and PI negative as live singlet cells. Single color controls were used for compensation. All analysis was performed in FlowJo (v10.6.1).

### C5aR1 high cell sorting and transfer

Peripheral blood from T127 carrying FVB mice was collected through orbital sinus of isoflurane anesthetized mice. Blood was collected with EDTA blood collection microtubes. After serum aspiration, blood was diluted with PBS and put through density centrifugation with Ficoll. After carefully extracting the layer of the mononuclear cells, cells were washed three times with PBS. CD11b positive cells were isolated by EasySep Mouse CD11b positive selection Kit II (STEMCELL, 18970). The CD11b positive cell pool was incubated with primary C5aR1 antibody at 3 μg/ml in PBS with 2% FBS. Secondary antibodies conjugated with Biotin were added 1.2 μg/ml in PBS with 2% FBS. Magnetic column based cell sorting was performed by following manufacture’s manual of EasySep Biotin Positive Selection Kit II (STEMCELL, 17683). The purity of positive cells was assessed with flow cytometry. 5*10^5^ C5aR1^hi^ cells in 100 μl selection buffer or only 100 μl selection buffer was transferred to FVB mouse 2 weeks after T22 transplantation through tail vein injection. Olaparib treatment started on the second day after C5aR1^hi^ cell transplantation with the same dose indicated above.

### CD8 T cell ex vivo activation assay

C5aR1^hi^ macrophages cells were isolated from spleen of tumor naive FVB mice as indicated above. C5aR1^−^ macrophages were the cells remaining after C5aR1 selection of the CD11b positive population. T127 tumor cells from mice treated with or without olaparib for 20 days as indicated in Fig. 1 were disaggregated and then depleted of CD45 positive hematopoietic cells with EasySep Mouse CD45 positive selection kit (STEMCELL, 18945). CD8 T cells were isolated from spleen of tumor naive FVB mice following the manufacture’s manual of EasySep Mouse CD8 T cell isolation Kit (STEMCELL, 19853). On day 1, 1×10^6^ T127 tumor cells from in vivo vehicle or olaparib treated mice were co-cultured with 5×10^5^ CD11b^+^C5aR1^−/+^ macrophages in a 0.4μm 24-well transwell plate (macrophages in the upper chamber and T127 cells in the lower chamber). On day 2, CD11b^+^C5aR1^−/+^ macrophages were isolated from the upper chamber, washed by PBS 3 times and then co-cultured with 5×10^5^ isolated CD8 T cells. After two days of co-culture, CD8 T cell activation was assessed by multi-color flow cytometry with antibodies to CD8, granzyme B (GZMB), perforin (PRF1) and INFγ. Golgi Plug Transport Inhibitor (Fisher, 15847968, 1:1000) was added 4 h before flow cytometry.

### ChIP-qPCR

Chromatin immunoprecipitation (ChIP) assays were performed with a Simplechip Enzymatic Chromatin IP kit (Cell Signaling Technology, #9003 S) following the product manual. Briefly, isolated splenocytes were crosslinked with 1% formaldehyde (Sigma, #47608-250ML-F). After cell lysis, nuclei were extracted and subjected to sonication for chromatin fragmentation. Chromatin samples were incubated for 4 h with rotation at 4 °C after adding anti-MEF2C antibody (Cell Signaling Technology, #5030 S). Antibody–chromatin complexes were pulled down by magnetic protein A/G beads. Purified DNAs were quantified by real-time PCR. Primers are listed in the Supplementary Table 1.

### Single cell RNA sequencing library construction

MDST tumors were disaggregated by gentleMACS kits (Miltenyi Biotec, #130-096-730), and incubated in TotelseqA hashtag antibodies (Biolegend, #394601, #394603, #394605, #394607, #394609, #394611, #394613, #394615) following the manufactural instructions. Live cells were isolated by EasySep Dead Cell Removal (Annexin V) Kit (STEMCELL Technologies, #17899). Single cell suspensions were processed according to 10xGenomics scRNAseq sample preparation protocol (Chromium Single Cell 3’ v3.1 Reagent Kit, 10xGenomics). Briefly, single cell suspensions with 2×10^6^ live cells were incubated with 100 μL antibody based hashtag oligos (10ug/ul) for 30 min on ice. After washing 3 times with 3 ml PBS with 10% FBS, 5×10^5^ live hashtagged cells were pooled together followed with dead cell removal column (STEMCELL, 17899) to enrich live cells. Sequencing was done by Novaseq 6000. 10000 cells were targeted for each sample.

### Single cell RNA sequencing data analysis

Single cell RNA sequencing data generated by 10xGenomics was processed by Cell Ranger count pipeline (v6.0.0) with command argument: “include introns” to filter low-quality reads and align reads to mouse reference genome (GRCm38, vM32), assign cell barcodes and generate unique molecular identifiers (UMI). Output BAM files were further processed by Velocyto to generate LOOM files containing UMI of spliced RNA, unspliced RNA and ambiguous as separate matrices^104^. Cells with fewer than 1000 genes detected (UMI > 1000) or with more than 20% UMIs from mitochondrial genes were excluded by SCANPY^105^. Doublets were removed by Scrublet with the expected doublet rate of 6 and doubletScore larger than 95%^106^. Filtered data with 4000 highly variable genes (HVG) were then normalized and scaled by SCANPY and scVelo^54^ to remove unwanted source of variance such as batch effects and cell cycle effects. Single cell sample demultiplexing was done by unsupervised Louvain clustering of cell multiplexing oligo matrix with a resolution of 1.2 and the Louvain communities with exclusively unique cell multiplexing oligo captured being assigned to their related treatment groups. Unsupervised clustering of the gene expression matrix was performed by Leiden cluster module with a resolution of 2 in SCANPY. Pathway scores were calculated with the genes listed in Supplementary Table 2 with the score module of SCANPY. Density preserving dimensionality reduction was done by densVis package with dens_lambda = 0.7^55^. Regulon enrichment was done by pySCENIC with default parameters^63^. Ligand-receptor based cell-cell communication was done by CellChat among genes expressed by more than 10% cells and among the cells with gene expression of top 80%^107^. Ligand-receptor pair database was downloaded from CellphoneDB^108^.

### Cell subtype assignment

Cell annotation was done according to the cell markers listed in Fig. 1E^37–40^. Briefly, cells with Cd45, Cd3, Cd4 and Tcf expression were assigned as central memory T cells. Cells with Cd45, Cd3, Cd4 and Foxp3 expression were assigned as regulatory T cells. Cells with Cd45, Cd3, Cd8 and Ccr7 expression were assigned as naive CD8 T cells. Cells with Cd45, Cd3 and Mki67 expression were assigned as proliferative T cells. Cells with Cd45, Ncr1 and Nkg7 expression were assigned as NK cells. Cells with Cd79a and Cd19 expression were assigned as B cells. Cells with Clec10a, Siglec and Cd300c were assigned as plasmacytoid dendritic cells. Cells with Dcn or Acta2 were assigned as fibroblasts. Cells with Epas1 expression were assigned as endothelial cells. Cells with copy number variations (CaSpEr) were assigned as malignant cells. Cells with Itgam, Cd14 and Adgre1 were assigned as monocytes or macrophages. Monocytes, macrophages and fibroblasts were further re-clustered by unsupervised Leiden clusters following by annotation with markers previously reported for different subsets and the top DEGs for each of the Leiden clusters^37,38,40^.

### Label transfer

Label transfer was done for macrophage phenotype validation. Myeloid raw counts of a pan-cancer tumor microenvironment project (GSE154763) were integrated with a reference based integration workflow in Seurat. In brief, datasets were first processed with *SCTransform*, and then transfer anchors were found by *FindTransferAnchors* followed by integration with *IntregrateData*. After integration of the pan-cancer myeloid datasets, the label information of pan-cancer was transferred by *TransferData* to our myeloid datasets with another set of transfer anchors between pan-cancer myeloid datasets and our myeloid datasets identified by *FindTransferAnchors*. Predicted labels with prediction score less than 0.35 were regarded as insufficient confidence as labels.

### Cell cross annotation by scGPT

The human pan-cancer myeloid dataset was accessed from the Gene Expression Omnibus (GEO) database using accession number GSE154763. Cross validation of mouse myeloid sub-clusters and human pan-cancer tumor myeloid clusters were down using scGPT^45^. Mouse normalized datasets were then log transformed and binned before model fine-tuning with default parameters in scGPT. The training datasets contained 25408 cells and 2425 genes. The query datasets contained 9748 cells and 3000 genes. 4000 HVGs were selected during data processing.

### Interaction count estimate

To estimate interactions between two different conditions containing independent replicates, we developed an “interaction count” estimate formula. Each tumor (a) from condition A is compared with each tumor (b) from condition B resulting in a×b comparison pairs. The interaction count estimate is then calculated as sum of the interactions that were increased in each comparison pair with subtraction of those that were decreased in each comparison pair. Where there are multiple ligands for a single receptor interaction count is calculated as the sum of the interaction count estimate of all ligands.

1 $$N={\sum }_{i=1}^{k}{n}_{l}^{{up}}-{n}_{l}^{{dn}}$$

### Trajectory analysis

RNA velocity analysis was performed by scVelo with dynamic mode. The calculated RNA velocity vectors were embedded to low-dimension space using the Partition-based graph abstraction (PAGA) module in scVelo package. Latent time of macrophage development was assigned to each sub-cluster of macrophages by scVelo with lower latent time indicating initial events and higher latent time indicating terminal events.

### Survival analysis by online webtool Kaplan-Meier Plotter

Kaplan Meier Plots for triple negative breast patient bulk RNA sequence database was generated by the online webtool Kaplan-Meier Plotter with restriction to only “basal” subtype. Survival analysis was assessed with univariate Cox regression with auto-select best cutoff provided by the Kaplan-Meier Plotter. For the survival analysis of C5aR1 high and low subgroups, patients were first stratified by median expression of C5aR1 and then the survival analysis was done with univariate Cox regression with auto-select best cutoff in aforementioned sub-groups individually. Survival analysis of GSE169246 was done by Graphpad Prism with univariate Cox regression.

### Survival analysis of TCGA basal breast cancer cohort

Basal breast cancer data of TCGA pan-cancer gene expression data was downloaded from UCSC Xena (http://xena.ucsc.edu/). Cell signature scores of each sub-cluster were calculated by UCell (v2.2) with top 3 DEGs of each cluster. Cell signature score ranks were calculated by Z-scores across each cell sub-cluster within the same sample. Cut-off of each score rank was set by cutpoint function of Survival R package. The hazard ratios (HRs) and log-rank test were performed using the Survival R package (v3.3-1).

### Pseudobulk correlation analysis

In order to visualize the correlation of macrophage specific gene and pathway co-expression pattern in all 40 MDST tumors, the co-expression correlation analysis was calculated by taking the average expression of indicated genes or pathway scores of the macrophages including each sub-cluster of macrophages for each sample and computed the Pearson correlation within the dataset containing all 40 MDST tumors. Correlations with r higher than 0.3 and *p*-value less than 0.05 were regarded as significant.

### Public data analysis

Processed human data of TNBC treated with ICB with or without chemotherapy was obtained from EGAD00001006608 and GSE169246. Cell annotation was based on expression of C5aR1 (C5aR1^hi^) and on expression of CD86 (CD86^hi^) and “other” that did not express high levels of CD86 or C5aR1. The upper quartile of C5aR1^hi^/CD86^hi^ ratio of the cohort was used as a cutoff for patient grouping. Survival analysis was assessed by univariate Cox regression using Graphpad Prism.

### Software used in this study

R (https://www.r-project.org/; RRID: SCR_001905, v4.1.0), SCANPY (https://scanpy.readthedocs.io/en/stable/, v1.7.1), scVelo (https://scvelo.readthedocs.io/en/stable/, v0.2.3), densVis (https://github.com/hhcho/densvis/tree/master/densmap), Cell Ranger (https://www.10xgenomics.com/support/software/cell-ranger/latest, v6.0.0), GSVA (https://github.com/rcastelo/GSVA, 1.40.1), Survival R (https://cran.r-project.org/web/packages/survival/survival.pdf, v3.3-1), samtools (https://github.com/samtools/samtools, v1.11), CellChat (https://github.com/sqjin/CellChat, v1.1.3), CellphoneDB (https://www.cellphonedb.org/), pySCENIC (https://github.com/aertslab/pySCENIC, v0.11.2), Seurat (https://satijalab.org/seurat/, 4.3.0.1), UCell (https://github.com/carmonalab/UCell, v2.2), scGPT (https://github.com/bowang-lab/scGPT v0.2.1), ImageJ (v1.52a), Prism (http://www.graphpad.com/faq/viewfaq.cfm?faq=1362, v8.0.2), FlowJo (https://www.flowjo.com/, v10). All statistical analysis was performed by GraphPad Prism software. Non-normally distributed data was assessed using the Mann-Whitney test. Wilcoxon matched-pairs signed rank test was used for paired data. Comparison among multiple groups was done by one-way ANOVA.

### Reporting summary

Further information on research design is available in the Nature Portfolio Reporting Summary linked to this article.

### Supplementary information

Supplementary information

Peer Review File

Description of Additional Supplementary Files

Supplementary Data 1

Reporting Summary

### Source data

Source Data

## Data Availability

The mouse-derived syngeneic transplant sequencing data generated in this study have been deposited in the GEO database under accession code GSE215908. The data of cancer cell lines used in this study are available in the GEO database under accession code GSE157220 [https://www.ncbi.nlm.nih.gov/geo/query/acc.cgi?acc=GSE15722]. The public human data of TNBC used in this study are available in the European Genome-phenome Archive (EGA) under accession code EGAD00001006608 [https://ega-archive.org/studies/EGAS00001004809] and in the GEO database under accession code GSE169246. Public RNA-seq dataset for breast cancer in the public database The Cancer Genome Atlas (TCGA, National Cancer Institute (NCI), Bethesda, MD, USA) were downloaded from the GDC portal (https://www.cbioportal.org/study/summary?id=brca_tcga_pub) The remaining data are available within the Article, Supplementary Information or Source Data file. Source data are provided with this paper.

## Code availability

This study did not generate custom code, and the wrapped code used in the study is freely available from https://github.com/xili3367/Left-and-right-co-transplantation (version: 01/21/21).

## References

[1] Litton JK (2018). Talazoparib in patients with advanced breast cancer and a germline BRCA mutation. N. Engl. J. Med..

[2] Robson M (2017). Olaparib for metastatic breast cancer in patients with a germline BRCA mutation. N. Engl. J. Med..

[3] Sun, C. et al. Rational combination therapy with PARP and MEK inhibitors capitalizes on therapeutic liabilities in RAS mutant cancers. *Sci. Transl. Med.* **9**, eaal5148 (2017). 10.1126/scitranslmed.aal5148 PMC5919217 28566428

[4] Lord CJ, Ashworth A (2013). Mechanisms of resistance to therapies targeting BRCA-mutant cancers. Nat. Med..

[5] Dev H (2018). Shieldin complex promotes DNA end-joining and counters homologous recombination in BRCA1-null cells. Nat. Cell Biol..

[6] Sun C, Fang Y, Labrie M, Li X, Mills GB (2020). Systems approach to rational combination therapy: PARP inhibitors. Biochem. Soc. Trans..

[7] Bhin J (2023). Multi-omics analysis reveals distinct non-reversion mechanisms of PARPi resistance in BRCA1- versus BRCA2-deficient mammary tumors. Cell Rep..

[8] Baxter JS, Zatreanu D, Pettitt SJ, Lord CJ (2022). Resistance to DNA repair inhibitors in cancer. Mol. Oncol..

[9] Labrie M (2021). Multiomics analysis of serial PARP inhibitor treated metastatic TNBC inform on rational combination therapies. NPJ Precis. Oncol..

[10] Chen Z, Wang X, Li X, Zhou Y, Chen K (2021). Deep exploration of PARP inhibitors in breast cancer: monotherapy and combination therapy. J. Int. Med. Res..

[11] Ledermann J (2014). Olaparib maintenance therapy in patients with platinum-sensitive relapsed serous ovarian cancer: a preplanned retrospective analysis of outcomes by BRCA status in a randomised phase 2 trial. Lancet Oncol..

[12] Poveda A (2022). Olaparib maintenance monotherapy in platinum-sensitive relapsed ovarian cancer patients without a germline BRCA1/BRCA2 mutation: OPINION primary analysis. Gynecol. Oncol..

[13] Shao, F. et al. Efficacy and safety of PARP inhibitors as the maintenance therapy in ovarian cancer: a meta-analysis of nine randomized controlled trials. *Biosci. Rep.* **40**, BSR20192226 (2020). 10.1042/BSR20192226 PMC7080644 32096544

[14] Gonzalez-Martin A (2023). Progression-free survival and safety at 3.5years of follow-up: results from the randomised phase 3 PRIMA/ENGOT-OV26/GOG-3012 trial of niraparib maintenance treatment in patients with newly diagnosed ovarian cancer. Eur. J. Cancer.

[15] Farkkila A (2020). Immunogenomic profiling determines responses to combined PARP and PD-1 inhibition in ovarian cancer. Nat. Commun..

[16] Harter, P. et al. Durvalumab with paclitaxel/carboplatin (PC) and bevacizumab (bev), followed by maintenance durvalumab, bev, and olaparib in patients (pts) with newly diagnosed advanced ovarian cancer (AOC) without a tumor BRCA1/2 mutation (non-tBRCAm): Results from the randomized, placebo (pbo)-controlled phase III DUO-O trial. *J. Clin. Oncol.* LBA5506 (2023).

[17] Banerjee, S. et al. Phase II study of olaparib plus durvalumab with or without bevacizumab (MEDIOLA): Final analysis of overall survival in patients with non-germline BRCA-mutated platinum-sensitive relapsed ovarian cancer. *Annals of Oncology*. **33**, S788–S789 (2022).

[18] Lampert EJ (2020). Combination of PARP inhibitor olaparib, and PD-L1 inhibitor durvalumab, in recurrent ovarian cancer: a proof-of-concept phase II study. Clin. Cancer Res..

[19] Konstantinopoulos PA (2019). Single-arm phases 1 and 2 trial of niraparib in combination with pembrolizumab in patients with recurrent platinum-resistant ovarian carcinoma. JAMA Oncol..

[20] Kaufman B (2015). Olaparib monotherapy in patients with advanced cancer and a germline BRCA1/2 mutation. J. Clin. Oncol..

[21] Tutt A (2010). Oral poly(ADP-ribose) polymerase inhibitor olaparib in patients with BRCA1 or BRCA2 mutations and advanced breast cancer: a proof-of-concept trial. Lancet.

[22] Amit I, Winter DR, Jung S (2016). The role of the local environment and epigenetics in shaping macrophage identity and their effect on tissue homeostasis. Nat. Immunol..

[23] Ma, R. Y., Black, A. & Qian, B. Z. Macrophage diversity in cancer revisited in the era of single-cell omics. *Trends Immunol*. **43**, 546–563 (2022). 10.1016/j.it.2022.04.008 35690521

[24] Anderson NR, Minutolo NG, Gill S, Klichinsky M (2021). Macrophage-based approaches for cancer immunotherapy. Cancer Res..

[25] Qian J (2020). A pan-cancer blueprint of the heterogeneous tumor microenvironment revealed by single-cell profiling. Cell Res..

[26] Cheng S (2021). A pan-cancer single-cell transcriptional atlas of tumor infiltrating myeloid cells. Cell.

[27] Yang B (2021). MEK inhibition remodels the immune landscape of mutant KRAS tumors to overcome resistance to PARP and immune checkpoint inhibitors. Cancer Res..

[28] Shen J (2015). ARID1A deficiency impairs the DNA damage checkpoint and sensitizes cells to PARP inhibitors. Cancer Discov..

[29] Westin SN (2021). Phase Ib dose expansion and translational analyses of olaparib in combination with capivasertib in recurrent endometrial, triple-negative breast, and ovarian cancer. Clin. Cancer Res..

[30] Mitri, Z. I. et al. Biomarker-driven selection of polyADP ribose polymerase inhibitors (PARPi)-based combination therapies in patients with metastatic triple negative breast cancer (mTNBC) [abstract]. *Proceedings of the American Association for Cancer Research Annual Meeting.* **82**, Abstract no. 2149 (2022).

[31] Mehta AK (2021). Targeting immunosuppressive macrophages overcomes PARP inhibitor resistance in BRCA1-associated triple-negative breast cancer. Nat. Cancer.

[32] Wang Q (2022). STING agonism reprograms tumor-associated macrophages and overcomes resistance to PARP inhibition in BRCA1-deficient models of breast cancer. Nat. Commun..

[33] Takahashi N, Surolia I, Thomas A (2020). Targeting DNA repair to drive immune responses: it’s time to reconsider the strategy for clinical translation. Clin. Cancer Res..

[34] Federico L (2017). A murine preclinical syngeneic transplantation model for breast cancer precision medicine. Sci. Adv..

[35] Wang R (2023). Evolution of immune and stromal cell states and ecotypes during gastric adenocarcinoma progression. Cancer Cell.

[36] Serin Harmanci A, Harmanci AO, Zhou X (2020). CaSpER identifies and visualizes CNV events by integrative analysis of single-cell or bulk RNA-sequencing data. Nat. Commun..

[37] Cords L (2023). Cancer-associated fibroblast classification in single-cell and spatial proteomics data. Nat. Commun..

[38] Murray PJ (2014). Macrophage activation and polarization: nomenclature and experimental guidelines. Immunity.

[39] Tietscher S (2023). A comprehensive single-cell map of T cell exhaustion-associated immune environments in human breast cancer. Nat. Commun..

[40] Zou Y (2023). The single-cell landscape of intratumoral heterogeneity and the immunosuppressive microenvironment in liver and brain metastases of breast cancer. Adv. Sci. (Weinh.).

[41] ProfileEkta Dadlani, P. D., Debashis Sahoo. An AI-assisted investigation of tumor-associated macrophages and their polarization in colorectal cancer. *BioRxiv*10.1101/2023.08.01.551559 (2023).

[42] Dos Anjos Cassado A (2017). F4/80 as a major macrophage marker: the case of the peritoneum and spleen. Results Probl. Cell Differ..

[43] Twum, D. Y. et al. IFN regulatory factor-8 expression in macrophages governs an antimetastatic program. *JCI Insight* **4**, 124267 (2019). 10.1172/jci.insight.124267 PMC6413790 30728331

[44] Zhang Y (2021). Single-cell analyses reveal key immune cell subsets associated with response to PD-L1 blockade in triple-negative breast cancer. Cancer Cell.

[45] Cui, H. et al. scGPT: toward building a foundation model for single-cell multi-omics using generative AI. *Nat. Methods*10.1038/s41592-024-02201-0 (2024). 10.1038/s41592-024-02201-0 38409223

[46] Bassez A (2021). A single-cell map of intratumoral changes during anti-PD1 treatment of patients with breast cancer. Nat. Med..

[47] Liu C (2023). Single-cell RNA-sequencing reveals radiochemotherapy-induced innate immune activation and MHC-II upregulation in cervical cancer. Signal Transduct. Target Ther..

[48] Kilian M (2023). MHC class II-restricted antigen presentation is required to prevent dysfunction of cytotoxic T cells by blood-borne myeloids in brain tumors. Cancer Cell.

[49] Li, Z. Y. et al. The transcriptional repressor ID2 supports natural killer cell maturation by controlling TCF1 amplitude. *J. Exp. Med.* **218**, e20202032 (2021). 10.1084/jem.20202032 PMC8056751 33857289

[50] Jeong S, Park SH (2020). Co-stimulatory receptors in cancers and their implications for cancer immunotherapy. Immune Netw..

[51] Karasu E (2020). Complement C5a induces pro-inflammatory microvesicle shedding in severely injured patients. Front Immunol..

[52] Illuzzi G (2022). Preclinical characterization of AZD5305, a next-generation, highly selective PARP1 inhibitor and trapper. Clin. Cancer Res..

[53] Iyer S (2021). Genetically defined syngeneic mouse models of ovarian cancer as tools for the discovery of combination immunotherapy. Cancer Discov..

[54] Bergen V, Lange M, Peidli S, Wolf FA, Theis FJ (2020). Generalizing RNA velocity to transient cell states through dynamical modeling. Nat. Biotechnol..

[55] Narayan A, Berger B, Cho H (2021). Assessing single-cell transcriptomic variability through density-preserving data visualization. Nat. Biotechnol..

[56] Otsuka R, Wada H, Seino KI (2021). IL-34, the rationale for its expression in physiological and pathological conditions. Semin Immunol..

[57] Ricketts TD, Prieto-Dominguez N, Gowda PS, Ubil E (2021). Mechanisms of macrophage plasticity in the tumor environment: manipulating activation state to improve outcomes. Front Immunol..

[58] Franklin RA, Li MO (2016). Ontogeny of tumor-associated macrophages and its implication in cancer regulation. Trends Cancer.

[59] Biswas SK, Mantovani A (2010). Macrophage plasticity and interaction with lymphocyte subsets: cancer as a paradigm. Nat. Immunol..

[60] Cassetta L (2019). Human tumor-associated macrophage and monocyte transcriptional landscapes reveal cancer-specific reprogramming, biomarkers, and therapeutic targets. Cancer Cell.

[61] Zaidan, I., et al. Angiotensin-(1-7)/MasR axis promotes migration of monocytes/macrophages with a regulatory phenotype to perform phagocytosis and efferocytosis. *JCI Insight* **7**, e147819 (2022). 10.1172/jci.insight.147819 PMC8765051 34874920

[62] Zhang C, Yang M, Ericsson AC (2021). Function of macrophages in disease: current understanding on molecular mechanisms. Front Immunol..

[63] Aibar S (2017). SCENIC: single-cell regulatory network inference and clustering. Nat. Methods.

[64] Zhao X (2022). MEF2C promotes M1 macrophage polarization and Th1 responses. Cell Mol. Immunol..

[65] Oishi Y (2017). SREBP1 contributes to resolution of pro-inflammatory TLR4 signaling by reprogramming fatty acid metabolism. Cell Metab..

[66] Boonyatecha N, Sangphech N, Wongchana W, Kueanjinda P, Palaga T (2012). Involvement of Notch signaling pathway in regulating IL-12 expression via c-Rel in activated macrophages. Mol. Immunol..

[67] Kovarik P, Stoiber D, Novy M, Decker T (1998). Stat1 combines signals derived from IFN-gamma and LPS receptors during macrophage activation. EMBO J..

[68] Liu, T., Zhang, L., Joo, D. & Sun, S. C. NF-kappaB signaling in inflammation. *Signal Transduct Target Ther.* **2**, 17023 (2017). 10.1038/sigtrans.2017.23 PMC5661633 29158945

[69] Xie C (2016). Effects of IRF1 and IFN-beta interaction on the M1 polarization of macrophages and its antitumor function. Int J. Mol. Med..

[70] Mahabeleshwar GH (2011). The myeloid transcription factor KLF2 regulates the host response to polymicrobial infection and endotoxic shock. Immunity.

[71] Ruffell D (2009). A CREB-C/EBPbeta cascade induces M2 macrophage-specific gene expression and promotes muscle injury repair. Proc. Natl Acad. Sci. USA.

[72] Liu M (2020). Transcription factor c-Maf is a checkpoint that programs macrophages in lung cancer. J. Clin. Invest.

[73] Mohapatra S, Pioppini C, Ozpolat B, Calin GA (2021). Non-coding RNAs regulation of macrophage polarization in cancer. Mol. Cancer.

[74] Alam Z (2020). Counter regulation of spic by NF-kappaB and STAT signaling controls inflammation and iron metabolism in macrophages. Cell Rep..

[75] Yu Z (2015). MSX3 switches microglia polarization and protects from inflammation-induced demyelination. J. Neurosci..

[76] Castro-Mondragon JA (2022). JASPAR 2022: the 9th release of the open-access database of transcription factor binding profiles. Nucleic Acids Res..

[77] Gyorffy B (2021). Survival analysis across the entire transcriptome identifies biomarkers with the highest prognostic power in breast cancer. Comput. Struct. Biotechnol. J..

[78] Jiang, S. & Sun, L. Tongue Sole CD209: a pattern-recognition receptor that binds a broad range of microbes and promotes phagocytosis. *Int. J. Mol. Sci.* **18**, 1848 (2017). 10.3390/ijms18091848 PMC5618497 28869534

[79] Cullen SP, Brunet M, Martin SJ (2010). Granzymes in cancer and immunity. Cell Death Differ..

[80] Hargadon KM, Johnson CE, Williams CJ (2018). Immune checkpoint blockade therapy for cancer: An overview of FDA-approved immune checkpoint inhibitors. Int Immunopharmacol..

[81] Dirix LY (2018). Avelumab, an anti-PD-L1 antibody, in patients with locally advanced or metastatic breast cancer: a phase 1b JAVELIN Solid Tumor study. Breast Cancer Res Treat..

[82] Pusztai L (2021). Durvalumab with olaparib and paclitaxel for high-risk HER2-negative stage II/III breast cancer: Results from the adaptively randomized I-SPY2 trial. Cancer Cell.

[83] Stringer-Reasor EM (2021). An open-label, pilot study of veliparib and lapatinib in patients with metastatic, triple-negative breast cancer. Breast Cancer Res..

[84] Cheng H, Wang Z, Fu L, Xu T (2019). Macrophage polarization in the development and progression of ovarian cancers: an overview. Front Oncol..

[85] Yan S, Wan G (2021). Tumor-associated macrophages in immunotherapy. FEBS J..

[86] Gomez-Roca CA (2019). Phase I study of emactuzumab single agent or in combination with paclitaxel in patients with advanced/metastatic solid tumors reveals depletion of immunosuppressive M2-like macrophages. Ann. Oncol..

[87] Cannarile MA (2017). Colony-stimulating factor 1 receptor (CSF1R) inhibitors in cancer therapy. J. Immunother. Cancer.

[88] Papadopoulos KP (2017). First-in-human study of AMG 820, a monoclonal anti-colony-stimulating factor 1 receptor antibody, in patients with advanced solid tumors. Clin. Cancer Res..

[89] Wesolowski R (2019). Phase Ib study of the combination of pexidartinib (PLX3397), a CSF-1R inhibitor, and paclitaxel in patients with advanced solid tumors. Ther. Adv. Med. Oncol..

[90] von Tresckow B (2015). An open-label, multicenter, phase I/II study of JNJ-40346527, a CSF-1R inhibitor, in patients with relapsed or refractory Hodgkin lymphoma. Clin. Cancer Res.

[91] Tap WD (2015). Structure-Guided Blockade of CSF1R Kinase in Tenosynovial Giant-Cell Tumor. N. Engl. J. Med.

[92] Wang Y, Zhang H, He YW (2019). The complement receptors C3aR and C5aR are a new class of immune checkpoint receptor in cancer immunotherapy. Front Immunol..

[93] Medler TR (2018). Complement C5a fosters squamous carcinogenesis and limits T cell response to chemotherapy. Cancer Cell.

[94] Piao C (2018). Complement 5a stimulates macrophage polarization and contributes to tumor metastases of colon cancer. Exp. Cell Res..

[95] Markiewski MM (2008). Modulation of the antitumor immune response by complement. Nat. Immunol..

[96] Markiewski MM (2017). The ribosomal protein S19 suppresses antitumor immune responses via the complement C5a Receptor 1. J. Immunol..

[97] Ou B (2021). C5aR1-positive neutrophils promote breast cancer glycolysis through WTAP-dependent m6A methylation of ENO1. Cell Death Dis..

[98] Bourgeois CT, Satou R, Prieto MC (2017). HDAC9 is an epigenetic repressor of kidney angiotensinogen establishing a sex difference. Biol. Sex. Differ..

[99] Ding L (2018). PARP inhibition elicits STING-dependent antitumor immunity in Brca1-deficient ovarian cancer. Cell Rep..

[100] Pantelidou C (2019). PARP inhibitor efficacy depends on CD8(+) T-cell recruitment via intratumoral STING pathway activation in BRCA-deficient models of triple-negative breast cancer. Cancer Discov..

[101] Shen J (2019). PARPi triggers the STING-dependent immune response and enhances the therapeutic efficacy of immune checkpoint blockade independent of BRCAness. Cancer Res.

[102] Wang Z (2019). Niraparib activates interferon signaling and potentiates anti-PD-1 antibody efficacy in tumor models. Sci. Rep..

[103] Toda G, Yamauchi T, Kadowaki T, Ueki K (2021). Preparation and culture of bone marrow-derived macrophages from mice for functional analysis. STAR Protoc..

[104] La Manno G (2018). RNA velocity of single cells. Nature.

[105] Wolf FA, Angerer P, Theis FJ (2018). SCANPY: large-scale single-cell gene expression data analysis. Genome Biol..

[106] Wolock SL, Lopez R, Klein AM (2019). Scrublet: computational identification of cell doublets in single-cell transcriptomic data. Cell Syst..

[107] Jin S (2021). Inference and analysis of cell-cell communication using CellChat. Nat. Commun..

[108] Vento-Tormo R (2018). Single-cell reconstruction of the early maternal-fetal interface in humans. Nature.

