# Supplementary material for: C5aR1 inhibition reprograms tumor associated macrophages and reverses PARP inhibitor resistance in breast cancer
Source: Nat Commun. 2024 May 27;15:4485. doi: 10.1038/s41467-024-48637-y (PMC11130309; doi:10.1038/s41467-024-48637-y)
Supplement: Supplementary file 1 — Supplementary information [file 41467_2024_48637_MOESM1_ESM.pdf]

C5aR1 inhibition reprograms tumor associated macrophages and reverses PARP inhibitor resistance in breast cancer

Supplementary Figure 1

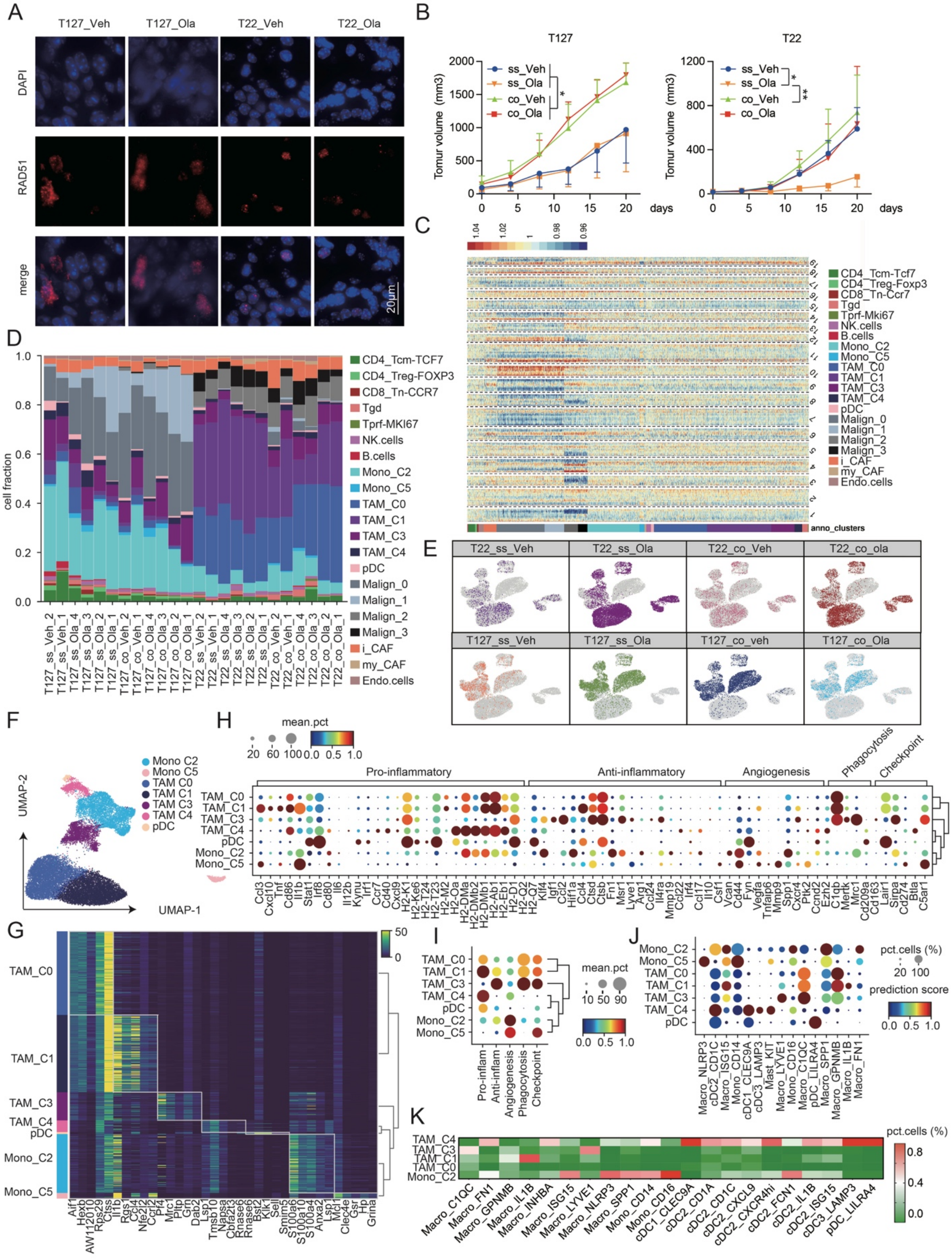

Supplementary Figure 1: scRNA-seq analysis of T22, T127 and co-transplanted tumors

(A) Representative immunofluorescence images of RAD51 foci formation in indicated MDST tumors. Veh: vehicle. Ola: olaparib. Data are representative of three replicates.
(B) Tumor growth curve of T127 single strain and co-transplanted tumors treated with olaparib for 20 days. Tumor size of single strain transplantation models were calculated as average size of both sides. ss: single strain transplantation models. co: co-transplantation models. T22_co: T22 site of co-transplantation model. Veh: vehicle. Ola: olaparib. Sample numbers are indicated in Figure 1B and C. All data are presented as mean values +/- SD. *p* values are from one-way ANOVA. * $p < 0.05$, ** $P < 0.01$, *** $p < 0.001$, **** $p < 0.0001$. Source data and exact *p* values are provided as a Source Data file.
(C) CNV analysis of T22 and T127 tumors by CaSpER.
(D) Cell fraction of different cell in T22 and T127 tumors from indicated treatment determined by markers in Figure 1E (epithelial, fibroblast, endothelial, NK, T, Myeloid and DC cells).
(E) Distribution of cells in T127 and T22 tumors in the UMAP of Figure 1D. ss: single strain transplantation models. co: co-transplantation models. T127_co_Ola_1: 1089 cells, T127_co_Ola_2: 890 cells, T127_co_Ola_3: 1180 cells, T127_co_Ola_4: 1170 cells, T127_co_Veh_1: 508 cells, T127_co_Veh_2: 801 cells, T127_ss_Ola_1: 1639 cells, T127_ss_Ola_2: 1408 cells, T127_ss_Ola_3: 534 cells, T127_ss_Ola_4: 676 cells, T127_ss_Veh_1: 597 cells, T127_ss_Veh_2: 454 cells, T22_co_Ola_1: 1892 cells, T22_co_Ola_2: 989 cells, T22_co_Ola_3: 415 cells, T22_co_Ola_4: 613 cells, T22_co_Veh_1: 801 cells, T22_co_Veh_2: 856 cells, T22_ss_Ola_1: 2481 cells, T22_ss_Ola_2: 2108 cells, T22_ss_Ola_3: 1755 cells, T22_ss_Ola_4: 1665 cells, T22_ss_Veh_1: 435 cells, T22_ss_Veh_2: 709 cells. For vehicle treated, n = 2 and olaparib treated, n = 4.
(F) Distribution of each sub-cluster of myeloid cells in UMAP.
(G) Heatmap of top 20 differential expression genes of each sub-cluster of myeloid cells.
(H) Dot plot of markers representing each pathway of indicated TAM clusters.
(I) Dot plot of summarized expression level of each pathway of indicated TAM clusters. Gene lists used for functional pathway scores are listed in (H).
(J) Prediction scores of indicated macrophage clusters transferred from a pan-cancer infiltrating myeloid study to MDST datasets by label transfer.
(K) Cell fraction of annotated MDST myeloid clusters in each human myeloid cluster by scGPT. Source data are provided as a Source Data file.

Supplementary Figure 2

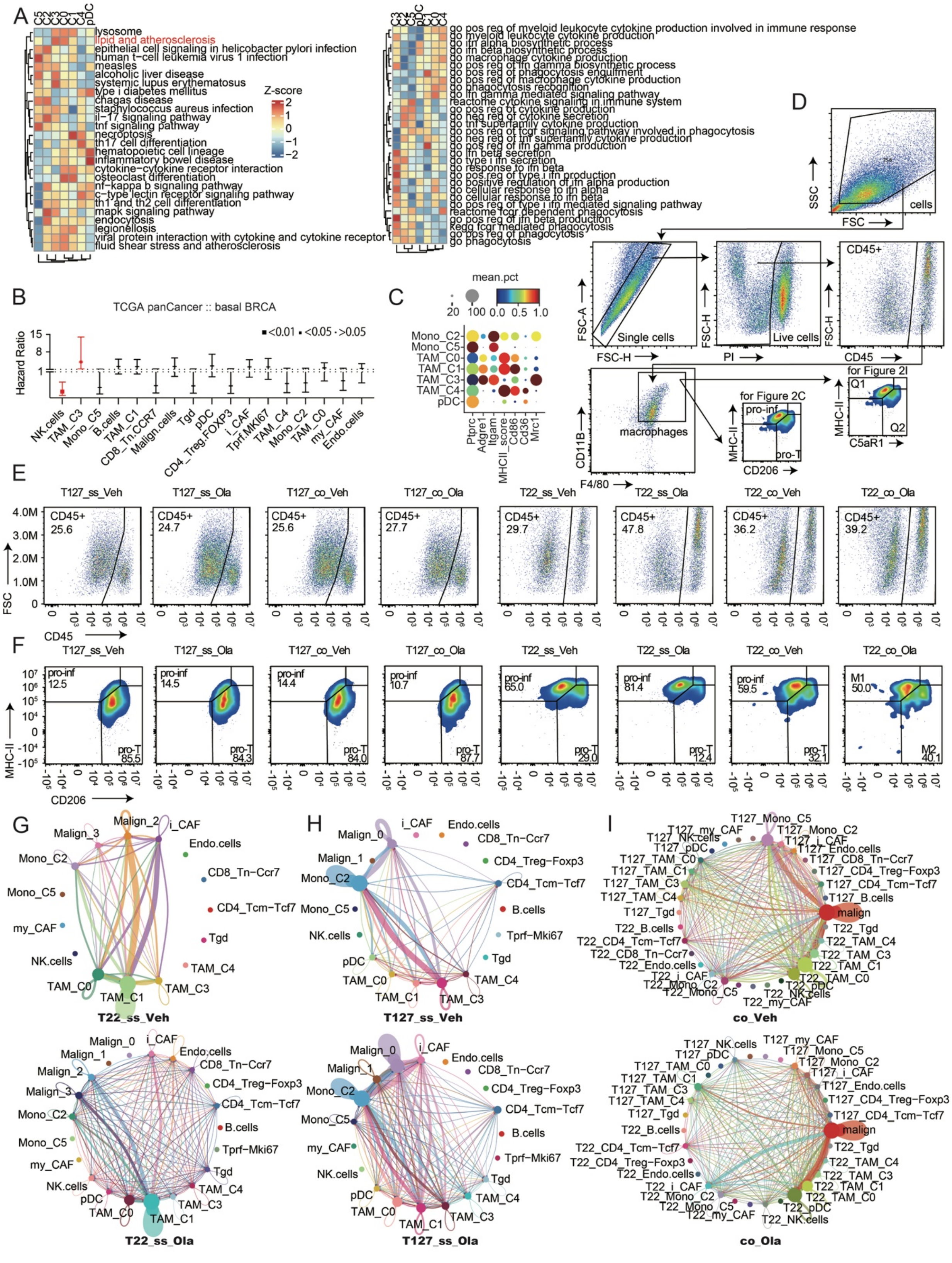

Supplementary Figure 2. Landscape of tumor microenvironment of T22 and T127 tumors with and without PARPi treatment

(A) Heatmap showing GSVA enrichment scores of differentially expressed pathways of indicated TAM clusters (left), and heatmap showing GSVA enrichment scores of immune related pathways of indicated TAM clusters (right).
(B) Dot chart of Hazard Ratio of indicated cell signature rank in a TCGA basal breast cancer database. Cutoff of rank of cell signature was set by surv_cutpoint function of package 'survival'. *p* values are shown by dot size. n=171.
(C) Dot plot of subtype related markers of indicated TAM clusters.
(D) Work flow of flow cytometry analysis of lymphocyte panel for Figure 2C and 2J. After gating cells as macrophages by staining with CD11B and F4/80, for Figure 2C macrophage subtypes were differentiated into MHC-II^{hi}CD206^{lo} or MHCII^{lo}CD206^{hi} by staining with MHCII and CD206, for Figure 2J macrophage subtypes were differentiated into MHC-II^{hi}C5aR1^{lo} or MHCII^{lo}C5aR1^{hi} by staining with MHCII and C5aR1.
(E) Distribution pattern of CD45^{+} cells in each indicated group. n=5.
(F) Distribution pattern of MHC-II^{hi}CD206^{lo} or MHCII^{lo}CD206^{hi} TAMs in each indicated group. n=5.
(G-I) Circle plot of cell-cell communication from CellChat among each component in each treatment group. ss: single strain models. ss: single strain transplantation models. co: co-transplantation models. Veh: vehicle. Ola: olaparib. Source data are provided as a Source Data file.

Supplementary Figure 3

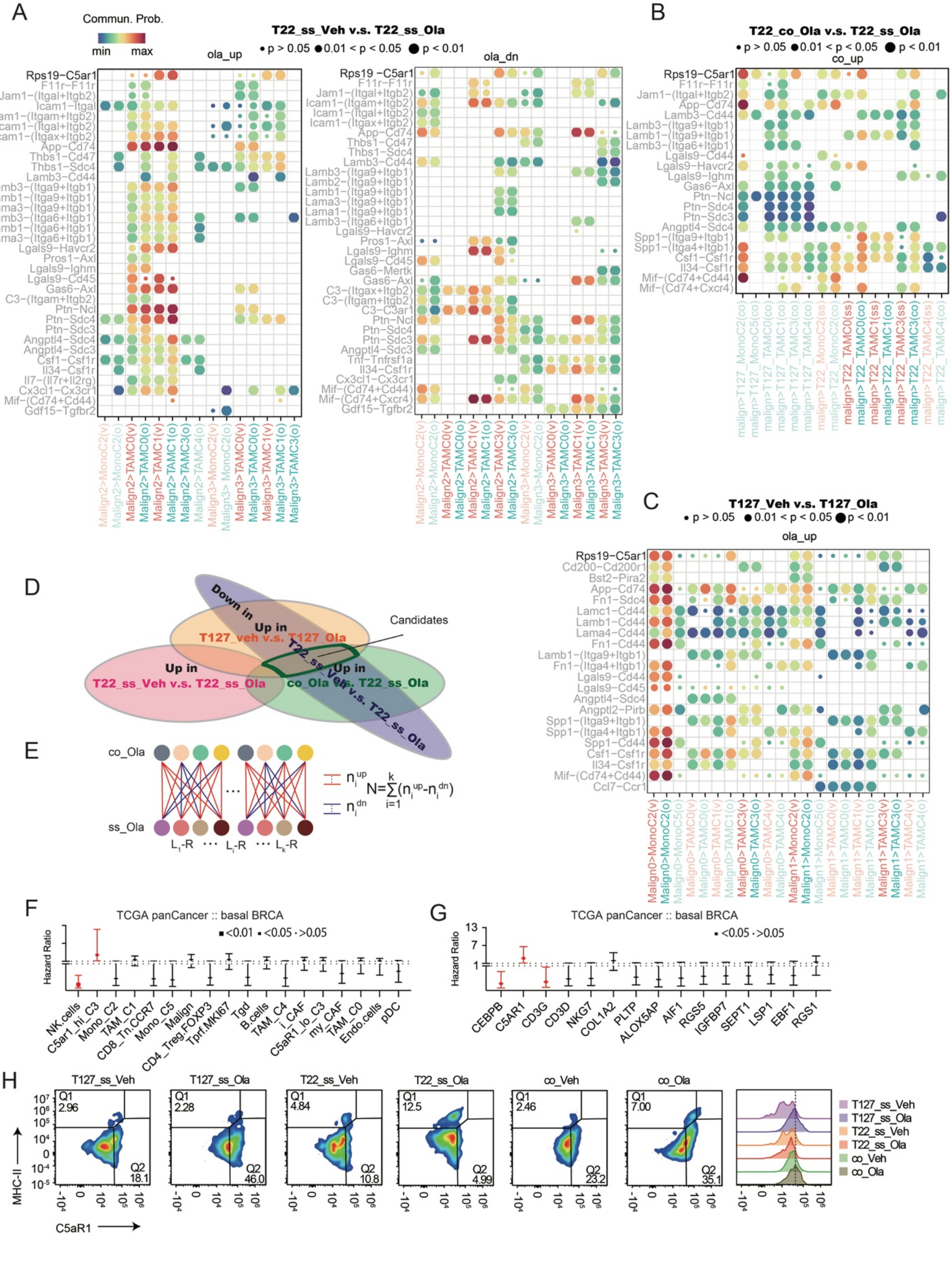

Supplementary Figure 3. Changes in ligand-receptor pair communication in T22 and T127 models

(A) Ligand-receptor pair changes in single strain transplanted T22 tumors with versus without olaparib treatment.
(B) Ligand-receptor pair changes in co-transplanted T22 tumors with olaparib treatment compared with single strain transplanted T22 tumors with olaparib treatment. ss: single strain transplantation models. co: co-transplantation models. Veh: vehicle. Ola: olaparib. Interactions of ligand-receptor pairs from Figure 1G are highlighted.
(C) Ligand-receptor pair changes in single strain transplanted T127 tumors with versus without olaparib treatment.
(D) Design of ligand-receptor candidate selection strategy. Candidate ligand-receptor pairs represent those that mediate communication between tumor cells and macrophages in T127 single strain and were upregulated by olaparib in T127 (resistant to olaparib), were not present or weakly present in T22 single strain (sensitive to olaparib) and were up-regulated in T22 in the co-transplantation model treated by olaparib (resistant to olaparib).
(E) Design of interaction count estimation based on CellChat outputs comparing olaparib treated single strain T22 tumors to T22 co-transplanted tumors. Interaction count estimate is the sum of interactions that were increased in each comparison pair using individual tumors and subtracted those that were decreased in each comparison pair (see Methods).
(F) Dot chart of Hazard Ratio of indicated cell signature rank in a TCGA basal breast cancer database. Cutoff of rank of cell signature were set by surv_cutpoint function of package 'survival'. *p* values are shown by dot size. n=171.
(G) Dot chart of Hazard Ratio of indicated gene in a TCGA basal breast cancer database. Cutoff of each gene was set by surv_cutpoint function of package 'survival'. *p* values are shown by dot size. n=171.
(H) Representative cell distribution pattern of macrophages in blood from T22, T127 and co-transplantation models with and without olaparib treatment. Source data are provided as a Source Data file.

Supplementary Figure 4

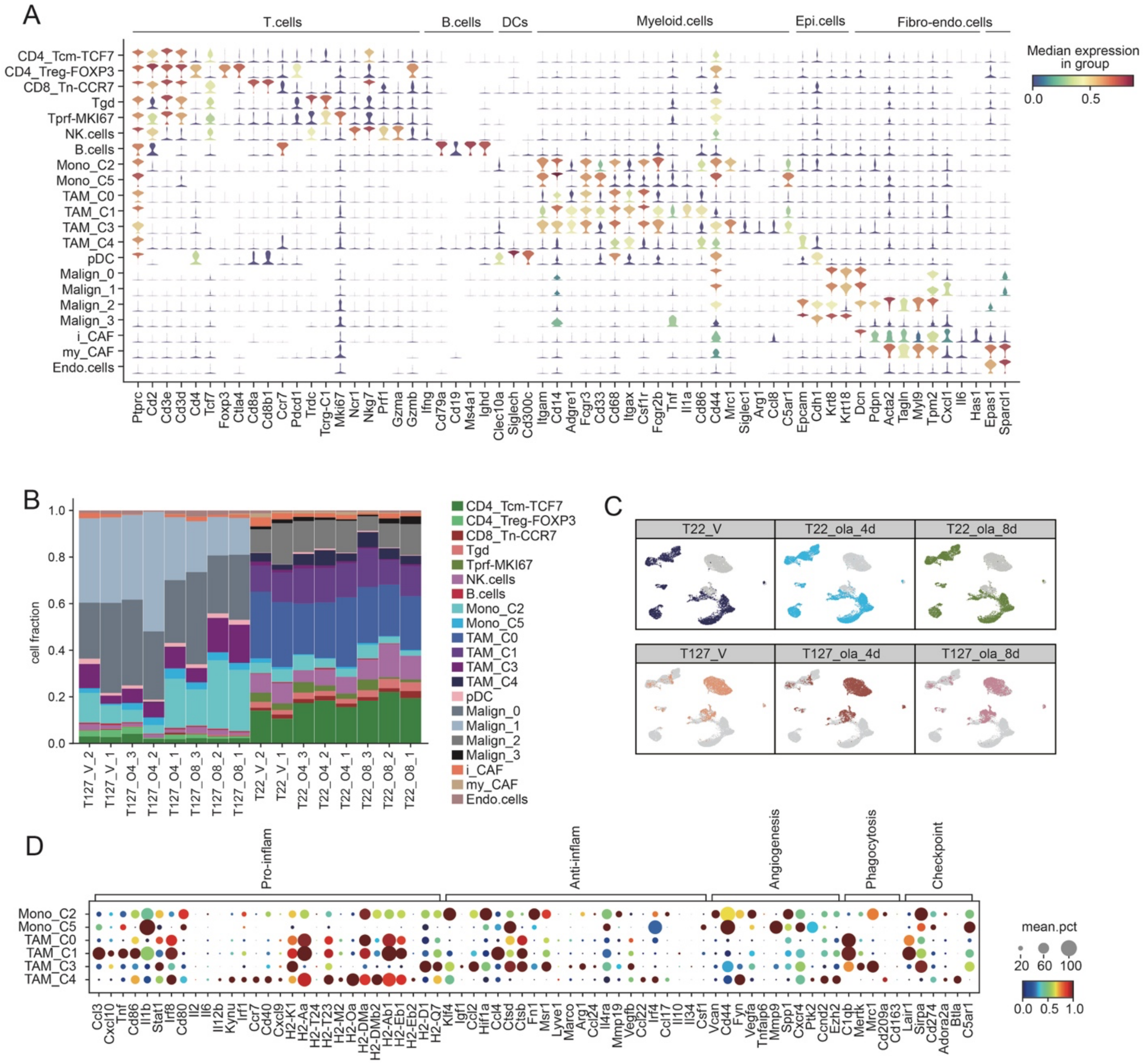

Supplementary Figure 4. Landscape of tumor microenvironment of T22 and T127 short-term olaparib treated tumors

(A) Violin plot of markers to verify annotation of T subtypes and macrophage subtypes and the markers used for subtyping epithelial, fibroblast, endothelial, dendritic and NK cells.

(B) Cell fraction of each cell type in T22 and T127 tumors V: vehicle, O4: olaparib for 4 days, O8: olaparib for 8 days.

(C) Distribution of T22 and T127 tumor clusters from individual mouse in the UMAP Figure 3D. V: vehicle, O4: olaparib for 4 days, O8: olaparib for 8 days. T22_O4_1: 2152 cells, T22_O4_2: 2482 cells, T22_O4_3: 1694 cells, T22_O8_1: 1701 cells, T22_O8_2: 1094 cells, T22_O8_3: 1807 cells, T22_V_1: 1436 cells, T22_V_2: 2114 cell,T127_O4_1: 1147 cells, T127_O4_2: 771 cells, T127_O4_3: 1136 cells, T127_O8_1: 1113 cells, T127_O8_2: 1203 cells, T127_O8_3: 832 cells, T127_V_1: 1180 cells, T127_V_2: 695 cells. For vehicle treated, n = 2 and olaparib treated, n = 3.

(D) Dot plot of markers representing each pathway of indicated TAM clusters.

Supplementary Figure 5

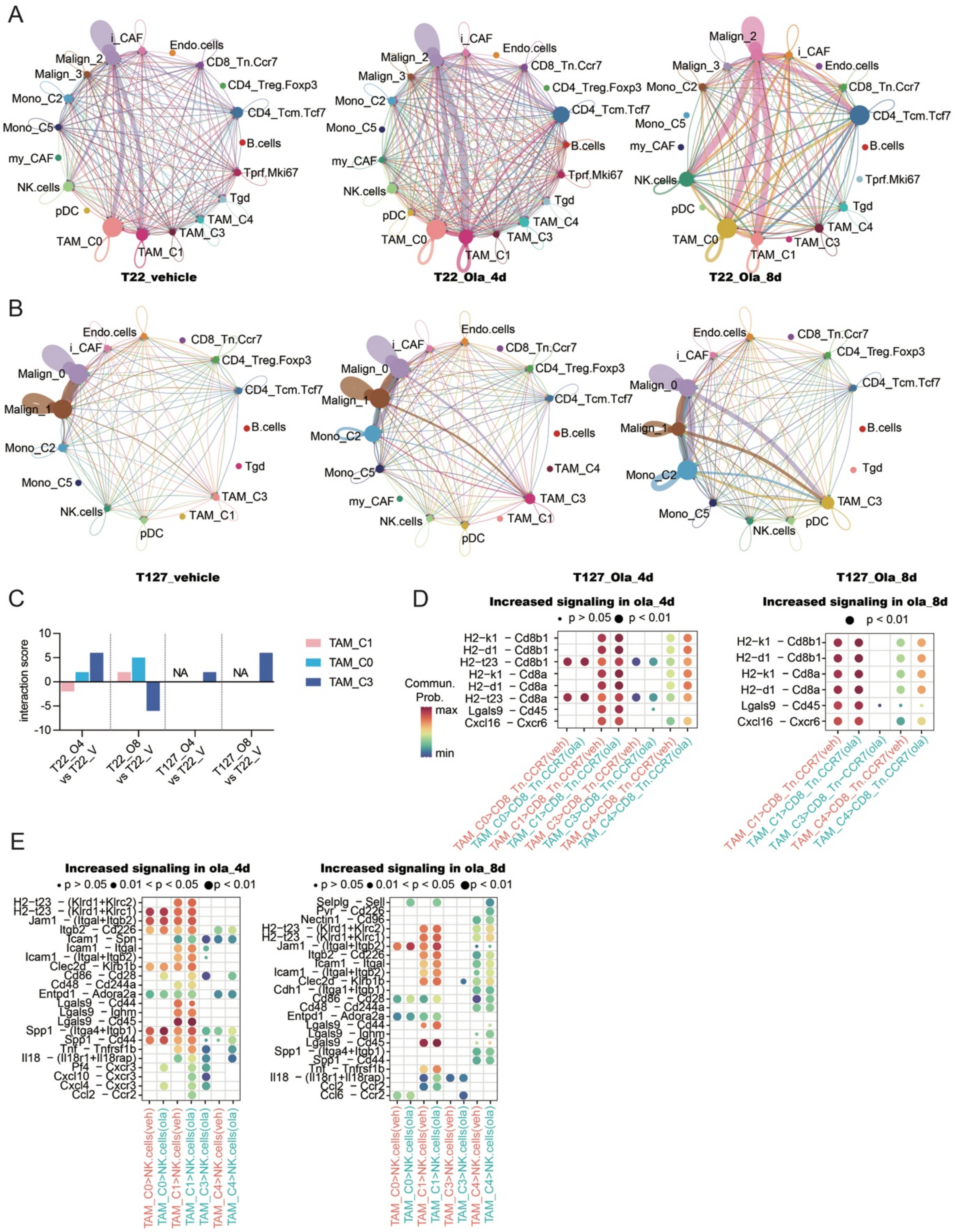

Supplementary Figure 5. CellChat predicted communication between TAMs and lymphocytes in T22 and T127 tumors

(A) Circle plot of cell-cell communication among each cell type in T22 tumors treated with vehicle or olaparib for 4 days or 8 days.

(B) Circle plot of cell-cell communication among each cell type in T127 tumors treated with vehicle or olaparib for 4 days or 8 days..

(C) Bar plot showing alternated ligand-receptor pair interaction count estimate between malignant cells and each TAM cluster in the T22 or T127 tumor after olaparib treatment compared to the T22 or T127 after vehicle treatment.

(D) Significant up-regulated ligand-receptor communications between macrophages and naïve CD8 T cells in T22 treated with vehicle or olaparib for 4 days or 8 days.

(E) Significant up-regulated ligand-receptor communications between macrophages and NK cell in T22 treated with vehicle or olaparib for 4 days or 8 days. Source data are provided as a Source Data file.

Supplementary Figure 6

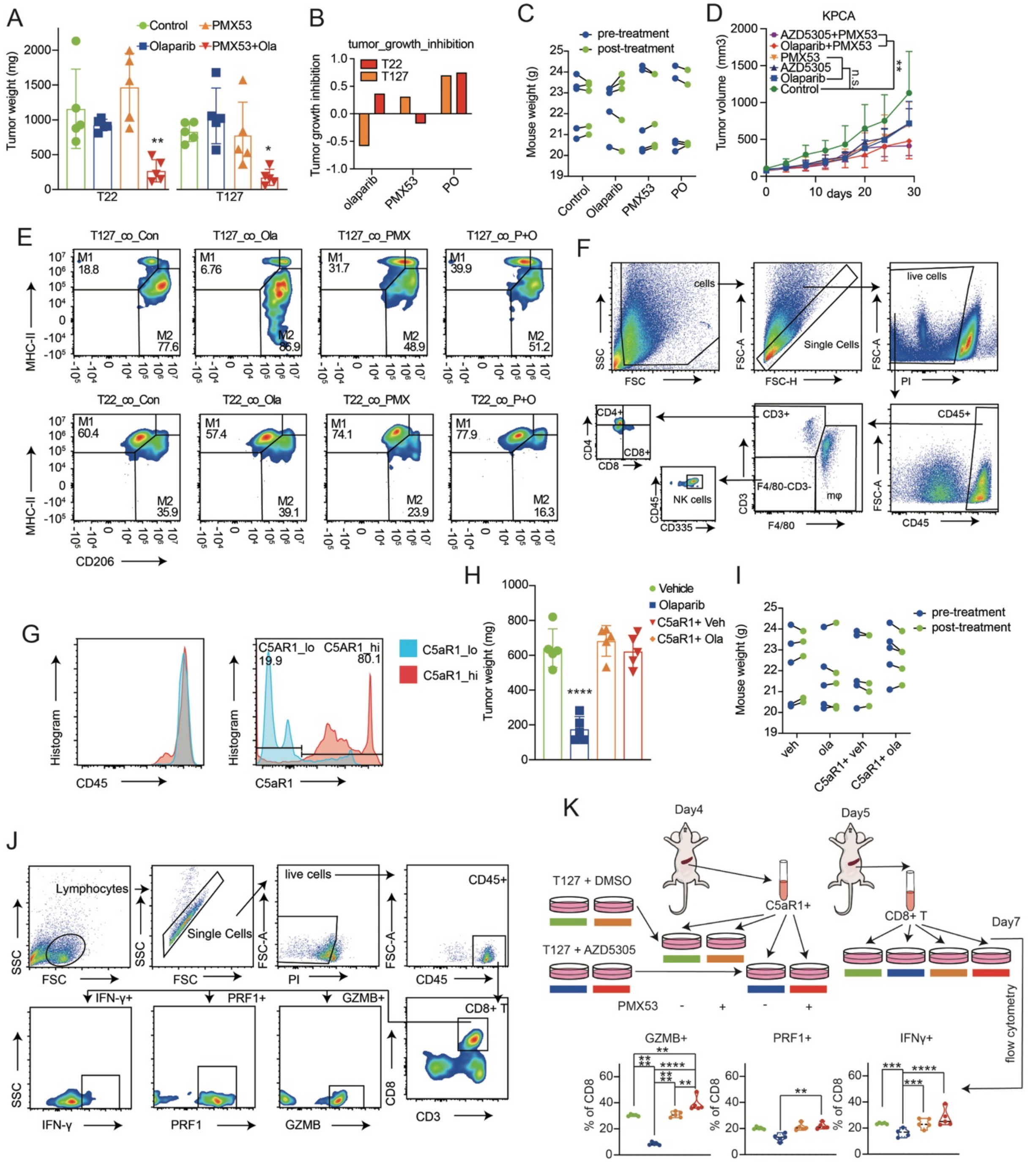

Supplementary Figure 6. Response and immune characterization of tumors treated with PMX-53 with and without olaparib

(A) Tumor weight of T22 (left) and T127 (right) of co-transplanted T22 and T127 tumor models treated with control (vehicle + PMX-53C), olaparib, PMX-53 or the combination for 14 days. n=5.

(B) Tumor growth inhibition calculated based on last day tumor volume. PO = PMX53+olaparib. n=5.

(C) Mouse weight changes pre- and post- treatment with control, olaparib, PMX-53 or the combination. n=5.

(D) Tumor growth curve of KPCA mouse model treated with control, olaparib, AZD5305, PMX53, the combination of PMX53 and olaparib or the combination of PMX53 and AZD5305 for 28 days. The day 29 volumes were derived

by measurement of dissected tumors which is more accurate that measurement of masses in the mice. For vehicle treated and AZD5305+PMX53 treated, n = 7 and the other groups, n = 8.
(E) Representative cell distribution pattern of $MHCII^{hi}CD206^{lo}$ and $MHCII^{lo}CD206^{hi}$ in each treatment for Figure 4C. n=5.
(F) Work flow of flow cytometry analysis of lymphocyte panel for Figure 4D. n=5.
(G) Purity of $C5aR1^{hi}$ cells assessed by flow cytometry after magnetic column selection.
(H) Tumor weight of T22 of mice receiving $C5aR1^{hi}$ cells treated with control, olaparib, PMX53 or the combination for 8 days. n=5.
(I) Mouse weight changes pre- and post-treatment of mice receiving $C5aR1^{hi}$ cells with and without olaparib. n=5.
(J) Work flow of flow cytometry analysis of CD8 T panel for Figure 5. n=3.
(K) Work flow of co-culture assay: T127 tumor cells were purified from mice and then treated with vehicle or AZD5305 (5nM) for 4 days in culture. $CD11b^{+}C5aR1^{-/+}$ macrophages isolated from tumor naive mice were added on day 4. 1×10^{6} T127 tumor cells were treated with or without AZD5305 (5nM) in culture for 4 days. They were co-cultured for 1 additional day with 5×10^{5} $CD11b^{+}C5aR1^{-/+}$ macrophages isolated from $CD11b^{+}$ positive splenocytes from tumor naïve mice with or without PMX53 (40nM). On day 5, $CD11b^{+}C5aR1^{-/+}$ macrophages were isolated and then co-cultured with 5×10^{5} CD8 T cells from tumor naïve mice. Fraction of indicated CD8 T cells in each indicated group assessed by flow cytometry. n=5, All data are presented as mean values +/- SD. *p* values are from one-way ANOVA. * $p < 0.05$, ** $P < 0.01$, *** $p < 0.001$, **** $p < 0.0001$. Source data and exact *p* values are provided as a Source Data file. Source data are provided as a Source Data file.

Supplementary Figure 7

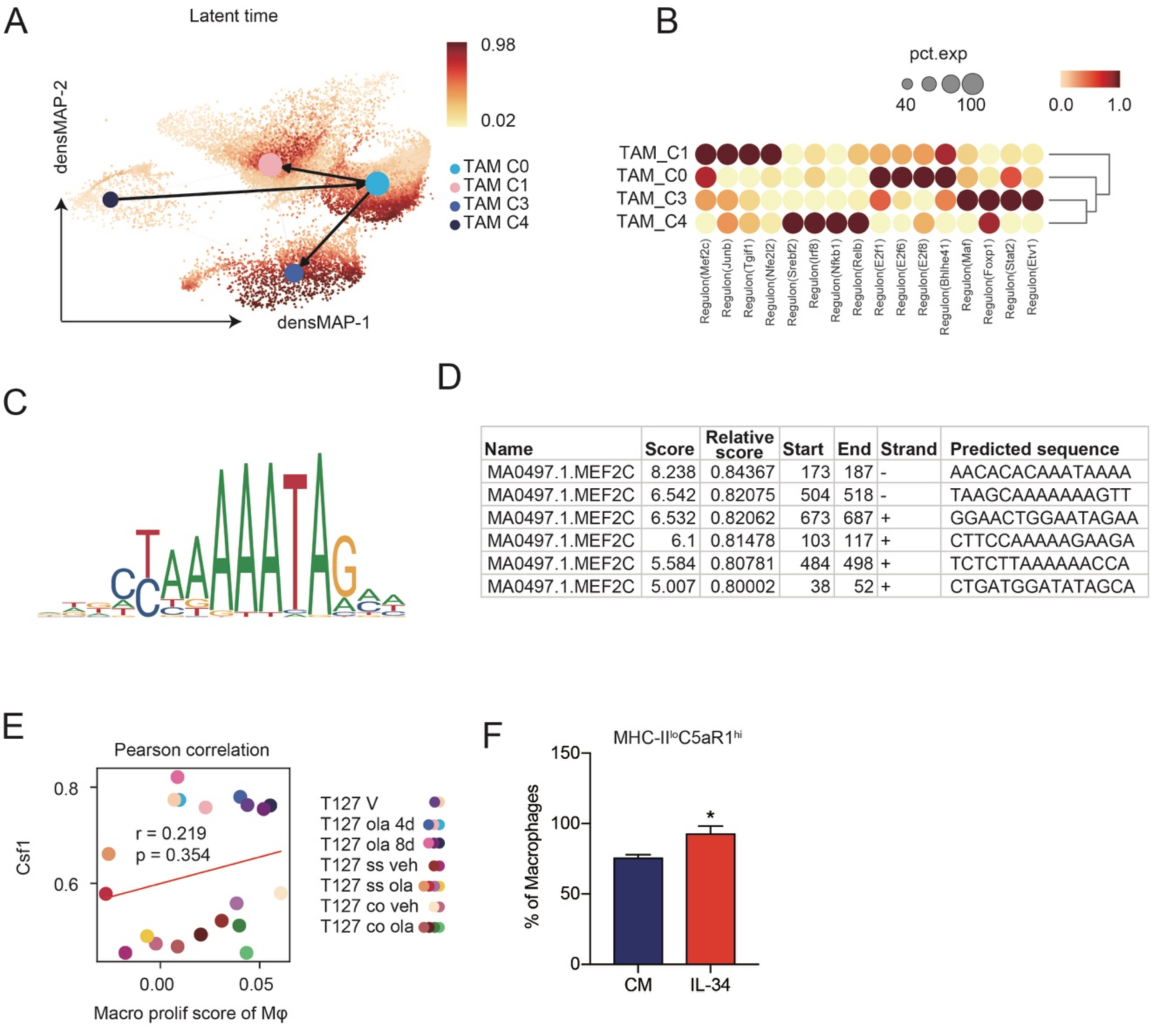

Name	Score	Relative score	Start	End	Strand	Predicted sequence
MA0497.1.MEF2C	8.238	0.84367	173	187	-	AACACACAAATAAAA
MA0497.1.MEF2C	6.542	0.82075	504	518	-	TAAGCAAAAAAAGTT
MA0497.1.MEF2C	6.532	0.82062	673	687	+	GGAACTGGAATAGAA
MA0497.1.MEF2C	6.1	0.81478	103	117	+	CTTCCAAAAAGAAGA
MA0497.1.MEF2C	5.584	0.80781	484	498	+	TCTCTTAAAAAACCA
MA0497.1.MEF2C	5.007	0.80002	38	52	+	CTGATGGATATAGCA

Supplementary Figure 7. Trajectory analysis of TAM_C3 polarization and regulation of C5aR1

(A) Partition-based graph abstraction (PAGA) representation of cell sub-clusters using latent time in scVelo were mapped on a density preserving uniform manifold approximation and projection map (densMAP) colored by latent time score of each sub-cluster of macrophages from all tumors mentioned in Figure 1 and Figure 3.

(B) Dotplot showing AUC of indicated transcription factors enriched by pySCENIC in each macrophage subtype of the pooled macrophages from all the tumors mentioned in (A).

(C) Jaspar predicted motif of Mef2c binding cite of C5ar1.

(D) Binding potential of Mef2c to C5ar1 transcription regulation region predicated by Jaspar.

(E) Correlation between Csf1 mRNA expressed by malignant cells versus the macrophage proliferative score from the same tumor of (A). V: tumors treated with vehicle for short term. veh: vehicle. ola: olaparib. *p* value was from Pearson analysis.

(F) Fraction of MHCII^{lo}C5aR1^{hi} macrophages of total macrophages of BMDM cells after treated with complete media with or without IL34. CM: complete medium. n=3. Comparison between groups was done by two-sided Student's *t* test. Source data and exact *p* values are provided as a Source Data file. Source data are provided as a Source Data file.

Supplementary Figure 8

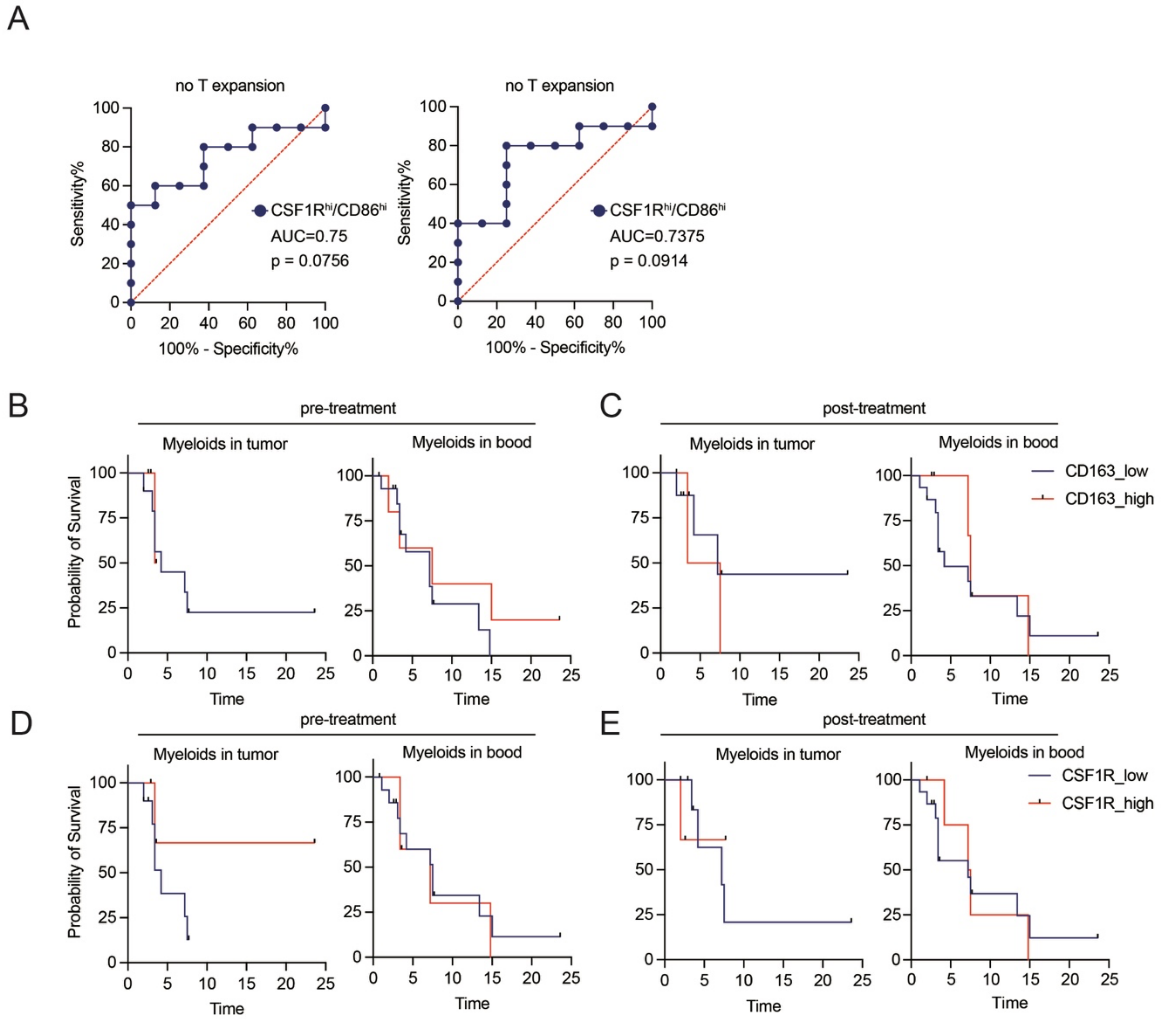

Supplementary Figure 8. Different prediction power of different macrophage markers.
(A) ROC curve demonstrating the ability of CD163^{hi}/CD86^{hi} ratio (left) and CSF1R^{hi}/CD86^{hi} ratio (right) to predict patients who did not achieve T cell expansion after anti-PD1 treatment. T cell expansion information was adopted from original publication. CD86_high or CD163 high cells were identified by CD86 expression or CD163 expression higher than the 25th percentile respectively. Malignant cell annotation was adopted from the original publication. CD163_low cells were the cells neither in CD86_high group nor in CD163_high group. n=18. *p* values are from Receiver operating characteristic curve (ROC).
(B) Kaplan-Meier relapse-free survival curves of GSE169246 patients based on CD163^{hi}/CD86^{hi} ratio in tumor (left) and blood (right) of pre-treatment tumors. Left, n=14. Right, n=20. *p* values are from two-sided Mann-Whitney test.
(C) Kaplan-Meier relapse-free survival curves of GSE169246 patients based on CD163^{hi}/CD86^{hi} ratio in tumor (left) and blood (right) of post-treatment tumors**.** Left, n=14. Right, n=20. *p* values are from two-sided Mann-Whitney test.
(D) Kaplan-Meier relapse-free survival curves of GSE169246 patients based on CSF1R^{hi}/CD86^{hi} ratio in tumor (left) and blood (right) of pre-treatment tumors**.** CD86_high or CSF1R high cells were identified by CD86 expression or CSF1R expression higher than the 25th percentile respectively. Malignant cell annotation was

adopted from the original publication. CSF1R_low cells were the cells neither in CD86_high group nor in CSF1R_high group. Left, n=14. Right, n=20. *p* values are from two-sided Mann-Whitney test. (E) Kaplan-Meier relapse-free survival curves of GSE169246 patients based on $CSF1R^{hi}/CD86^{hi}$ ratio in tumor (left) and blood (right) of post-treatment tumors. Survival information was adopted from the original publication. *p* values are from two-sided Mann-Whitney test. Left, n=14. Right, n=20. Source data are provided as a Source Data file.

Supplementary Table 1: primers for ChIP-PCR

C5aR1_TF2_f	TCT CAA CCT CCC CCT TCC AAA
C5aR1_TF2_r	TGC AGT CAC AGA GAC CCA CAA
C5aR1_TF3_f	GTT GGG TTA AGC AAG CCA CAG
C5aR1_TF3_r	GAC TGG GCA CAG ACC AAA CC
C5aR1_TF4_f	ATC TCC ATG AGT TCA AGG CTA CC
C5aR1_TF4_r	GCC TCA TTA TGT AAC TTG GCT GAC T

Supplementary Table 2: gene list used for functional pathway scores

Anti-tumor cytokine	Il1b ,Il2 ,Il12b ,Il1a ,Tnf ,Cxcl9 ,Cxcl10 , Ifng
Pro-tumor cytokine	Il10, Tgfb1 , Ccl17, Ccl3, Ccl22, Ccl24, Ccl2, Vegfa, Il4, Cxcl15, Cxcl1, Cxcl2, Cxcl3, Csf1, Csf2, Csf3, Ptgs2, Ptges2
Antigen presentation	Mill1, Cd1d1, Cd74, Mill2, Relb, H2-T23, H2-T22, Gm11127, Gm7030, H2-T10, Gm8909, H2-T3, H60c, H2-M10.2, H2-M10.3, H2-M10.1, H2-M10.4, H2-M11, H2-M9, H2-M1, H2-M10.5, H2-M10.6, H10b, Raet1e, Raet1d, H2-M5, H2-M3, H2-M2, Ap3b1, Fcgrt, Ifng, Hfe, Psmb9, Procr, Psmb8, H2-Qb, H2-Ab1, H2-Aa, H2-Eb1, H2-Eb2, Mr1, H2-Q1, H2-Q2, H2-Q4, H2-Q6, H2-Q7, H2-Q10, Azgp1
