# Supplementary material for: C5aR1 inhibition reprograms tumor associated macrophages and reverses PARP inhibitor resistance in breast cancer
Source: Nat Commun. 2024 May 27;15:4485. doi: 10.1038/s41467-024-48637-y (PMC11130309; doi:10.1038/s41467-024-48637-y)
Supplement: Supplementary file 3 — Description of Additional Supplementary Files [file 41467_2024_48637_MOESM3_ESM.pdf]

Description of Additional Supplementary Files

Supplementary Data 1: Top 25 DEG of each annotated cluster.
