# Supplementary material for: C5aR1 inhibition reprograms tumor associated macrophages and reverses PARP inhibitor resistance in breast cancer
Source: Nat Commun. 2024 May 27;15:4485. doi: 10.1038/s41467-024-48637-y (PMC11130309; doi:10.1038/s41467-024-48637-y)
Supplement: Supplementary file 5 — Reporting Summary [file 41467_2024_48637_MOESM5_ESM.pdf]

nature portfolio

Corresponding author(s): Xi Li

Last updated by author(s): May 9, 2024

Reporting Summary

Nature Portfolio wishes to improve the reproducibility of the work that we publish. This form provides structure for consistency and transparency in reporting. For further information on Nature Portfolio policies, see our Editorial Policies and the Editorial Policy Checklist.

Statistics

For all statistical analyses, confirm that the following items are present in the figure legend, table legend, main text, or Methods section.

n/a	Confirmed	
☐	☒	The exact sample size (*n*) for each experimental group/condition, given as a discrete number and unit of measurement
☐	☒	A statement on whether measurements were taken from distinct samples or whether the same sample was measured repeatedly
☐	☒	The statistical test(s) used AND whether they are one- or two-sided<br>*Only common tests should be described solely by name; describe more complex techniques in the Methods section.*
☐	☒	A description of all covariates tested
☐	☒	A description of any assumptions or corrections, such as tests of normality and adjustment for multiple comparisons
☐	☒	A full description of the statistical parameters including central tendency (e.g. means) or other basic estimates (e.g. regression coefficient) AND variation (e.g. standard deviation) or associated estimates of uncertainty (e.g. confidence intervals)
☐	☒	For null hypothesis testing, the test statistic (e.g. *F*, *t*, *r*) with confidence intervals, effect sizes, degrees of freedom and *P* value noted<br>*Give P values as exact values whenever suitable.*
☒	☐	For Bayesian analysis, information on the choice of priors and Markov chain Monte Carlo settings
☒	☐	For hierarchical and complex designs, identification of the appropriate level for tests and full reporting of outcomes
☐	☒	Estimates of effect sizes (e.g. Cohen's *d*, Pearson's *r*), indicating how they were calculated

Our web collection on statistics for biologists contains articles on many of the points above.

Software and code

Policy information about availability of computer code

Data collection	MDST tumors were disaggregated by gentleMACS kits (Miltenyi Biotec, #130-096-730), and incubated in TotelseqA hashtag antibodies (Biolegend, #394601, #394603, #394605, #394607, #394609, #394611, #394613, #394615) following the manufactural instructions. Live cells were isolated by EasySep Dead Cell Removal (Annexin V) Kit (STEMCELL Technologies, #17899). Single cell suspensions were processed according to 10xGenomics scRNAseq sample preparation protocol (Chromium Single Cell 3' v3.1 Reagent Kit, and 3' CellPlex Kit Set A, 10xGenomics). Sequencing was done by Novaseq 6000.
Data analysis	SCANPY (v1.7.1), scVelo (v0.2.3), Cell Ranger (v6.0.0), GSVA (1.40.1), Survival R (v3.3-1), FlowJo (v10.4), samtools (v1.11), ImageJ (v1.52a), Prism (v8.0.2), CellChat (v1.1.3), pySCENIC (v0.11.2), scPred (v1.9.2). This study did not generate custom code, and the wrapped code used in the study is freely available from https://github.com/xili3367/Left-and-right-co-transplantation.

For manuscripts utilizing custom algorithms or software that are central to the research but not yet described in published literature, software must be made available to editors and reviewers. We strongly encourage code deposition in a community repository (e.g. GitHub). See the Nature Portfolio guidelines for submitting code & software for further information.

Data

Policy information about availability of data

All manuscripts must include a data availability statement. This statement should provide the following information, where applicable:

- Accession codes, unique identifiers, or web links for publicly available datasets
- A description of any restrictions on data availability
- For clinical datasets or third party data, please ensure that the statement adheres to our policy

The mouse-derived syngeneic transplant sequencing data generated in this study have been deposited in the GEO database under accession code GSE215908 [https://www.ncbi.nlm.nih.gov/geo/query/acc.cgi?acc=GSE215908]. The data of cancer cell lines used in this study are available in the GEO database under accession code GSE157220 [https://www.ncbi.nlm.nih.gov/geo/query/acc.cgi?acc=GSE15722].
The public human data of TNBC used in this study are available in the European Genome-phenome Archive (EGA) under accession code EGAD00001006608 [https://ega-archive.org/studies/EGAS00001004809] and in the GEO database under accession code GSE169246 [https://www.ncbi.nlm.nih.gov/geo/query/acc.cgi?acc=GSE169246].
Public RNA-seq dataset for breast cancer in the public database The Cancer Genome Atlas (TCGA, National Cancer Institute (NCI), Bethesda, MD, USA) were downloaded from the GDC portal (https://www.cbioportal.org/study/summary?id=brca_tcga_pub)

The remaining data are available within the Article, Supplementary Information or Source Data file. Source data are provided with this paper.

Research involving human participants, their data, or biological material

Policy information about studies with human participants or human data. See also policy information about sex, gender (identity/presentation), and sexual orientation and race, ethnicity and racism.

Reporting on sex and gender	n/a
Reporting on race, ethnicity, or other socially relevant groupings	n/a
Population characteristics	n/a
Recruitment	n/a
Ethics oversight	n/a

Note that full information on the approval of the study protocol must also be provided in the manuscript.

Field-specific reporting

Please select the one below that is the best fit for your research. If you are not sure, read the appropriate sections before making your selection.

☒ Life sciences ☐ Behavioural & social sciences ☐ Ecological, evolutionary & environmental sciences

For a reference copy of the document with all sections, see nature.com/documents/nr-reporting-summary-flat.pdf

Life sciences study design

All studies must disclose on these points even when the disclosure is negative.

Sample size	For mouse assays, n>=5 was chosen as the minimal replicate number based on the statistical power of detecting meaningful effects. For ChIP-pcr, assay was done with 3 technology replicates. For single cell RNA sequencing, n=2 was chosen for vehicle groups, n=3 was chosen for the minimal sample number for treatment groups.
Data exclusions	For single cell analysis: cells with fewer than 400 genes detected (UMI > 1000) or with more than 20% UMIs from mitochondrial genes were excluded by SCANPY . Doublets were removed by Scrublet with the expected doublet rate of 6 and doubletScore larger than 95%.<br>For other assays in the manuscript: no sample was excluded from the analysis.
Replication	our methods of replication were all successful. The efficacy of PMX53 combined with olaparib was recapitulated in independent mouse models and was also confirmed by flow cytometry.
Randomization	Mice after tumor transplantation or tumor injection were randomly distributed to different treatment groups.
Blinding	No blinding was performed in this study. But the cell type assignment of scRNA-seq data was based on automated analysis.

Reporting for specific materials, systems and methods

We require information from authors about some types of materials, experimental systems and methods used in many studies. Here, indicate whether each material, system or method listed is relevant to your study. If you are not sure if a list item applies to your research, read the appropriate section before selecting a response.

Materials & experimental systems

n/a	Involved in the study	
☐	☒	Antibodies
☐	☒	Eukaryotic cell lines
☒	☐	Palaeontology and archaeology
☐	☒	Animals and other organisms
☒	☐	Clinical data
☒	☐	Dual use research of concern
☒	☐	Plants

Methods

n/a	Involved in the study	
☒	☐	ChIP-seq
☐	☒	Flow cytometry
☒	☐	MRI-based neuroimaging

Antibodies

Antibodies used

Cells were stained with CD45-BV711 (Biolegend, 103147, 0.125µg), CD3-APC (Biolegend, 100236, 0.125µg), CD8-AF532 (Invitrogen, 58-0081-80, 0.125µg), CD335-PerCP-efluor710 (Invitrogen, 46-3351-82, 0.125µg), F4/80-BUV563 (Invitrogen, 365-4801-82, 0.25µg), I-A/I-E-APC-Cy7 (Biolegend, 107627, 0.0625µg), CD206-PE-eFluor610 (Thermo, 61-2061-82, .0625µg) antibody, secondary goat anti-rat antibody conjugated with Alexa Fluor 555 (Thermo, A-21434, 1:500) and C5aR1 (Santa Cruz, sc-271949, 0.5µg), GZMB-AlexaFluor594 (Fisher, IC2906T100UG, 0.5µg), PRF1-PE (eBioscience, 12-9392-82, 0.5µg) and IFNγ-BUV395 (eBioscience, 363-73311-82, 0.5µg).

Validation

CD45-BV711 (Biolegend, 103147), CD3-APC (Biolegend, 100236) ,I-A/I-E-APC-Cy7 (Biolegend, 107627) and CSF1R (Biolegend, 135501) have been validataed for flow cytometry as stat4ed on Biolegend product page. CD8-AF532 (Invitrogen, 58-0081-80), CD335-PerCP-efluor710 (Invitrogen, 46-3351-82) and CD206-PE-eFluor610 (Thermo, 61-2061-82) have been validataed for flow cytometry as stat4ed on ThermoFisher product page. C5aR1 (Santa Cruz, sc-271949) has been validated for western blotting and IHC as stated on SANTA CRUZ product sheet.

Eukaryotic cell lines

Policy information about cell lines and Sex and Gender in Research

Cell line source(s)

State the source of each cell line used and the sex of all primary cell lines and cells derived from human participants or vertebrate models.

Authentication

Describe the authentication procedures for each cell line used OR declare that none of the cell lines used were authenticated.

Mycoplasma contamination

Confirm that all cell lines tested negative for mycoplasma contamination OR describe the results of the testing for mycoplasma contamination OR declare that the cell lines were not tested for mycoplasma contamination.

Commonly misidentified lines
(See ICLAC register)

Name any commonly misidentified cell lines used in the study and provide a rationale for their use.

Animals and other research organisms

Policy information about studies involving animals; ARRIVE guidelines recommended for reporting animal research, and Sex and Gender in Research

Laboratory animals

Female FVB and C57BL/6 mice at 6 weeks of age were purchased from the Jackson Laboratory.

Wild animals

No wild animals were used in this study

Reporting on sex

Only female mice were used in this study.

Field-collected samples

No field-collected samples were used in this study.

Ethics oversight

Animal experiments were performed in the AALAC approved Animal Facility, Knight cancer Institute, Oregon Health & Science University under IACUC protocol: Combination Therapy Targets Adaptive Resistance in Cancer (TR01_IP00002062).

Note that full information on the approval of the study protocol must also be provided in the manuscript.

Plants

Seed stocks

Report on the source of all seed stocks or other plant material used. If applicable, state the seed stock centre and catalogue number. If plant specimens were collected from the field, describe the collection location, date and sampling procedures.

Novel plant genotypes

Describe the methods by which all novel plant genotypes were produced. This includes those generated by transgenic approaches, gene editing, chemical/radiation-based mutagenesis and hybridization. For transgenic lines, describe the transformation method, the number of independent lines analyzed and the generation upon which experiments were performed. For gene-edited lines, describe the editor used, the endogenous sequence targeted for editing, the targeting guide RNA sequence (if applicable) and how the editor was applied.

Authentication

Describe any authentication procedures for each seed stock used or novel genotype generated. Describe any experiments used to assess the effect of a mutation and, where applicable, how potential secondary effects (e.g. second site T-DNA insertions, mosiacism, off-target gene editing) were examined.

Flow Cytometry

Plots

Confirm that:

- [x] The axis labels state the marker and fluorochrome used (e.g. CD4-FITC).
- [x] The axis scales are clearly visible. Include numbers along axes only for bottom left plot of group (a 'group' is an analysis of identical markers).
- [x] All plots are contour plots with outliers or pseudocolor plots.
- [x] A numerical value for number of cells or percentage (with statistics) is provided.

Methodology

Sample preparation	Cells were isolated with gentleMACS kits (Miltenyi Biotec, #130-096-730). Cells were washed with cold staining buffer (Biolegend), followed by resuspension of 106 cells in 100µl cold staining buffer (Biolegend, #420201) with PI or Calcein Violet AM as live/dead cell indicators. Cells were stained with CD45-BV711 (Biolegend, 103147), CD3-APC (Biolegend, 100236), CD8-AF532 (Invitrogen, 58-0081-80), CD335-PerCP-efluor710 (Invitrogen, 46-3351-82), F4/80-BUV563 (Invitrogen, 365-4801-82), I-A/I-E-APC-Cy7 (Biolegend, 107627), CD206-PE-eFluor610 (Thermo, 61-2061-82) antibody, secondary goat anti-rat antibody conjugated with Alexa Fluor 555 (Thermo, A-21434) and C5aR1 (Santa Cruz, sc-271949) or CSF1R (Biolegend, 135501) for 30 minutes on ice.
Instrument	Cells were analyzed by Aurora Cytek Analyzer.
Software	All analysis was performed in FlowJo (v10.6.1).
Cell population abundance	5000 live cells of each tumor were gated for further analysis.
Gating strategy	Gating strategy was indicated in the supplementary figures. Briefly, cells were sorted by FSC and SSC to obtain single cells, then sorted for PI negative (liver) versus PI positive (dead.) for further analysis.

- [x] Tick this box to confirm that a figure exemplifying the gating strategy is provided in the Supplementary Information.
