# Supplementary material for: C5aR1 inhibition reprograms tumor associated macrophages and reverses PARP inhibitor resistance in breast cancer
Source: Nat Commun. 2024 May 27;15:4485. doi: 10.1038/s41467-024-48637-y (PMC11130309; doi:10.1038/s41467-024-48637-y)
Supplement: Supplementary file 2 — Peer Review File [file 41467_2024_48637_MOESM2_ESM.pdf]

nature portfolio

Peer Review File

C5aR1 inhibition reprograms tumor associated macrophages and reverses PARP inhibitor resistance in breast cancer

Open Access This file is licensed under a Creative Commons Attribution 4.0 International License, which permits use, sharing, adaptation, distribution and reproduction in any medium or format, as long as you give appropriate credit to the original author(s) and the source, provide a link to the Creative Commons license, and indicate if changes were made. In the cases where the authors are anonymous, such as is the case for the reports of anonymous peer reviewers, author attribution should be to 'Anonymous Referee' followed by a clear attribution to the source work. The images or other third party material in this file are included in the article's Creative Commons license, unless indicated otherwise in a credit line to the material. If material is not included in the article's Creative Commons license and your intended use is not permitted by statutory regulation or exceeds the permitted use, you will need to obtain permission directly from the copyright holder. To view a copy of this license, visit http://creativecommons.org/licenses/by/4.0/.

REVIEWERS' COMMENTS:

Reviewer #1 (Remarks to the Author): with expertise in scRNAseq, immunology

Li et al. studied the mechanisms underlying the sensitivity and resistance to PARPi in the murine breast cancer model. They found that the PARPi-sensitive tumor model enriched more M1 compared to M2 macrophages, while the PARPi-resistant tumor models harbored a lower level of M1 macrophages. Moreover, the authors identified that C5aR1 signaling contributed to the elevated M2 macrophages in PARPi-resistant tumors. They further revealed that targeting C5aR1 could increase the sensitivity of tumors to PARPi via selective depletion of M2 macrophages. This work provides a valuable therapeutic role of C5aR1. However, a few points should be addressed, especially for the single-cell analyses of tumor-associated macrophages.

Here are my main concerns.

1. For single-cell studies, the thorough annotation of different clusters is critical to draw biological or clinically relevant conclusions. However, the cell types in this study are less characterized, and the annotations are vague. Specifically, the entire manuscript focused on the alterations of Macro-M1 and Macro-M2, but these cells showed barely discriminated signatures among myeloid clusters, according to Figures 1A and 1D. The authors should compare their myeloid cells with those from other murine models by single-cell analyses. It will aid in characterizing their clusters more precisely.

2. Although the M1 and M2 polarization of macrophages is well established in vitro models, the authors should provide more evidence that macrophages in their tumor models fit the conventional M1/M2 models. The concern is that several single-cell studies demonstrated that TAM exhibited both M1 and M2 phenotypes in tumors such as breast cancer and colorectal cancer.

3. As the authors pointed out in their discussion that C5aR1+ neutrophils also contribute to the poor survival of breast cancer. They should clarify whether their observations were attributed to C5aR1+ neutrophils or not.

4. The experimental design needs to be more specific. For example, the authors should provide the rationale for their models' two-week gap between two transplants.

Minor concerns:
1. The authors should add detailed parameters for single-cell analyses and bioinformatic tools in the Methods.
2. As demultiplexing is mentioned in the data processing, the authors should provide details on how they mixed the samples.
3. The P-values or significance labels are missed in Figure 3E.

Reviewer #2 (Remarks to the Author): with expertise in breast cancer, immunology, macrophages

Li et al. present a manuscript entitled: Single cell RNA sequencing reveals C5aR1 inhibition to selectively target pro-tumorigenic M2 macrophages reversing PARP inhibitor resistance. The team used a PARPi-sensitive and a PARPi-resistant murine model of breast cancer. They claim that the PARPi-sensitive model has a high ratio of M1/M2 macrophages, and the M1/M2 macrophage ratio is increased after PARPi therapy. The PARPi-resistant model has low M1 macrophages and a low M1/M2 ratio; which is not altered by PARPi therapy. The team claims that when the sensitive and resistant tumors are co-implanted into the same mouse the sensitive tumors accumulate M2 macrophages and it becomes resistant to PARPi therapy. The team identifies that C5ar1 is elevated in the macrophages associated with resistance. They claim that therapeutic targeting of C5ar1 was effective at depleting the M2 macrophages but left the M1 macrophages, which sensitized the resistant tumors to PARPi therapy. Human breast tumors with high expression of C5aR1 were associated with poor responses to ICB. This work has important clinical implications as PARPi resistance is a major unmet need (albeit among patients with BRCA- and HR-associated cancers). This manuscript has many significant flaws which need to be addressed.

Several major limitations are found in this work:
1. It is unclear why there is a focus on PARPi. PARPi are only FDA approved for use in patients with BRCA- and HR- deficient cancers and the studies so far have shown there is no

benefit for BRCA- and HR – proficient patients. Why wasn't ICB used in this model? Or chemotherapy? Is this a PARPi-specific effect?

a. The models do not accurately represent patients being treated with PARPi since the models are brca- and hr – proficient. This study claims to address an unmet need but it does not. While there is clinical interest in using PARPi outside of BRCA and HR – deficient tumors, it is not currently FDA approved in breast cancer for that use and there is minimal rationale that the drug will be effective in this setting. PARPi-resistance largely stems from brca reversions and a number of other alterations (Li et al. Molecular Cancer 2020; Wicks et al. Open Biol 2022; Baxter et al. Mol. Oncol. 2022), which is not the case with this model.

b. The team suggests that TNBC patients who have a worse response to ICB have higher expression of C5aR1 however this is disconnected from their main findings that C5aR1+ macrophages are driving PARPi resistance.

c. How are the cancer cells dying in response to the drug if they are not undergoing synthetic lethality?

2. A major weakness and detrimental limitation to the study as a whole was the generalized used of M1 and M2, which are not biological states of a macrophage in a tumor. There is very poor macrophage biology described in this paper. Since the focus is entirely on tumor macrophages the authors need to address the complexity and heterogenous nature of tumor macrophages. The complexities of tumor macrophages have been studied extensively and this work should incorporate the heterogeneity of these cells. The M1/M2 dichotomy is outdated when describing macrophages in tissues. M1 and M2 definitions are macrophages cultured ex vivo in either LPS/IFNg (M1) or some combination of IL-4/10/13 (M2). Macrophages in a tumor do not represent either M1 or M2, but likely a mix of both states. Using the terms M1/M2 throughout this paper seriously delays progress in this field and renders the interpretation of the data meaningless.

a. Marker and functional characterizations of macrophage populations need to be used. There are several clusters of macrophages in the UMAPs, which should be characterized by marker expression.

b. In S2A authors show use CSF1R, CD2026 or C5AR1 to define a "M2" population but state throughout the text that CSf1R is both M1 and M2. There is so much confusion in this manuscript regarding what macrophages are being called and referred to that

interpretation is very difficult. Authors need to call populations of macrophages as unique subsets and call them by their marker expression and not “M1/M2”.

3. Several comparisons between CSF1R and C5aR1 are made throughout the manuscript, likely due to a previously published paper showing that CSF1R+ macrophages contribute to PARPi resistance (Mehta et al. Nat. Can 2021). There are too much unclear, unsubstantiated comparisons, ie claims made without statistics. It’s not clear why there needs to be a focus on the comparison. The comparison between CSF1R and C5aR1 causes confusion

a. The data shown here indicate that Ola increases both CSF1R+ macrophages and C5aR1+ macrophages after PARPi. These populations tend to follow a similar trend (Fig. 2F-G). Are these populations overlapping? What is the extent of double positive cells (CSF1R+C5aR1+)?

b. Line 427-428; CSF1R appears to be associated with M1 and M2; as does C5ar1 from S4A.

c. The authors claim that CSF1R “may contribute to M1 polarization” but there is no real data to show this. Just speculation. The data shown suggests that CSF1R is expressed in the same population of macrophages as C5aR1(high) (CSF1R and C5ar1 is shown in both M1 and M2 populations in Fig 1D; Fig 6G and 6J CSF1R is highest in the CfaR1 high population). Since it is a fairly well published and accepted that CSF1R+ macrophages are associated with a suppressive phenotype, the authors would need to back up their claims with solid data, functional studies and statistics to show that CSF1R is more “M1-like”. Otherwise the authors need to not over state/ over interpret their data. This is a very confusing point they try to make, and their data does not support the claim.

d. Additionally, the authors show that C5aR1 is expressed in both “Macro-M1” and “Macro-M1” (and Macro-others and Macro-MKI67), making it a pan macrophage marker. It is not clear why they try to suggest that C5aR1 is only on their M2s and CSF1R is on both of their M1s and M2s. Subclustering the macrophage populations and calling them by marker expression and function would potentially provide clarity to the heterogeneity.

Other concerns:

1. The single cell data/flow cytometry in Fig 1-2 has several concerns.

a. Only 2 vehicle mice were used. Statistics can not be run with an n of 2. Authors must include additional mice in order to run statistical analysis.

b. In Figure 2A it appears there are more Macro-M2 in both the T127 and T22 tumors compared to their “M1”. The “M2” do not appear to change after treatment. The abstract reads as though there are more M1 macrophages in the tumors. This is not true. The

abstract needs to be revised to reflect the baseline infiltration of macrophages as there are actually more M2's in the tumor before and after treatment.
The text causes confusion and is misleading.
c. It is not clear how the authors are defining "M1" and "M2" by flow cytometry in Fig. 2B (and throughout, this needs to be clearly defined by markers). Is it the use of S2A or S2C? They show different marker uses for "M2".
d. *Legend for S2A is wrong related to which figures the flow corresponds to.
e. In 2B the analysis of macrophage populations out of total cells should be performed and shown to indicate total numbers of these cells (not the proportion out of macrophages, which is what is currently shown).
f. Authors state in 335-337 that T22 had fewer macrophages, but that is not what is shown. Only the proportion out of macrophages is shown. Please revise to reflect what is shown and include the data showing numbers of macrophages out of total cells (as previous comment indicates).
2. Define what "altered functionality" means in line 445.
3. Fig 4 C: does not line up with previously shown data including that shown in Fig 2B comparing veh and ola. Fig 4C shows significant increase in their "m2" macrophages after Ola in T127s and no change in T22s; whereas Fig 2B shows no change in "m2" in T127 and decrease in m2 in T22. This discrepancy needs to be explained.
4. Data shown in S6C is not interpretable because the Ola did not work the same as in Fig 3B. S6C shows no effect of Ola on either the T22 or T127 however in the experiment with the PMX53 compound there was a reduction in tumor burden with Ola monotherapy shown in Fig 3B.
5. It's not clear how or why C5ar1 and CSF1r would be inversely correlated in Fig 5C when Fig 1D (and other figure) show that the macrophage populations express both receptors.
6. Supl Fig 7H: csf1 and il34 are ligands for csf1r. Is CSF1R also upregulated in this assay? This experiment seems out of context and the connection to C5aR1 is confusing. I suppose IL34 is making the macrophages more M2-like and therefore express C5aR1, but wouldn't these macrophages also express CSF1R?
7. Authors need to support the claim that C5aR1 macrophages are suppressive through ex vivo functional studies and genetic manipulation.

Other minor concerns:

1. Figure 1B needs stats with average tumor volume curves. The text compares T22 and T127 but they are graphed on separate graphs. Conclusions are hard to make without proper statistical analysis. It is not clear what the gray colors of the circles are meant to indicate in Fig 1A.

2. S1A-C is difficult to see; consider darker colors (S4C looks much better).

3. Fig 3E: show the M1/M2 ratio below the graph to indicate the ratio of increased M2s.

4. Figure 5 is blurry.

5. Text indicates an “other” macrophage cluster in Fig 6B-C, but is not shown in the figure.

Reviewer #3 (Remarks to the Author): with expertise in PARPi

In this manuscript, Li et al. report a new mechanism of resistance to the PARP1/2 inhibitor olaparib. The authors show that C5aR1 positive M2 macrophages are mediating both local and distant therapy resistance to olaparib in a syngeneic murine breast cancer model. Accordingly, the authors proposed that C5aR1 inhibition may be a good therapeutic strategy to sensitize tumour to olaparib.

Major comments

1.- In co-transplantation models, both T22 and T127 tumours grow faster than when implanted individually, regardless of whether they are treated with olaparib or vehicle. What mechanisms may be involved?

2.- Olaparib, which inhibits both PARP1 and PARP2, is used throughout the work. A selective PARP1 inhibitor is now available (AZ5305; Illuzzi et al., 2022), clinical trials are ongoing, and is commercially available. It would be interesting, using this new inhibitor, to determine the specific role of PARP1 and to assess whether the proposed therapeutic combination for olaparib can be extended to the new generation of selective PARP1 inhibitors.

3.- In the T127 model, in figure 1B, a large variability is observed, especially in response to olaparib treatment, with some mice being "sensitive" and others more resistant. How can this large experimental heterogeneity be explained? Has it been taken into account in any way in the following analyses?

4.- Data on the effect of olaparib on macrophage function have been estimated based on scRNAseq results. Similarly, the interaction between cell populations has been estimated

based on scRNAseq data. It would be interesting to validate these predictions.

Minor comments

Line 296.- Authors stated “See figure 1A for a list of samples assessed”. Figure 1A does not show a list of samples assessed for scRNAseq

2.- Figure 1B: T22 tumour growth curves are represent in the upper panel and T127 in the lower panel. However, the legend to the figure indicates “Tumor growth curve of T127 (upper) and T22 (lower)”.

3.- Legend Y-axis of Figure 1B, 3B, 3C, 4A, 4B, 4E, 4F: Tumor instead Tomur.

4.- The legend in Fig. 3B and 3C should be corrected as shown in the figure.

5.- The definition of the experimental groups in figure 4F is not clear, and does not coincide with the legend in figure 4F.

Reviewer #4 (Remarks to the Author): with expertise in PARPi, breast cancer

This report utilizes two syngeneic mouse tumors (T22 and T127) to assess the contribution of immune cell populations to PARP inhibitor (PARPi) sensitivity. Consistent with their previous publication, the authors note that T22 is sensitive to PARPi treatment upon transplantation into syngeneic recipient mice, whereas T127 is resistant. The ratio of M2 vs. M1-like macrophage populations also differs between these tumors. In line with published work, abundant M2 macrophages in T127 tumors is coincident with immune suppression and PARPi resistance. The authors analyze immune cells also by scRNAseq in both single and co-transplantation settings. Using such analysis, they suggest C5ar1 as a new receptor on M2 macrophages that may be important for the immune suppressive phenotype observed in such tumors. Finally, they draw upon data from patients that received immune checkpoint inhibitor treatment to assess whether C5ar1is indeed important for immune suppression in these tumors.

This is an interesting study; however, it has many fundamental technical shortcomings that significantly impact the validity of the conclusions drawn from the data. As such, it is currently unsuitable for publication.

Specific points

1. The authors indicate that both T22 (PARPi sensitive) and T127 (PARPi resistant) are both HR proficient due to the fact that they are BRCA1/2 wildtype (line 77-79). They need to provide this data on BRCA1/2 or reference appropriately. Their previous Cancer Research paper referenced here does not contain these data either. In addition, there are many other well-established ways by which cells can acquire HR deficiency independently of BRCA1/2 mutation. At a minimum, to prove that both tumor models are HR proficient, the authors must provide conclusive data on the mutation status of key HR genes, including but not limited to BRCA1/2 as well as another measure of HRD. This may encompass RAD51 repair foci analysis or HRD scoring by other means such as HRdetect. This is essential since the conclusion of the manuscript changes dramatically if there are differences in HR proficiency.

2. The co-transplantation approach is confusing. Clarification is needed here. The authors state that 10 or 14 days after transplantation, mice were assigned to treatment groups based on randomization by tumor size (line 107+108). How exactly and under what rationale was this carried out? This is especially confusing since they also state in the methods section that T22 cells were transplanted 14 days prior to T127 due to big differences in tumor growth rates (line 108). This is very important since throughout the manuscript with or without co-culture, both models seem to follow very similar trajectories upon palpation (Figure 1B, 3B+C for example).

3. Presentation of Figure 1B is not clear. Please present data as dots per time point and condition with appropriate error bars, equivalent to Figure 4A+B for example.

4. T22 seems to contain only 10-20% tumor cells within the recovered mass with more than 80% immune and somatic cells in some mice (Figure S1B). This is striking and the authors need to elaborate on how this relates to the makeup of tumors in patients or in similar mouse models. The immune cell populations are seemingly not consistent when the authors examine the same models. For example, M2 macrophages are present at the same level in scRNA analysis of T22/T127 in Figure S1B but differ dramatically in Figure S4B+D. These data are also not consistent with the associated flow cytometry data. In addition, the representative gating used for this cytometry (Figure S2, S3, S6) shows that the authors strategy was not appropriate, which may explain this discrepancy. They did not omit very

low FSC/SSC particulate (Figure S2A, S6E), which often is found in such samples in the form of debris and these. It is necessary to re-analyze these data and then re-assess their key conclusions regarding immune cell composition together with their existing scRNAseq data.

5. The authors then draw upon analysis of existing patient data to validate their key findings in TNBC tumors. They determine M2/M1 populations using unconventional markers and importantly, they elect C5ar1 as a marker to delineate M2/M1. This is not appropriate. The data, as presented, is explained by the fact that higher M2 macrophages, independently of C5ar1, correlate with poor survival as expected on the basis of published literature. Under this light, C5ar1 may be simply expressed at a higher level in such cells, without any functional consequence. This needs to be examined.

6. General importance of C5ar1 in M2/M1 balance and PARPi sensitivity needs to be confirmed using another independent syngeneic mouse model to validate the authors findings.

Some additional minor concerns exist:

It would be useful to analyze known receptor ligand interactions as a positive control in their bioinformatic analysis presented in Fig 2, Fig S2+3.

Figure 2E is in apparent conflict with statements made in line 373+374. Olaparib treatment decreased C5ar1 expression in T22 single transplant tumors. This needs to be acknowledged and discussed in relation to their later findings in Figure 2F+G.

The significant NK cell populations detected in tumors in Figure S4B do not appear to be present in the same tumor model presented in Figure S1. How is this possible?

The authors need to confirm that C5ar1 inhibitor works equally well in their T22/T127 tumor models to supplement Figure 4.

Reviewer's Comments:
Reviewer #1 (Remarks to the Author)

Li et al. studied the mechanisms underlying the sensitivity and resistance to PARPi in the murine breast cancer model. They found that the PARPi-sensitive tumor model enriched more M1 compared to M2 macrophages, while the PARPi-resistant tumor models harbored a lower level of M1 macrophages. Moreover, the authors identified that C5aR1 signaling contributed to the elevated M2 macrophages in PARPi-resistant tumors. They further revealed that targeting C5aR1 could increase the sensitivity of tumors to PARPi via selective depletion of M2 macrophages. This work provides a valuable therapeutic role of C5aR1. However, a few points should be addressed, especially for the single-cell analyses of tumor-associated macrophages.

Here are my main concerns.
Reviewer #1:
1. For single-cell studies, the thorough annotation of different clusters is critical to draw biological or clinically relevant conclusions. However, the cell types in this study are less characterized, and the annotations are vague. Specifically, the entire manuscript focused on the alterations of Macro-M1 and Macro-M2, but these cells showed barely discriminated signatures among myeloid clusters, according to Figures 1A and 1D. The authors should compare their myeloid cells with those from other murine models by single-cell analyses. It will aid in characterizing their clusters more precisely.

Thank you for this comment; this is indeed an important point that we have now clarified in the revised manuscript. In the initial text, Figure 1D demonstrates clustering based on UMAP using scPred [1] trained on human cells for typing of T cells and macrophage and markers for typing of other cells from the single cell data with the key markers used for each cellular lineage indicated in Figure 1E [2] (https://github.com/xili3367/Left-and-right-co-transplantation). UMAP clustering shows that macrophages are a major component of the tumors. Figure 1E shows that a subset of marker overlaps in M1-like and M2-like macrophages. However, a subset of markers is dominantly expressed in the M1-like and the M2-like macrophages in Figure 1E. We have added an expansion of the macrophage subtyping markers to New Figure S1F using canonical macrophage markers defined by studies of murine macrophages [3-5]. This new Figure shows the differential expression of RNAs associated with different macrophage phenotypes. It also demonstrates that there are two macrophage populations that do not fit either the M1-like or M2-like macrophage designation from scPred.

As indicated in the new Figure S1F below, the M1-like population expresses high levels of canonical markers associated with the M1 anti-tumor/pro-inflammatory phenotype with low levels of the majority of the canonical M2 pro-tumor markers but with high levels of the subset of the M2 associated protumor markers. The M2-like population expresses lower levels than the M1-like population of most of the canonical markers associated with M1 anti-tumor/pro-inflammatory phenotype and high levels of the majority of the canonical M2/pro-tumor markers. This is consistent with previous studies demonstrating differences between human and murine macrophage polarization markers as well as differences between in vitro and in vivo macrophage polarization markers. Interestingly, the expression of the canonical markers in the M1-like and M2-like macrophages were to a major degree mutually exclusive consistent with these being different macrophage populations.

Response to reviewers Figure 1: Canonical M1 and M2 macrophage marker expression of each subtype of macrophages.

F

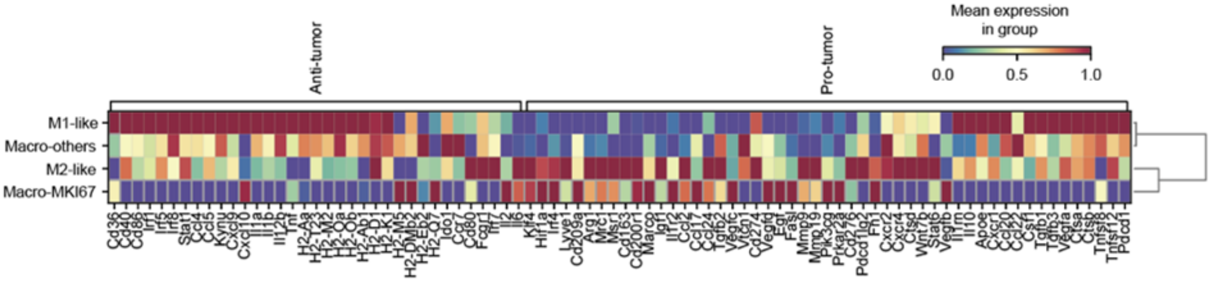

We have further compared our analysis of myeloid cells to those from other studies as suggested and the comparison supports the subtyping presented above. However, in comparison to the publicly available mouse scRNA-seq datasets GSE142213 and GSE141017, while there was an overlap with the M1-like macrophages, there was limited correlation with M2-like macrophages See Figure 2 below. This is like due to differences between M1 and M2 macrophages in vitro, in vivo and in specific murine models. This supports our designation of macrophages of M1-like and M2-like throughout the manuscript.

Response to reviewers Figure 2: Jaccard index between macrophage subtypes from our datasets and GSE142213, GSE141017. The M1 or M2 macrophages in GSE142213 and GSE141017 were annotated by MetaTiME from an independent bioinformatics algorithm [6].

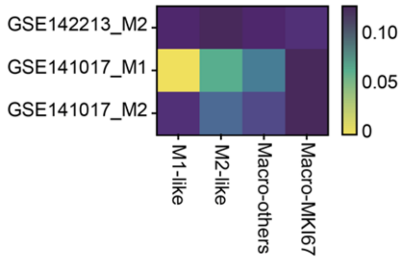

2. Although the M1 and M2 polarization of macrophages is well established in vitro models, the authors should provide more evidence that macrophages in their tumor models fit the conventional M1/M2 models. The concern is that several single-cell studies demonstrated that TAM exhibited both M1 and M2 phenotypes in tumors such as breast cancer and colorectal cancer.

Thank you for the suggestion; we agree that the dichotomy of M1 and M2 macrophages that have been developed in vitro are not fully recapitulated in vivo. We have evaluated the expression pattern of canonical M1/M2 markers in the new Figure S1F, although the M1-like TAM do express high levels of some canonical M2 markers and vice versa, the two populations identified in the single cell data do dominantly express markers associated with the M1 and M2 phenotype. Thus, we use the terms M1-like and M2-like to describe the populations that phenotypically copy M1 and M2 macrophage function (not markers) throughout the manuscript. As we demonstrate later in the manuscript, there is likely transition and plasticity between the M1-like and M2-like macrophage designation. In the revised text, we indicate the potential that the M1 and M2 designation may not fully capture the phenotype of the macrophages in the tumor. Throughout the manuscript and the Figures, we carefully define the process by which macrophages are designated M1-like and M2-like. For example, in addition to clarifying the scPred approach, a detailed description of marker usage of differentiating M1/M2 by flow cytometry has been added to Figure S2A.

3. As the authors pointed out in their discussion that C5aR1+ neutrophils also contribute to the poor survival of breast cancer. They should clarify whether their observations were attributed to C5aR1+ neutrophils or not.

As suggested, we have addressed the potential role of C5aR1+ neutrophil effects in our datasets. However, there was no Fut4 expression which encodes CD15 or Cd177 detected in our datasets indicating lack of capture of in our single cell RNA Seq data. This is not unusual due to the “fragility” of neutrophils in terms of the isolation procedures used with tissues. We have modified the section in discussion in light of this observation.

4. The experimental design needs to be more specific. For example, the authors should provide the rationale for their models' two-week gap between two transplants.

Thank you for this important comment. We have updated the methods to include the following points describing the rationales related to the model establishment. The T22 model and the T127 model demonstrate different take and initial growth rates. To accommodate the differences in the time required for the tumors to become established and grow exponentially, we introduced the T22 model 2 weeks before the T127 model when T22 tumor reaches around $20mm^3$. Once the tumors are established, their growth rates are similar (see Figure 1B).

It is important to note that the T22 and T127 are transplantation models. They are thus much more heterogenous than tissue culture derived cell culture models. Indeed, they were selected based on their heterogeneity of growth that more closely reflects human tumors than xenografts. In the revised text, we have presented the data as separated spider plots to emphasize the heterogeneity in growth of each tumor.

Minor concerns:
1. The authors should add detailed parameters for single-cell analyses and bioinformatic tools in the Methods.
Thank you for the suggestions. The details of the parameters and the bioinformatic tools used have been added in methods.

2. As demultiplexing is mentioned in the data processing, the authors should provide details on how they mixed the samples.
Thank you for the suggestions. The details of sample mixing and demultiplexing has been added in methods.

3. The P-values or significance labels are missed in Figure 3E.
Thank you for the important comments. However, Figure 3E majorly serves as a confirmation of Figure 1 and Figure 2 as well as exploring immune activity in a relative early phase of tumor development. Due to complexity of scRNA-seq multiplexing and large number of treatment groups, in some cases only 2 tumors were collected from a treatment group. The data should be viewed as “exploratory” without p values. The p values for the key experiments are presented in Figure 1 and Figure 2.

Reviewer #2 (Remarks to the Author):

Li et al. present a manuscript entitled: Single cell RNA sequencing reveals C5aR1 inhibition to selectively target pro-tumorigenic M2 macrophages reversing PARP inhibitor resistance. The team used a PARPi-sensitive and a PARPi-resistant murine model of breast cancer. They claim that the PARPi-sensitive model has a high ratio of M1/M2 macrophages, and the M1/M2 macrophage ratio is increased after PARPi therapy. The PARPi-resistant model has low M1 macrophages and a low M1/M2 ratio; which is not altered by PARPi therapy. The team claims that when the sensitive and resistant tumors are co-implanted into the same mouse the sensitive tumors accumulate M2 macrophages and it becomes resistant to PARPi therapy. The team identifies that C5ar1 is elevated in the macrophages associated with resistance. They claim that therapeutic targeting of C5ar1 was effective at depleting the M2 macrophages but left the M1 macrophages, which sensitized the resistant tumors to PARPi therapy. Human breast tumors with high expression of C5aR1 were associated with poor responses to ICB. This work has important clinical implications as PARPi resistance is a major unmet need (albeit among patients with BRCA- and HR-associated cancers). This manuscript has many significant flaws which need to be addressed.

Several major limitations are found in this work:
1. It is unclear why there is a focus on PARPi. PARPi are only FDA approved for use in patients with BRCA- and HR- deficient cancers and the studies so far have shown there is no benefit for BRCA- and HR – proficient patients. Why wasn't ICB used in this model? Or chemotherapy? Is this a PARPi-specific effect?
a. The models do not accurately represent patients being treated with PARPi since the models are brca- and hr – proficient. This study claims to address an unmet need but it does not. While there is clinical interest in using PARPi outside of BRCA and HR – deficient tumors, it is not currently FDA approved in breast cancer for that use and there is minimal rationale that the drug will be effective in this setting. PARPi-resistance largely stems from brca reversions and a number of other alterations (Li et al. Molecular Cancer 2020; Wicks et al. Open Biol 2022; Baxter et al. Mol. Oncol. 2022), which is not the case with this model.

We have revised the introduction to cover some of these questions. However, only looking for resistance and sensitivity to drugs following FDA approval or in FDA approved indications has the potential to miss the opportunity that is provided by drug combinations [8-11]. Indeed, many drug combinations have demonstrated activity where monotherapy has modest activity. Further, it is important to point out that PARP inhibitors are only approved in combination with chemotherapy in breast cancer. Thus, identifying mechanisms leading to resistance are important and further approaches that would obviate the toxicity of chemotherapy would improve quality of life for patients. We appreciate the references presented. However, as the reviewer points out these only apply to BRCA1/2 mutated tumors and further they only explain resistance in a subpopulation of patients. Indeed, quoting from the Baxter et al paper
"Despite the identification of reversion mutations in many PARPi-resistant patients (some estimates suggest > 40%), there is a considerable fraction of the PARPi-resistant patient population where the cause of PARPi resistance is not known [13]. Pre-clinical studies have suggested that alterations in a series of other DNA repair proteins cause PARPi resistance by restoring HR without the necessity for genetic reversion of a mutant *BRCA1/2* allele. The causative proteins in these non-reversion mechanisms include 53BP1 [33] (encoded by *TP53BP1*), RIF1 [34], SHLD1 [33, 35, 36, 37], SHLD2 [33, 35, 37, 38], SHLD3 [33, 37], REV7 [39] (encoded by *MAD2L2*), the CST complex [37, 40], PTIP [41], EZH2 [42], DYNLL1 [43, 44], SLFN11 [45], amplification in *TRIP13* [46] or an increase in TIRR [34]. Thus far, extensive evidence for genetic changes in the genes encoding these proteins in clinical PARPi resistance is limited, although *TP53BP1* mutation has been reported in a case of acquired resistance [47]. This may be due, at least in part, to the absence of these genes on common ctDNA sequencing panels used to profile liquid biopsies in patients with drug resistance."
To provide greater clarity for the rationale for the studies of PARP inhibitor resistance in breast cancer, we have revised the introductory paragraph:
"Poly (ADP-ribose) polymerase (PARP) inhibitors (PARPi) have been extensively explored in the laboratory and the clinic based on synthetic lethality with defects in homologous recombination (HR) mediated by mutations in BRCA1/2 and other aberrations in the HR pathway. Although PARPi in combination with chemotherapy have been approved for management of BRCA-associated metastatic triple-negative breast cancer (TNBC) [12, 13], benefits are not durable with almost universal recurrence. The mechanisms underlying PARPi resistance have been explored extensively and include BRCA1/2 reversion mutations, hypomorphic BRCA1/2 alleles, loss of the shieldin complex and activation of pro-survival pathways such as the MAPK and PI3K pathways [14-17]. However, taken together, the identified mechanisms can only explain PARPi resistance in a subset of patients [18]. The most interesting of the

resistance mechanisms is the acquisition of reversion mutations that reconstitute BRCA1/2 functions. However, a recent analysis of over 7000 BRCA1/2 mutant tumors indicates that reversion mutations are relatively uncommon, occurring in only approximately 1% of tumors with BRCA1/2 mutations [19]. In terms of extending the utility of PARPi to a wider population of TNBC patients, multiple preclinical and clinical studies have demonstrated that PARPi combinations are active in patients whose do not have BRCA1/2 mutations or other aberrations in the HR pathway providing an opportunity to provide benefit patients whose tumors do not have aberrations in BRCA1/2 [20-22]. Thus, there is a high priority for understanding resistance mechanisms to PARPi in tumors with and without BRCA1/2 mutations or aberrations in the HR pathway and identifying and implementing novel combinations therapies that are effective across mutational subtypes and that prevent the emergence of resistance or abrogate PARPi resistance once it occurs [23, 24]."

Additional justification is provided below.

PARP inhibitors block BER and inducing replication fork collapse in multiple preclinical breast cancer models. This does result in dsDNA breaks that are synergistic with HR deficiency. However, these dsDNA breaks also induce other responses that can be capitalized on such as the activation of STING responses with subsequent interferon production and immune activation. This results in synergy to a number of different combination therapies in multiple model systema. Furthermore, combination therapy with PARP inhibitors has demonstrated significant activity in breast cancer patients whose tumors do not have BRCA1/2 mutations including a meta-analysis (PMID: 34143979, PMID: 33663560, PMID: 34667258, PMID: 34518313, PMID: 33541181). Our group presented results of a PARPi combinations with ICB at the recent AACR meeting demonstrating a median PFS of 7.9 months and OS of 14.1 months in heavily pretreated BRCA1/2 wild type TNBC. This included a 27% ORR and a 60% CBR. The expected PFS with physician's choice is under 4 months. Thus, PARP inhibitors are active in triple negative breast cancer in patients whose tumors do not have BRCA1/2 mutations. As PARP inhibitor combinations are being evaluated in TNBC in patients whose tumors do not have BRCA1/2 mutations, it is important to understand sensitivity and resistance in this population of patients. One of the goals of this manuscript is to identify combinations with PARPi that would benefit BRCA1/2 wild type TNBC, which is by far the most common form of TNBC. Indeed, this study did identify a highly effective combination therapy: PARPi and C5ar1 inhibition that is effective in a PARP resistant BRCA1/2 wild type TNBC model providing a potential route forward to explore combination therapy in BRCA1/2 wild type TNBC.

b. The team suggests that TNBC patients who have a worse response to ICB have higher expression of C5aR1 however this is disconnected from their main findings that C5aR1+ macrophages are driving PARPi resistance.

This data with TNBC patients is designed to determine whether the effects of C5aR1 macrophages are generalizable beyond PARPi resistance. This data clearly indicates that C5aR1 macrophages may play a role in resistance to multiple therapeutic agents including ICB. Further, we and others have demonstrated that in BRCA1/2 mutant and wild type models that PARPi induce a STING response and engage the immune system and further that immune system engagement is a major contributor to the response to PARPi. Thus, it was appropriate to determine whether $C5aR1^{+}$ macrophages that mediate resistance to PARPi could affect response to ICB. We have clarified the rationale in the text.

c. How are the cancer cells dying in response to the drug if they are not undergoing synthetic lethality?

Synthetic lethality is only one mechanism of action of drugs. Most drugs do not mediate their activity in tumors through synthetic lethality. Indeed, PARP inhibitors as noted above have activity independent of synthetic lethality with BRCA1/2 mutations in many model systems. PARP trapping is just one example where lethality is not dependent on BRCA1/2 mutation status.

2. A major weakness and detrimental limitation to the study as a whole was the generalized used of M1 and M2, which are not biological states of a macrophage in a tumor. There is very poor macrophage biology described in this paper. Since the focus is entirely on tumor macrophages the authors need to address the complexity and heterogenous nature of tumor macrophages. The complexities of tumor macrophages have been studied extensively and this work should incorporate the heterogeneity of these cells. The M1/M2 dichotomy is outdated when describing macrophages in tissues. M1 and M2 definitions are macrophages cultured ex vivo in either LPS/IFNg

(M1) or some combination of IL-4/10/13 (M2). Macrophages in a tumor do not represent either M1 or M2, but likely a mix of both states. Using the terms M1/M2 throughout this paper seriously delays progress in this field and renders the interpretation of the data meaningless.

The terms M1 and M2 macrophages are used in two different manners. One is based on the specific markers used to define the subset in in vitro assays in mouse model systems. This, as the reviewer has indicated has turned out not to fit well into in vivo data and further human data. Expression of RNAs and surface markers do not define function well as macrophages and macrophage polarization are frequently plastic. The other use of the terminology is based on the concept that there is a subset of macrophages that have a dominantly anti-tumor (M1) phenotype and a population that has a dominantly pro-tumor (M2) phenotype. Indeed, in 2022 and 2023, there are more than 500 papers that use the term M2 macrophages reflecting the later concept that there is an M2 phenotype and that in general it is pro-tumor and that there is a M1 phenotype and that it is anti-tumor. However, based on recommendations in a recent review article [25], we have used the terms M1-like and M2-like throughout the manuscript.

We agree with the plasticity of the M1 and M2 phenotype particularly in vivo. Indeed, we present a trajectory analysis that demonstrates that the phenotypes are plastic and that cells do move from one phenotype to another. In the revised paper, we changed the term of M1/M2 into M1-like/M2-like macrophages to specify the difference of our M1/M2 macrophages with the typical classical ones. Further, we do characterize different macrophage subsets that are not the "classical" M1 and M2 macrophages in the manuscript with a focus on a C5aR1 subset of macrophages that are not those that have been extensively characterized previously.

In the introduction to the manuscript, we have clarified the concerns about the use of M1-like and M2-like macrophages and indicate how the terminology is being used in this manuscript (see below). We have also clarified in each Figure, how we are defining M1-like and M2-like macrophage phenotypes to provide clarity. We have also revised the text to indicate what markers were used to define the M1-like and the M2-like phenotype in the manuscript and in the tumor.

"Macrophages, innate immune cells derived from the myeloid lineage, exhibit remarkable epigenomic and transcriptomic plasticity driven, in part, by changes in the microenvironment [26]. Tumor associated macrophages (TAMs) can contribute to tumor clearance or protection based on their polarization into macrophages that mediate anti-tumor or pro-tumor effects, respectively [27]. TAMs have been classified as M1 and M2, respectively, based on makers defined *in vitro* for murine macrophages. While the phenotypic characterization of TAMs as pro-inflammation and pro-tumor has been validated in multiple systems, the markers that were originally used to define murine M1 and M2 macrophages in vitro have not been consistently observed in vivo in murine models or in human tumors. For ease of presentation, we will use the terminology M1-like and M2-like to refer to TAMs that have characteristics associated with antitumor and protumor macrophages, respectively. We realize that this split is simplistic and that there are clearly additional important macrophage subpopulations and further that plasticity can result in challenges in defining the functional characteristics of specific macrophage subpopulations. Thus, to improve clarity, the process used to define the M1-like and M2-like phenotype of particular TAM populations will be indicated throughout the text. TAMs, particularly M1-like macrophages, can also serve as a bridge between innate and adaptive immunity through antigen presentation associated with elevated MHC-II gene expression compared to other TAM subtypes [28]. M1-like and M2-like TAMs mediate many of their pro and anti-tumor activities through production of mutually exclusive sets of cytokines. M1-like TAMs express T_H1 response-inducing cytokines such as IL12, IL1β and TNFα while M2-like TAMs express IL10, CCL8 and CCL22 that are tumor promoting [29]."

a. Marker and functional characterizations of macrophage populations need to be used. There are several clusters of macrophages in the UMAPs, which should be characterized by marker expression.

We have added Figure S1F that demonstrates the RNAs that are expressed in the different populations of macrophages in Figure 1B.

b. In S2A authors show use CSF1R, CD2026 or C5AR1 to define a "M2" population but state throughout the text that CSf1R is both M1 and M2. There is so much confusion in this manuscript regarding what macrophages are

being called and referred to that interpretation is very difficult. Authors need to call populations of macrophages as unique subsets and call them by their marker expression and not “M1/M2”.

Throughout the manuscript and in the Figure legends, we have described the approach used to define the different macrophage subsets. This is now described in greater detail in the methods.

In S2A, we did not use CSF1R, CD2026 or C5aR1 to define the M2 population. Rather the work flow indicates the gating strategies to define the macrophage population under consideration. The first step was to define the total macrophage population by CD11b and F4/80 dual positive staining. These were divided into M1-like and M2-like based on staining for MHCII and CD206, respectively. We then arrayed C5aR1 and CSF1R staining on the macrophage population to show what populations were positive for C5aR1 and CSF1R. We did not define the M1-like or M2-like populations based on C5aR1 and CSF1R. We have now edited the text and the Figure legend to clarify the studies presented in S2A.

3. Several comparisons between CSF1R and C5aR1 are made throughout the manuscript, likely due to a previously published paper showing that CSF1R+ macrophages contribute to PARPi resistance (Mehta et al. Nat. Can 2021). There are too much unclear, unsubstantiated comparisons, ie claims made without statistics. It’s not clear why there needs to be a focus on the comparison. The comparison between CSF1R and C5aR1 causes confusion
a. The data shown here indicate that Ola increases both CSF1R+ macrophages and C5aR1+ macrophages after PARPi. These populations tend to follow a similar trend (Fig. 2F-G). Are these populations overlapping? What is the extent of double positive cells (CSF1R+C5aR1+)?

In order to focus the manuscript on the role of C5aR1 positive macrophages in resistance to PARPi, we have greatly decreased the comparison between Csf1R and C5aR1 positive macrophages throughout the manuscript. We have retained some comparisons such as described below to answer specific concerns raised by the reviewer.

The manuscript is focused on the role of C5aR1 positive macrophages in resistance to PARP inhibitors and the potential for targeting this population to increase effects of PARPi through reversing PARPi resistance. The previous paper demonstrating a role for CSF1R macrophages in PARPi resistance was indeed a trigger for comparison. Further as CSF1R targeting has been used both clinical and preclinically in terms of therapy resistance, and there is a potential for targeting of C5aR1, this is an important consideration.

We have carefully reviewed all comparisons between CSF1R and C5ar1 positive macrophages in the manuscript and have clarified the data supporting each comparison in the text. We have revised Fig 2F-G based on requests from other reviewers and have clarified the effects of olaparib on CSF1R and C5aR1 macrophages.

We have reanalyzed our single cell RNAseq data from Figure 1B and find that cells that have high levels of C5aR1 and do not express Csf1r exhibit a broader spectrum and a higher level of pro-tumor genes and the lowest level of MHCII genes. In contrast cells that have high levels of Csf1r and do not express C5aR1 exhibit a broader spectrum and a higher level of anti-tumor and MHCII genes. The population of macrophages that express high levels of both Csf1r and C5aR1 exhibit both pro- and anti- tumor makers with moderate levels of MHCII genes. The data is consistent with the C5aR1 being dominant M2-like and that the macrophages that express both Csf1r and C5aR1 potentially being a plastic population with both M1-like and M2-like characteristics.

Response to Reviewers Figure 8: Canonical M1 and M2 macrophage marker expression of each subtype of macrophages.

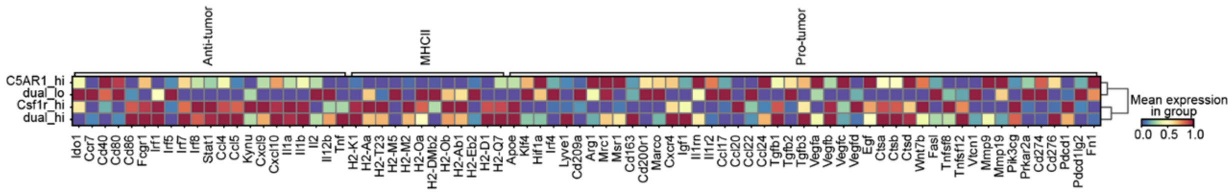

We thus compared the ratio of MHCII^{hi} representing M1-like macrophages with C5ar1^{hi} and Csf1r^{hi} macrophages and showed that the ratio between MHCII^{hi}/C5ar1^{hi} but not MHCII^{hi}/Csfr1^{hi} was significantly enriched in the T22 (PARPi sensitive) but not in the T127 (PARPi resistant) model and in the co-transplantation model (new Figure

S3F). By comparison with canonical macrophage markers, the C5aR1 positive cells express high levels of the majority but not all “pro-tumor” macrophage markers and a subset of the “anti-tumor” markers (new Figure S3G). In contrast, by comparison with canonical macrophage markers, the Csfr1 positive cells express high levels of the majority but not all “anti-tumor” macrophage markers and a subset of the “pro-tumor” markers (new Figure S3G). Further, it appears that the C5aR1 and Csf1r populations are different based on the mutually exclusive expression of high levels of the classical macrophage markers. For example, C5aR1_hi cells express high levels of Il1rn, Cd274, Cxcr1, Cxcr2 pro-tumor macrophage markers with Csf1r_hi cells expressing much lower levels.

Response to Reviewers Figure 9: (F) Bar plot showing relative numbers of indicated cell ratio of each model with and without olaparib in scRNAseq datasets. Upper panel: MHCII^{hi}/C5ar1^{hi}. Lower panel: MHCII^{hi}/Csf1r^{hi}. (G) Canonical M1 and M2 macrophage marker expression of each subtype of macrophages. MHCII^{hi} versus C5ar1^{hi}. Lower panel: MHCII^{hi} versus Csf1r^{hi}.

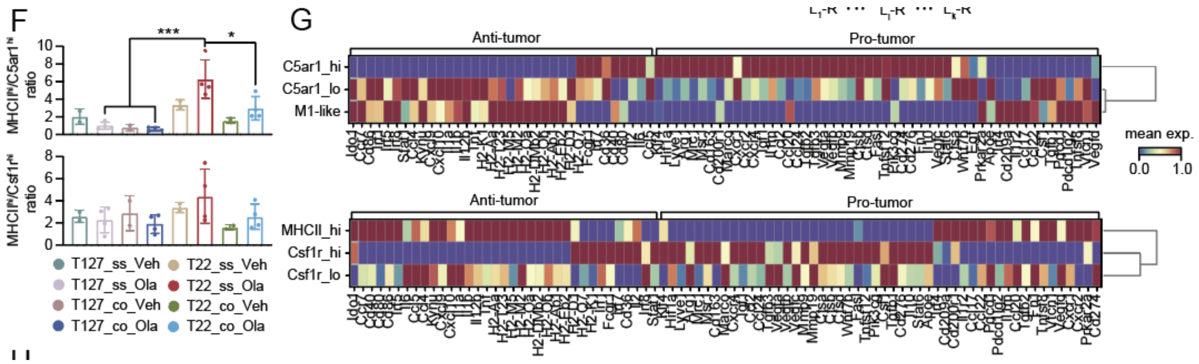

b. Line 427-428; CSF1R appears to be associated with M1 and M2; as does C5ar1 from S4A.

In Figure 1, the initial calling of M1-like and M2-like was done by scPred [1] based on training with a publicly available ICB treated human cohort [2]. We have provided detailed characterization of the Csf1R and C5aR1 positive macrophages as described above in the new Figure S1F and S1G. Thus, as indicated above, neither the Csf1R and C5aR1 positive macrophages exclusively express canonical markers associated with M1 or M2 phenotypes but rather C5ar1 positive markers dominantly express M2-like and Csf1r dominantly express M1-like markers. Further is demonstrates that the two populations are likely mutually exclusive.

c. The authors claim that CSF1R “may contribute to M1 polarization” but there is no real data to show this. Just speculation. The data shown suggests that CSF1R is expressed in the same population of macrophages as C5aR1(high) (CSF1R and C5ar1 is shown in both M1 and M2 populations in Fig 1D; Fig 6G and 6J CSF1R is highest in the CfaR1 high population). Since it is a fairly well published and accepted that CSF1R+ macrophages are associated with a suppressive phenotype, the authors would need to back up their claims with solid data, functional studies and statistics to show that CSF1R is more “M1-like”. Otherwise the authors need to not over state/ over interpret their data. This is a very confusing point they try to make, and their data does not support the claim.
We have removed this concept from the manuscript as suggested.

d. Additionally, the authors show that C5aR1 is expressed in both “Macro-M1” and “Macro-M1” (and Macro-others and Macro-MKI67), making it a pan macrophage marker. It is not clear why they try to suggest that C5aR1 is only on their M2s and CSF1R is on both of their M1s and M2s. Subclustering the macrophage populations and calling them by marker expression and function would potentially provide clarity to the heterogeneity.
We have revised the manuscript to more carefully represent the expression of C5aR1. The heatmap approach in Figure 1E (current Figure 1E) does not provide a clear definition of the expression of C5ar1 and Csf1r on the different populations. We have now added Figure S3G that demonstrates which M1 and M2 associated markers are expressed on the C5aR1 positive macrophages. Throughout the manuscript we now carefully define how the different macrophage populations were defined in the text and in the Figure legends.

Other concerns:
1. The single cell data/flow cytometry in Fig 1-2 has several concerns.

a. Only 2 vehicle mice were used. Statistics cannot be run with an n of 2. Authors must include additional mice in order to run statistical analysis.
We have revised Figure 1-2 and show comparisons where at least four mice were assessed. Although we cannot provide the statistical analysis compared to vehicle, the comparisons presented are statistically significant and validate the concepts related to PARPi resistance in the different models as presented in the text.

b. In Figure 2A it appears there are more Macro-M2 in both the T127 and T22 tumors compared to their "M1". The "M2" do not appear to change after treatment. The abstract reads as though there are more M1 macrophages in the tumors. This is not true. The abstract needs to be revised to reflect the baseline infiltration of macrophages as there are actually more M2's in the tumor before and after treatment.
The text causes confusion and is misleading.

Thank you. We have clarified this as suggested in the abstract as suggested: "Our data indicate that the PARPi-sensitive tumor model compared to PARPi-resistant tumor model has a higher ratio of M1-like pro-inflammatory/M2-like pro-tumor macrophages with the M1-like/M2-like ratio being increased by PARPi."

c. It is not clear how the authors are defining "M1" and "M2" by flow cytometry in Fig. 2B (and throughout, this needs to be clearly defined by markers). Is it the use of S2A or S2C? They show different marker uses for "M2".

We have now clarified the Figure legends throughout and have provided details for how each population was defined in Figure S2A and S2C.

d. *Legend for S2A is wrong related to which Figures the flow corresponds to.
Thank you. We have now corrected the legend for Figure S2A as requested

e. In 2B the analysis of macrophage populations out of total cells should be performed and shown to indicate total numbers of these cells (not the proportion out of macrophages, which is what is currently shown).
As requested, this has been inserted as new Figure 2B.

f. Authors state in 335-337 that T22 had fewer macrophages, but that is not what is shown. Only the proportion out of macrophages is shown. Please revise to reflect what is shown and include the data showing numbers of macrophages out of total cells (as previous comment indicates).
We have now clarified the text as requested "f flow cytometry, potentially due to greater sensitivity and the markers assessed, demonstrated that T22 had fewer M2-like ($MHCII^{lo}/CD206^{hi}$) macrophages than T127 and that olaparib treatment in the single strain model decreased the number of M2-like macrophages." And percentage of total macrophage has now been presented in Figure 2B.

2. Define what "altered functionality" means in line 445.
We have now defined what we mean as "this is associated with up-regulation of phagocytosis and cytokine production transcriptomes in the M1-like macrophages in T22 as well as increased communication between M1-like macrophages and naïve T cells and NK cells in T22 but not T127".

3. Fig 4 C: does not line up with previously shown data including that shown in Fig 2B comparing veh and ola. Fig 4C shows significant increase in their "m2" macrophages after Ola in T127s and no change in T22s; whereas Fig 2B shows no change in "m2" in T127 and decrease in m2 in T22. This discrepancy needs to be explained.
Thank you. We have now clarified the differences between Figure 2B and Figure 4C in the text. Figure 2B includes single strain models (T22 or T127 on both sides) as well as compared to the co-transplantation model ie different tumor on both sides. Figure 4C only includes the co-transplantation model and the data in Figure 4C refers to the T22 and T127 tumors in the co-transplantation models. This leads to the apparent discrepancy and is explained by the co-transplantation of T22 and T127 versus only a single tumor model. We have now clarified this in the text as well as in the Figure legend.

To compare the consistency of T22_co and T127_co between Figure 2 and Figure 4, we have now re-processed the flow data as requested by other reviewers and removed what were likely dead cells. After re-processing, there is no significant increase in the number of M2-like macrophages after olaparib treatment in in the T127_co or T22_co.

Similar to Figure 2B there is a slight increase in the number of M2-like macrophages in T127_co_ola compared with T127_co_veh in Figure 4C.

To compare differences between T127_co and T127_ss after olaparib, we also found that M1-like/M2-like decreased when T127 was co-transplanted with T22 and treated with olaparib in both Figure 2 and Figure 4. Although the changes were statistically significant the changes were minor when compared to the changes in the ratio in the T22 tumors.

4. Data shown in S6C is not interpretable because the Ola did not work the same as in Fig 3B. S6C shows no effect of Ola on either the T22 or T127 however in the experiment with the PMX53 compound there was a reduction in tumor burden with Ola monotherapy shown in Fig 3B.
The growth curves shown in Figure 3B is from the single strain models while the growth curves shown in the Figure S6 were all from the co-transplanted models. As we have shown and discussed in Figure 1, when T22 was transplanted alone in a mouse, T22 was sensitive to olaparib but when co-transplanted with T127, the sensitivity of T22 to olaparib was attenuated by the T127 tumor on the opposite side. We have now clarified in the text and in the Figure legend that the tumors in Figure S6 were from co-transplantation models.

5. It's not clear how or why C5ar1 and CSF1r would be inversely correlated in Fig 5C when Fig 1D (and other Figure) show that the macrophage populations express both receptors.
As indicated above, while C5ar1 and Csf1r are expressed on both M1-like and M2-like populations, we demonstrate in the new Figure S3G that they are expressed on mutually exclusive populations based on the expression of canonical M1-like and M2-like markers. The fact that the two populations do not exclusively express M1-like or M2-like markers as demonstrated in Figure S3G results in the indication in Figure 1D that C5ar1 and Csf1r are expressed on both M1-like and M2-like macrophages when these are defined by the aggregate marker set used to train scPred (see Methods).

6. Supl Fig 7H: csf1 and il34 are ligands for csf1r. Is CSF1R also upregulated in this assay? This experiment seems out of context and the connection to C5aR1 is confusing. I suppose IL34 is making the macrophages more M2-like and therefore express C5aR1, but wouldn't these macrophages also express CSF1R?
As indicated by the reviewer Csf1 and Il34 are ligands for Csf1R and would induce macrophage proliferation. Interestingly as the reviewer indicates this results in an increase C5aR1 positive macrophages. This Figure is used to explore the regulation of C5aR1 expression. As recommended by the reviewer, we have decreased the comparisons between Csf1R and C5aR1 where possible and we have removed the expression of Csf1R from the Figure as it is not required to understand mechanisms regulating C5aR1.

7. Authors need to support the claim that C5aR1 macrophages are suppressive through ex vivo functional studies and genetic manipulation.

We have now added a new Figure 5 with in vitro culture of T127 (resistant to PARPi) with C5aR1 positive and negative macrophages purified from naïve mice and then subsequently with CD8 positive T cells from naïve mice. The data show that T127 isolated from olaparib treated mice educated the C5aR1 positive macrophages and that they subsequently decreased both granzyme B and perforin production by the CD8 positive T cells. This was reversed in part by inhibition of C5aR1 with PMX53. This study was described in detail in response to reviewer #3 and is inserted below.
We performed in vitro co-culture assays of T127 tumor cells from mice treated with and without olaparib with C5aR1+/- macrophages and CD8 T cells to validate the observations in scRNA-seq data. The new results have been added to the text in the new Figure 5. To investigate whether C5aR1 inhibited macrophages contributes to anti-tumor activity by evoking T cell cyto-toxicity function, we first isolated T127 tumor cells (as described in methods) from vehicle or olaparib treated mice as described in Figure 1, then incubated the T127 tumor cells with CD11b+C5aR1- and CD11b+C5aR1+ macrophages isolated (as described in methods) from tumor naïve mouse splenocytes in a boydon chamber in the presence and absence of PMX53 (30nM) for 24h. After removal of PMX53, we co-cultured the macrophages with CD8+ T cells isolated from tumor naïve mouse splenocytes. We found that C5aR1- and C5aR1+ macrophages incubated with T127 from vehicle treated mice did not differentially affect subsequent CD8 activation except for a modest decrease in perforin levels in cells cultured with C5aR1+ macrophages (new Figure 5A in manuscript presented below). The C5aR1 inhibitor PMX53 had only modest effects

(New Figure 5A). In contrast, when T127 tumor cells from mice treated with olaparib were incubated with C5aR1+ macrophages, there was a marked decrease in granzyme B and perforin in the CD8 T cells that was partially reversed by incubation with PMX53 (New Figure 5B). For reasons that are unknown, the CD5aR1 macrophages incubated with T127 from olaparib treated mice showed a marked increase in IFN-γ expression that was further enhanced by PMX53 (New Figure 5B).

Response to Reviewers Figure 10: C5aR1 antagonist treated macrophages activate T cell cytotoxicity ex vivo.

(A) 1×10^6 T127 tumor cells were isolated from vehicle control treated T127 tumor bearing mice as described in Figure 1 and cultured with 5×10^5 $CD45^+$ $CD11b^+C5aR1^{-/+}$ macrophages isolated from the spleen of tumor naïve mice in a Boydon chamber for 24 hours with the $CD45^+$ $CD11b^+C5aR1^{-/+}$ macrophages cultured with and without PMX53 as indicated. One day later, the $CD45^+CD11b^+C5aR1^{-/+}$ macrophages were isolated from the Boydon chamber and then co-cultured with 5×10^5 CD8 T cells isolated from the spleen of tumor naïve mice for 4 days. Fraction of $GZMB^+$, $PRF1^+$, IFN-γ^+ $CD8^+$ T cells in each indicated group were assessed by flow cytometry with antiCD8 and antibodies to granzyme B, perforin and interferon gamma respectively. n=3, * $p < 0.05$, ** $P < 0.01$, *** $p < 0.001$, **** $p < 0.0001$.

(B) Figure 5B was identical to Figure 5B except that T127 cells were isolated from mice treated with olaparib as described in Figure 1.

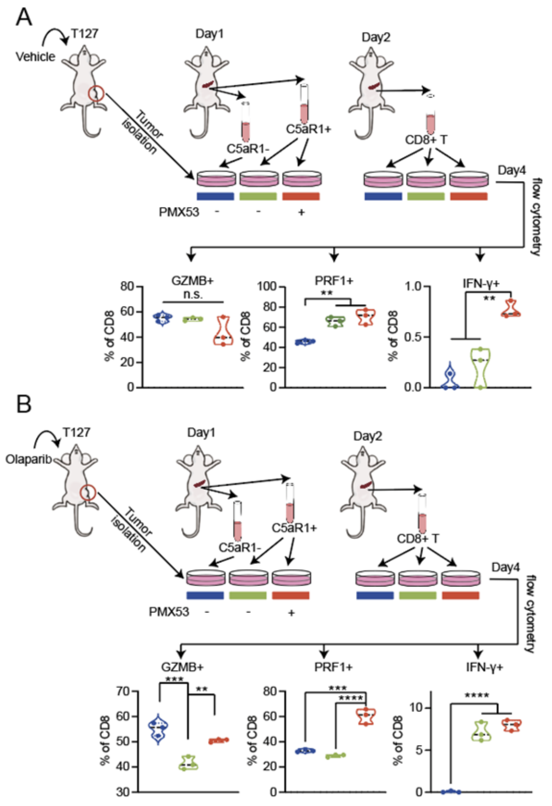

Other minor concerns:

1. Figure 1B needs stats with average tumor volume curves. The text compares T22 and T127 but they are graphed on separate graphs. Conclusions are hard to make without proper statistical analysis. It is not clear what the gray colors of the circles are meant to indicate in Fig 1A.

Figure 1B has been revised and split into Figure 1B and 1C for clarity. Stats have now been included.

2. S1A-C is difficult to see; consider darker colors (S4C looks much better).

We have revised the colors as suggested

3. Fig 3E: show the M1/M2 ratio below the graph to indicate the ratio of increased M2s.

Figure 3E: The last panel now shows the ratio of M1-like/M2 like macrophages

4. Figure 5 is blurry.

We have replaced Figure 5 with a higher resolution Figure as suggested.

5. Text indicates an “other” macrophage cluster in Fig 6B-C, but is not shown in the Figure.

We have now corrected the text to relate to Figure 6B-C

Reviewer #3 (Remarks to the Author)

In this manuscript, Li et al. report a new mechanism of resistance to the PARP1/2 inhibitor olaparib. The authors show that C5aR1 positive M2 macrophages are mediating both local and distant therapy resistance to olaparib in a syngeneic murine breast cancer model. Accordingly, the authors proposed that C5aR1 inhibition may be a good therapeutic strategy to sensitize tumour to olaparib.

Major comments

1.- In co-transplantation models, both T22 and T127 tumours grow faster than when implanted individually, regardless of whether they are treated with olaparib or vehicle. What mechanisms may be involved?

Thank you for this important comment. We noticed this also. As indicated in revised Figure 1D, this is primarily due to less heterogeneity in growth of the tumors. We have not explored this question in this manuscript as it does not impact the outcomes of the studies herein related to the transfer of resistance to PARP inhibitors from T127 to T22 and the resultant improved understanding of PARP inhibitor resistance. We will explore it in a future manuscript.

2.- Olaparib, which inhibits both PARP1 and PARP2, is used throughout the work. A selective PARP1 inhibitor is now available (AZ5305; Illuzzi et al., 2022), clinical trials are ongoing, and is commercially available. It would be interesting, using this new inhibitor, to determine the specific role of PARP1 and to assess whether the proposed therapeutic combination for olaparib can be extended to the new generation of selective PARP1 inhibitors.

Thank you for this suggestion. As indicated below have assessed the combination of AZD5305 and PMX53 in KPCA syngeneic mouse models derived from the precursor of ovarian cancer, the fallopian tube epithelium [7]. As expected, mono-therapy with olaparib, AZD5305 or PMX53 only induced modest tumor growth inhibition. However, significant growth control was obtained by the combination treatment with either olaparib or AZD5305 and the PMX53 C5aR1 inhibitor. Olaparib and AZD5305 in combination with PMX53 demonstrated similar activity. New Figure has been added as Figure S6D.

Response to Reviewers Figure 3: Tumor growth curve of KPCA mouse model treated with control, olaparib, AZD5305 (0.4mg/kg, o.g., daily), PMX53, the combination of PMX53 and olaparib or the combination of PMX53 and AZD5305 for 28 days. The day 29 volumes were derived by measurement of dissected tumors which is more accurate that measurement of masses in the mice.

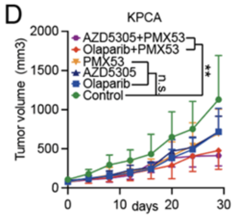

In addition, we tested AZD5305 in a co-culture study that demonstrates that C5aR1 macrophages incubated with AZD5305 treated T127 would suppress CD8 T cell activation. This inhibitory effect can be rescued by PMX53. As indicated in Figure 2 below (new Figure S5L in the paper), incubation of T127 tumor cells in vitro with AZD5305 and then subsequent incubation of the T127 cells with C5aR1 positive macrophages results in a marked decrease in granzyme B and interferon production and a more modest decrease in perforin production. The effects of the AZD5305 incubated T127 on the ability of C5aR1 positive macrophages to alter T cell activation were reversed by inhibition of C5aR1 with PMX53.

Response to Reviewers Figure 4: Work flow of co-culture assay: T127 tumor cells were purified from mice and then treated with vehicle or AZD5305 (5nM) for 4 days in culture. CD11b+C5aR1-/+ macrophages isolated from tumor naive mice were added on day 4. Cells were isolated and then cultured with T cells from tumor naïve mice ex vivo for 2 days and cellls assessed for activation markers by flow cytometry (see Methods for details). 1×106 T127 tumor cells were treated with or without AZD5305 (5nM) in culture for 4 days. They were co-cultured for 1 additional day with 5×105 CD11b+C5aR1-/+ macrophages isolated from CD11b+ positive splenocytes from tumor naïve mice with or without PMX53 (40nM). On day 5, CD11b+C5aR1-/+ macrophages were isolated and then co-cultured with 5×105 CD8 T cells from tumor

naïve mice. Fraction of GZMB+, PRF1+, IFN-γ+ cells of CD8+ T cells in each indicated group from left assessed by flow cytometry. n=3, * p < 0.05, ** P < 0.01, *** p < 0.001, **** p < 0.0001.

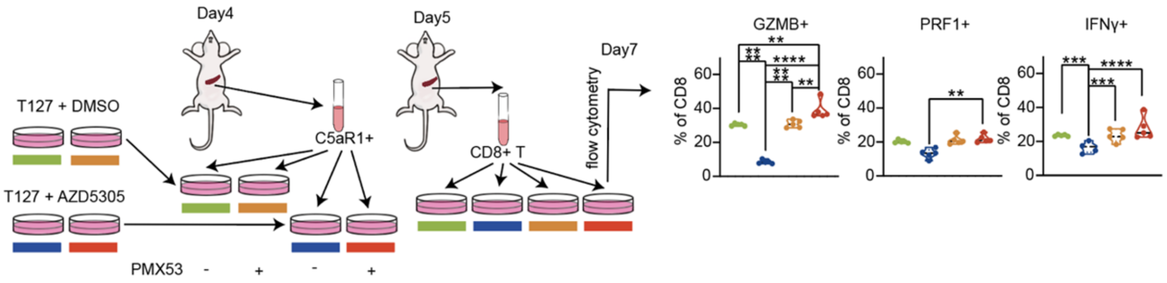

3.- In the T127 model, in Figure 1B, a large variability is observed, especially in response to olaparib treatment, with some mice being "sensitive" and others more resistant. How can this large experimental heterogeneity be explained? Has it been taken into account in any way in the following analyses?

As noted above, the T22 and T127 models are heterogeneous including in growth rates in vivo. This arises from this being a transplantation model that has not been selected for growth on plastic. These models were selected in part due to the heterogeneity in growth rates as these parallels what is seen with human tumors as indicated by variable growth rates of PDXs. The apparent differential response to olaparib indicated by the reviewer is not a difference in sensitivity to olaparib but rather heterogeneity in the growth rate. Indeed, there is no different in the growth rates for the T127 tumor in vehicle or olaparib treated tumors in Figure 1B. We have revised Figure 1B to make this clearer.

4.- Data on the effect of olaparib on macrophage function have been estimated based on scRNAseq results. Similarly, the interaction between cell populations has been estimated based on scRNAseq data. It would be interesting to validate these predictions.

Thank you for the suggestions. We performed in vitro co-culture assays of T127 tumor cells from mice treated with and without olaparib with C5aR1+/- macrophages and CD8 T cells to validate the observations in scRNA-seq data. The new results have been added to the text in the new Figure 5. To investigate whether C5aR1 inhibited macrophages contributes to anti-tumor activity by evoking T cell cyto-toxicity function, we first isolated T127 tumor cells (as described in methods) from vehicle or olaparib treated mice as described in Figure 1, then incubated the T127 tumor cells with CD11b+C5aR1- and CD11b+C5aR1+ macrophages isolated (as described in methods) from tumor naïve mouse splenocytes in a boydon chamber in the presence and absence of PMX53 (40nM) for 24h. After removal of PMX53, we co-cultured the macrophages with CD8+ T cells isolated from tumor naïve mouse splenocytes. We found that C5aR1- and C5aR1+ macrophages incubated with T127 from vehicle treated mice did not differentially affect subsequent CD8 activation except for a modest decrease in perforin levels in cells cultured with C5aR1+ macrophages (new Figure 5A in manuscript presented below). The C5aR1 inhibitor PMX53 had only modest effects (New Figure 5A). In contrast, when T127 tumor cells from mice treated with olaparib were incubated with C5aR1+ macrophages, there was a marked decrease in granzyme B and perforin in the CD8 T cells that was partially reversed by incubation with PMX53 (New Figure 5B). For reasons that are unknown, the CD5aR1 macrophages incubated with T127 from olaparib treated mice showed a marked increase in IFN-γ expression that was further enhanced by PMX53 (New Figure 5B).

Response to Reviewers Figure 5: C5aR1 antagonist treated macrophages activate T cell cytotoxicity ex vivo. (A) 1×10^6 T127 tumor cells were isolated from vehicle control treated T127 tumor bearing mice as described in Figure 1 and cultured with 5×10^5 $CD45^+$ $CD11b^+C5aR1^{-/+}$ macrophages isolated from the spleen of tumor naïve mice in a Boydon chamber for 24 hours with the $CD45^+$ $CD11b^+C5aR1^{-/+}$ macrophages cultured with and without PMX53 as indicated. One day later, the $CD45^+CD11b^+C5aR1^{-/+}$ macrophages were isolated from the Boydon chamber and then co-cultured with 5×10^5 CD8 T cells isolated from the spleen of tumor naïve mice for 4 days. Fraction of $GZMB^+$, $PRF1^+$, IFN-γ^+ $CD8^+$ T cells in each indicated group were assessed by flow cytometry with

antiCD8 and antibodies to granzyme B, perforin and interferon gamma respectively. n=3, * $p < 0.05$, ** $P < 0.01$, *** $p < 0.001$, **** $p < 0.0001$.
(B) Figure 5B was identical to Figure 5B except that T127 cells were isolated from mice treated with olaparib as described in Figure 1.

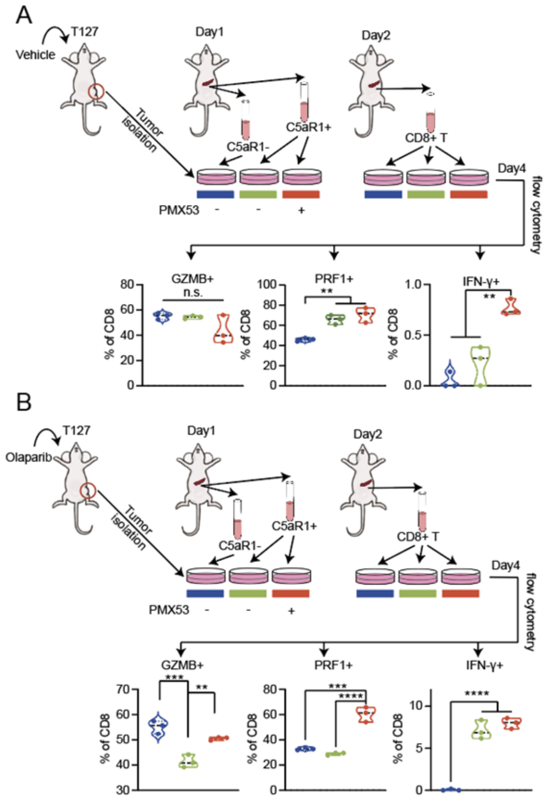

Minor comments
Line 296.- Authors stated “See Figure 1A for a list of samples assessed”. Figure 1A does not show a list of samples assessed for scRNAseq
Thank you for the comment. We have revised the text to read: Individuals samples are presented in Figure S1E (in the updated version).
2.- Figure 1B: T22 tumour growth curves are represented in the upper panel and T127 in the lower panel. However, the legend to the Figure indicates “Tumor growth curve of T127 (upper) and T22 (lower)”.
Thank you for the comment. We have now separated T22 and T127 into Figure 1B and 1C for clarity.
3.- Legend Y-axis of Figure 1B, 3B, 3C, 4A, 4B, 4E, 4F: Tumor instead Tomur.
Apologies for the typo. This has been corrected as suggested.
4.- The legend in Fig. 3B and 3C should be corrected as shown in the Figure.
Apologies for the mismatched figure legends. This has been corrected as suggested.
5.- The definition of the experimental groups in Figure 4F is not clear, and does not coincide with the legend in Figure 4F.
Thank you for the comments. This has been clarified in the Figure and legend as suggested.

Reviewer #4 (Remarks to the Author)

This report utilizes two syngeneic mouse tumors (T22 and T127) to assess the contribution of immune cell populations to PARP inhibitor (PARPi) sensitivity. Consistent with their previous publication, the authors note that T22 is sensitive to PARPi treatment upon transplantation into syngeneic recipient mice, whereas T127 is resistant. The ratio of M2 vs. M1-like macrophage populations also differs between these tumors. In line with published work, abundant M2 macrophages in T127 tumors is coincident with immune suppression and PARPi resistance. The authors analyze immune cells also by scRNAseq in both single and co-transplantation settings. Using such analysis, they suggest C5ar1 as a new receptor on M2 macrophages that may be important for the immune suppressive phenotype observed in such tumors. Finally, they draw upon data from patients that received immune checkpoint inhibitor treatment to assess whether C5ar1is indeed important for immune suppression in these tumors.

This is an interesting study; however, it has many fundamental technical shortcomings that significantly impact the validity of the conclusions drawn from the data. As such, it is currently unsuitable for publication.

Specific points

1. The authors indicate that both T22 (PARPi sensitive) and T127 (PARPi resistant) are both HR proficient due to the fact that they are BRCA1/2 wildtype (line 77-79). They need to provide this data on BRCA1/2 or reference appropriately. Their previous Cancer Research paper referenced here does not contain these data either. In addition, there are many other well-established ways by which cells can acquire HR deficiency independently of BRCA1/2 mutation. At a minimum, to prove that both tumor models are HR proficient, the authors must provide conclusive data on the mutation status of key HR genes, including but not limited to BRCA1/2 as well as another measure of HRD. This may encompass RAD51 repair foci analysis or HRD scoring by other means such as HRdetect. This is essential since the conclusion of the manuscript changes dramatically if there are differences in HR proficiency.

Thank you for this important comment. This is indeed an important point that we have now clarified in the revised manuscript. As indicated in the Science Advances paper which is properly cited now, we have performed whole exome sequencing on T22 and T127. Analysis of 33 genes implicated in HR repair including BRCA1/2, PALB2 and Rad51 failed to detect mutations. Further as indicated in new Figure S1A, both T22 and T127 are positive for Rad51 foci indicative of competent HR.

Response to Reviewers Figure 6: Representative immunofluorescence images of RAD51 foci formation in indicated MDST tumors. Veh = vehicle. Ola = olaparib. Data are representative of three replicates.

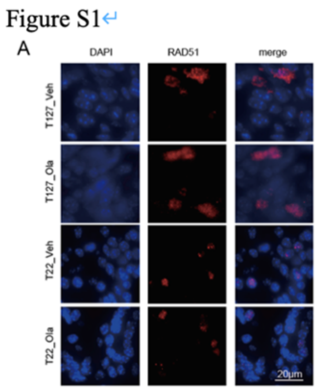

2. The co-transplantation approach is confusing. Clarification is needed here. The authors state that 10 or 14 days after transplantation, mice were assigned to treatment groups based on randomization by tumor size (line 107+108). How exactly and under what rationale was this carried out? This is especially confusing since they also state in the methods section that T22 cells were transplanted 14 days prior to T127 due to big differences in tumor growth rates (line 108). This is very important since throughout the manuscript with or without co-culture, both models seem to follow very similar trajectories upon palpation (Figure 1B, 3B+C for example).

Thank you for this important comment. This is indeed another important point that we have now clarified in the revised manuscript. The T22 model and the T127 model demonstrate different initial growth rates. To accommodate the differences in the time required for the tumors to become established and enter into and exponential growth phase, we introduced the T22 model 2 weeks before the T127 model that was introduced when T22 tumor reaches around 20mm^3. Once the tumors are established, their growth rates are similar (see Figure 1B, 1B and 3C as indicated by the reviewer). Thus, the difference in when the tumors were transplanted were due to differences in when the tumors became established but once established subsequent growth trajectories were similar. Mice were randomized when the size of the T22 and T127 tumors were approximately equal. As requested by the reviewer, we have modified the description of the randomization into "mice were first assigned individual numbers and then assigned to treatment groups (n>5) by online random number generator (http://www.graphpad.com/quickcalcs/randomize1/)."

It is important to note that the T22 and T127 are transplantation models. They are thus much more heterogenous than tissue culture derived cell culture models. Indeed, they we selected based on their heterogeneity of growth that more closely reflects human tumors than xenografts. Indeed, we have presented the data as spider plots to emphasize the heterogeneity in growth of each tumor.

3. Presentation of Figure 1B is not clear. Please present data as dots per time point and condition with appropriate error bars, equivalent to Figure 4A+B for example.

Figure 1B has now been modified. We have retained individual tumor growth curves as "spider plots" to show the heterogeneity of tumor growth and have separated the T22 and T127 growth curves for clarity. Growth curves similar to Figure 4A and 4B have been added as new supplementary Figure S1B as requested.

4. T22 seems to contain only 10-20% tumor cells within the recovered mass with more than 80% immune and somatic cells in some mice (Figure S1B). This is striking and the authors need to elaborate on how this relates to the makeup of tumors in patients or in similar mouse models. The immune cell populations are seemingly not consistent when the authors examine the same models. For example, M2 macrophages are present at the same level in scRNA analysis of T22/T127 in Figure S1B but differ dramatically in Figure S4B+D. These data are also not consistent with the associated flow cytometry data. In addition, the representative gating used for this cytometry (Figure S2, S3, S6) shows that the authors strategy was not appropriate, which may explain this discrepancy. They did not omit very low FSC/SSC particulate (Figure S2A, S6E), which often is found in such samples in the form of debris and these. It is necessary to re-analyze these data and then re-assess their key conclusions regarding immune cell composition together with their existing scRNAseq data.

Thank you for the comment. The tumor size as well as the duration of growth in Figure S1 and S4B were markedly different. The tumors in Figure S1 were grown for a longer time period and had a larger size than the tumors in S4B. These studies were performed to determine differences over time in tumor and immune infiltration in the two models. This suggests that the duration of tumor growth and tumor size influences the degree of infiltration of the tumor by immune and somatic cells.

Thank you for the important suggestion of flow-cytometry re-assessing. As suggested by the reviewer, we have re-assessed the flow cytometry data using the approach recommended and have modified the Figures based on these results. The macrophage subtyping methods are quite different between scRNA-seq M1-like/M2-like classification and the flow M1-like/M2-like classification. The scRNA-seq calling is based on a large number of mRNAs based on scPred trained on human macrophages. The flow cytometry data is based on protein levels with a more limited number of markers. We have added a new Figure S3F that uses the quantification approach suggested by the reviewer. Based on the recommended analysis in the new Figure S3F, the ratio between M1-like/M2-like and the MHC^{hi}/C5aR1^{hi} now falls in the same range as that from the scPred analysis.

5. The authors then draw upon analysis of existing patient data to validate their key findings in TNBC tumors. They determine M2/M1 populations using unconventional markers and importantly, they elect C5ar1 as a marker to delineate M2/M1. This is not appropriate. The data, as presented, is explained by the fact that higher M2 macrophages, independently of C5ar1, correlate with poor survival as expected on the basis of published literature. Under this light, C5ar1 may be simply expressed at a higher level in such cells, without any functional consequence. This needs to be examined.

Thank you for the comment. Yes, it is indeed possible that C5aR1 is simply a surrogate for M2 macrophages that have been associated with a worsened survival. To determine whether this was the case, we have compared outcomes in patients with CSF1R or CD163 positive macrophages with that C5aR1 macrophages to Figure S8. Importantly as indicated in the new Figure S3G, C5aR1 and CSF1R positive macrophages express different sets of canonical macrophage markers. CD163 was included as a classical M2 macrophage marker. Importantly as compared to C5aR1 positive macrophages, the CSF1R and CD163 macrophage populations had much lower predictive power.

6. General importance of C5ar1 in M2/M1 balance and PARPi sensitivity needs to be confirmed using another independent syngeneic mouse model to validate the authors findings.

We have also tested the combination in KPCA syngeneic mouse models derived from the precursor of ovarian cancer, the fallopian tube epithelium. As expected, mono-therapy with two PARPi olaparib and AZD5305 as well as PMX53 only induced modest tumor growth inhibition. However, significant growth control was obtained by the combination treatment with both PARPi and the PMX53 C5aR1 inhibitor. New Figure has been added in Figure S6D.

Response to Reviewers Figure 7: Tumor growth curve of KPCA mouse model treated with control, olaparib, AZD5305 (0.4mg/kg, o.g., daily), PMX53, the combination of PMX53 and olaparib or the combination of PMX53 and AZD5305 for 28 days. The day 29 volumes were derived by measurement of dissected tumors which is more accurate that measurement of masses in the mice.

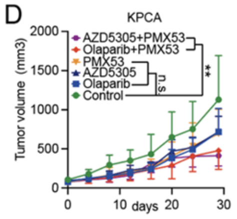

Some additional minor concerns exist:

It would be useful to analyze known receptor ligand interactions as a positive control in their bioinformatic analysis presented in Fig 2, Fig S2+3.

Thank you for the suggestion. We have now added a section on known receptor ligands to the text.
"Known interactions between macrophages and tumors such as Lgals9-Cd45, H2ab1/H2aa-Cd4, Csf1-Csf1r, Cx3cl1-Cx3Cr1, Ccl7-Ccr2 were captured by CellChat."

Figure 2E is in apparent conflict with statements made in line 373+374. Olaparib treatment decreased C5ar1 expression in T22 single transplant tumors. This needs to be acknowledged and discussed in relation to their later findings in Figure 2F+G.

In Figure2E interpretation, we wrote: "Of the receptors, C5ar1 was markedly upregulated by olaparib in both the T127 and T22 co-transplantation models (Figure 2E)." Figure 2E is the total population of M2 macrophages.

We apologize for any confusion and have edited the text as indicated: "Of the receptors, C5ar1 on M2-like macrophages was markedly upregulated by olaparib in both the T127 and T22 co-transplantation models and T127 single strain models but not in T22 single strain models (Figure 2E)."

For Figure 2F+G, it shows the macrophages isolated from the peripheral blood. In both T22 and T127 single strain models, the changes in Figure 2F that represents data from blood are similar with the changes in Figure 2E that represent data from the tumors. In the co-transplantation model, it is important to note that the peripheral blood is from mice with T22 and T127 in the opposite fat pads, therefore the changes of $MHCII^{lo}C5aR1^{hi}$ macrophages might not be the same as either T22 or T127 alone mice but rather is expected to be similar to the average of mice with T22 or T127 tumors. However, as indicated in Figure 2G, similar to Figure 1E, Csf1r was not expressed

preferentially in M2-like macrophages. The difference in the changes in $MHCII^{lo}C5aR1^{hi}$ macrophages and $MHCII^{lo}CSF1R^{hi}$ macrophages indicates that C5aR1 and CSF1R are likely expressed on different macrophage subsets.

As requested, we have now added this concept to the paper.

The significant NK cell populations detected in tumors in Figure S4B do not appear to be present in the same tumor model presented in Figure S1. How is this possible?

Thank you for the comment. The tumor size as well as the duration of growth in Figure S1 and S4B were markedly different. The tumors in Figure S1 were grown for a long time period and had a larger size. We expect that the duration of tumor growth and the size of the tumor altered the NK cell content.

The authors need to confirm that C5ar1 inhibitor works equally well in their T22/T127 tumor models to supplement Figure 4.

Thank you for the suggestion. The comparison of tumor growth inhibition has been added in Figure S6B and demonstrates equal growth inhibition induced by the C5aR1 inhibitor in the presence of olaparib in both tumor models.

References

1. Alquicira-Hernandez, J., et al., *scPred: accurate supervised method for cell-type classification from single-cell RNA-seq data.* Genome Biol, 2019. **20**(1): p. 264.
2. Zhang, Y., et al., *Single-cell analyses reveal key immune cell subsets associated with response to PD-L1 blockade in triple-negative breast cancer.* Cancer Cell, 2021. **39**(12): p. 1578-1593 e8.
3. Azizi, E., et al., *Single-Cell Map of Diverse Immune Phenotypes in the Breast Tumor Microenvironment.* Cell, 2018. **174**(5): p. 1293-1308 e36.
4. Long, Z., et al., *Single-cell multiomics analysis reveals regulatory programs in clear cell renal cell carcinoma.* Cell Discov, 2022. **8**(1): p. 68.
5. Kadomoto, S., K. Izumi, and A. Mizokami, *Macrophage Polarity and Disease Control.* Int J Mol Sci, 2021. **23**(1).
6. Yi Zhang, G.X., Alva Yijia Jiang, Allen Lynch, Zexian Zeng, Chenfei Wang, Wubing Zhang, Jingyu Fan, Jiajinlong Kang, Shengqing Stan Gu, Changxin Wan, Boning Zhang, X. Shirley Liu, Myles Brown, Clifford A Meyer, *MetaTiME: Meta-components of the Tumor Immune Microenvironment.* BioRxiv.
7. Iyer, S., et al., *Genetically Defined Syngeneic Mouse Models of Ovarian Cancer as Tools for the Discovery of Combination Immunotherapy.* Cancer Discov, 2021. **11**(2): p. 384-407.
8. Chen, Z., et al., *Deep exploration of PARP inhibitors in breast cancer: monotherapy and combination therapy.* J Int Med Res, 2021. **49**(2): p. 300060521991019.
9. Labrie, M., et al., *Multiomics analysis of serial PARP inhibitor treated metastatic TNBC inform on rational combination therapies.* NPJ Precis Oncol, 2021. **5**(1): p. 92.
10. Stringer-Reasor, E.M., et al., *An open-label, pilot study of veliparib and lapatinib in patients with metastatic, triple-negative breast cancer.* Breast Cancer Res, 2021. **23**(1): p. 30.
11. Pusztai, L., et al., *Durvalumab with olaparib and paclitaxel for high-risk HER2-negative stage II/III breast cancer: Results from the adaptively randomized I-SPY2 trial.* Cancer Cell, 2021. **39**(7): p. 989-998 e5.
12. Litton, J.K., et al., *Talazoparib in Patients with Advanced Breast Cancer and a Germline BRCA Mutation.* N Engl J Med, 2018. **379**(8): p. 753-763.
13. Robson, M., et al., *Olaparib for Metastatic Breast Cancer in Patients with a Germline BRCA Mutation.* N Engl J Med, 2017. **377**(6): p. 523-533.
14. Sun, C., et al., *Rational combination therapy with PARP and MEK inhibitors capitalizes on therapeutic liabilities in RAS mutant cancers.* Sci Transl Med, 2017. **9**(392).
15. Lord, C.J. and A. Ashworth, *Mechanisms of resistance to therapies targeting BRCA-mutant cancers.* Nat Med, 2013. **19**(11): p. 1381-8.
16. Dev, H., et al., *Shieldin complex promotes DNA end-joining and counters homologous recombination in BRCA1-null cells.* Nat Cell Biol, 2018. **20**(8): p. 954-965.
17. Sun, C., et al., *Systems approach to rational combination therapy: PARP inhibitors.* Biochem Soc Trans, 2020. **48**(3): p. 1101-1108.
18. Baxter, J.S., et al., *Resistance to DNA repair inhibitors in cancer.* Mol Oncol, 2022. **16**(21): p. 3811-3827.

19. Darabi, S., et al., *BRCA1/2 Reversion Mutations in Patients Treated with Poly ADP-Ribose Polymerase (PARP) Inhibitors or Platinum Agents.* Medicina (Kaunas), 2022. **58**(12).
20. Ledermann, J., et al., *Olaparib maintenance therapy in patients with platinum-sensitive relapsed serous ovarian cancer: a preplanned retrospective analysis of outcomes by BRCA status in a randomised phase 2 trial.* Lancet Oncol, 2014. **15**(8): p. 852-61.
21. Poveda, A., et al., *Olaparib maintenance monotherapy in platinum-sensitive relapsed ovarian cancer patients without a germline BRCA1/BRCA2 mutation: OPINION primary analysis.* Gynecol Oncol, 2022. **164**(3): p. 498-504.
22. Shao, F., et al., *Efficacy and safety of PARP inhibitors as the maintenance therapy in ovarian cancer: a meta-analysis of nine randomized controlled trials.* Biosci Rep, 2020. **40**(3).
23. Kaufman, B., et al., *Olaparib monotherapy in patients with advanced cancer and a germline BRCA1/2 mutation.* J Clin Oncol, 2015. **33**(3): p. 244-50.
24. Tutt, A., et al., *Oral poly(ADP-ribose) polymerase inhibitor olaparib in patients with BRCA1 or BRCA2 mutations and advanced breast cancer: a proof-of-concept trial.* Lancet, 2010. **376**(9737): p. 235-44.
25. Monnier, M., et al., *Antitumor strategies targeting macrophages: the importance of considering the differences in differentiation/polarization processes between human and mouse macrophages.* J Immunother Cancer, 2022. **10**(10).
26. Amit, I., D.R. Winter, and S. Jung, *The role of the local environment and epigenetics in shaping macrophage identity and their effect on tissue homeostasis.* Nat Immunol, 2016. **17**(1): p. 18-25.
27. Ma, R.Y., A. Black, and B.Z. Qian, *Macrophage diversity in cancer revisited in the era of single-cell omics.* Trends Immunol, 2022.
28. Biswas, S.K. and A. Mantovani, *Macrophage plasticity and interaction with lymphocyte subsets: cancer as a paradigm.* Nat Immunol, 2010. **11**(10): p. 889-96.
29. Anderson, N.R., et al., *Macrophage-Based Approaches for Cancer Immunotherapy.* Cancer Res, 2021. **81**(5): p. 1201-1208.

REVIEWERS' COMMENTS:

Reviewer #1 (Remarks to the Author):

In general, the manuscript has been improved. The authors have addressed most of my previous comments. However, as I raised previously, the fundamental concerns regarding single-cell analysis persist that need to be addressed.

1. Regarding the clustering and annotation, the authors used scPred to obtain cell subsets from the training of single-cell data from the human. However, several studies have reported that non-conserved immune cell subsets exist between human and mouse models, especially the macrophages. The authors should perform clustering on their data independently. For the relevance of human populations, they can perform similarity analysis of cell types obtained from their mouse models with those from human samples, such as breast cancer.

2. The Jaccard indices did not support the discrimination of M1/2 either.

3. The visualization approach, which utilizes normalized expression in a heatmap, fails to address inadequate discrimination among cell clusters effectively. The normalization process unavoidably amplifies the mean expression of clusters, regardless of the presence or absence of significant disparities.

Reviewer #2 (Remarks to the Author):

Li et al present a revised manuscript. Unfortunately significant flaws exist and make the story uninterpretable and introduce confusion to the field. The major problem, as discussed by several reviewers in the first review is the dependence on using M1/M2; adding "-like" does not help.

Beyond the over simplification they use Supl. Fig. 1F to support their phenotype calling of either "M1" or "M2". But their canonical classification of M1 and M2 markers in Supl Fig 1F is flawed. There is a mix of markers that would indicate pro-tumor and anti-tumor function

within the so called “M1”. Also several markers are misclassified; ie Cd36 and others. CSF1R and C5aR1 are missing from this heatmap. The “M1” express just as many markers of suppressive function as they express markers of anti-tumor immunity further highlighting the need to more carefully dissect the diversity. The authors have single cell data and this should be required.

Comments like: “However, consistent with the plasticity of TAM as well as differences in in vitro and in vivo tumor models [39, 42], the M1-like TAM do express high levels of some canonical M2 markers and similarly the M2-like TAM does express high levels of some canonical M1 markers” cause confusion in the field and do not move the field forward. Authors must describe the subsets of macrophages with marker expression using unbiased clustering analysis.

Another speculative statement that includes oversimplification and confusion is: “Our data and that of others suggests that targeting CSF1R would block both M1-like and M2- like macrophages and indeed potentially selectively inhibit M1-like anti-tumor macrophages that could contribute to the limited efficacy of CSF1R inhibitors in the clinic”. The authors already commented that CSF1R and C5aR1 are expressed by different subsets of macrophages. These subsets need to be defined using modern analysis tools.

Overall, the team’s dependence on calling macrophages M1/M2 to characterize macrophage phenotype is not timely and delays the field. The M1/M2 ratios and analysis are meaningless given these populations do not exist in nature. The group has single cell data and it would be very simple to classify the cells using precise marker definitions as all of the most recent papers have.

I recommend rejecting this paper unless the group would revise ALL of their language to remove all reference of M1 and M2 and describe the cell population more appropriately, which can absolutely be done. For example, they would simply convert this sentence:

C5ar1 and Rps19/C5ar1 signaling are selectively elevated in the M2-like macrophages that are

associated with PARPi resistance. Indeed, C5aR1 positive cells were sufficient to transfer resistance to PARPi. Strikingly targeting C5aR1 decreased M2-like macrophage numbers, while
sparing M1-like macrophages rendering PARPi-resistant tumors sensitive to PARPi in a CD8 T cell dependent manner.

TO:

C5ar1 and Rps19/C5ar1 signaling are selectively elevated in the macrophages that are associated with PARPi resistance. Indeed, C5aR1 positive cells were sufficient to transfer resistance to PARPi. Strikingly targeting C5aR1 macrophages, while
sparing [define macrophages that are spared based on genes they express] macrophages associated with [functional gene signatures] rendering PARPi-resistant tumors sensitive to PARPi in a CD8 T cell dependent manner.

Reviewer #3 (Remarks to the Author):

The authors have addressed all my comments satisfactorily.

Reviewer #4 (Remarks to the Author):

The authors have not addressed the concerns raised by us and other reviewers. There are major problems with this study that render it unsuitable for publication in Nature Communications. These fundamental concerns about the validity of the main conclusions drawn from the work are accompanied by many technical shortcomings in terms of statistical testing, experimental design, and data presentation.

Major concerns

1. The data provided to validate the HR status of cells is overall insufficient and contrary to the author's conclusion. We concur with Reviewer 2 that the model system presented here does not reflect an unmet clinical need since PARPi are only approved for HR deficient

cancer. This is because synthetic lethality upon PARPi occurs in HR deficient cells, and this kills tumors. It is true that PARPi resistance commonly emerges, and this is an unmet clinical need.

2. We also strongly concur with the view of the other reviewers on M1/M2 as presented in this paper. It is clear from the analysis presented here as well as many other emerging studies that the artificial grouping of tumor-associated macrophages into M1/M2 is not reflected in vivo. The author's main conclusions are based on this grouping without further stratification and/or functional insight into macrophage populations. Indeed, their own data shows that there are several other groups of macrophages in tumors derived from their mouse models. In patients, CD163 in tumors does not predict survival in their analysis. It is still completely unclear whether C5aR1 is present in macrophages that contribute to PARPi sensitivity.

Reviewer #1 (Remarks to the Author):

In general, the manuscript has been improved. The authors have addressed most of my previous comments. However, as I raised previously, the fundamental concerns regarding single-cell analysis persist that need to be addressed.

1. Regarding the clustering and annotation, the authors used scPred to obtain cell subsets from the training of single-cell data from the human. However, several studies have reported that non-conserved immune cell subsets exist between human and mouse models, especially the macrophages. The authors should perform clustering on their data independently. For the relevance of human populations, they can perform similarity analysis of cell types obtained from their mouse models with those from human samples, such as breast cancer.

As suggested, we re-did the macrophage analysis by unsupervised clustering of our single cell data. We then re-clustered the myeloid cells identified in the unsupervised clustering to develop leiden clusters of the myeloid population. For monocyte and macrophage annotation, we combined the canonical monocyte markers like Cd14, Fcgr3 (new Figure 1E) with the cluster specific monocyte related genes like Cebpb, S100a6 (new Figure S1G) and identified two different monocyte populations designated Mono_C2 and Mono_C5. We used canonical macrophage markers including Adgre1, Itgam and Cd68 (new Figure 1E) combined with the cluster specific macrophage related genes including Hexba, Il1b, Mrc1 and Lsp1 (new Figure S1G) to identify TAM_C0, TAM_C1, TAM_C3 and TAM_C4. To link the clusters to myeloid functions, we first referred to Wang et al.'s recent work about gastric adenocarcinoma immune and stromal cell evolution for macrophage functional pathway gene lists with a minor modification to link the data to the breast cancer subsets in this study[1]. As previously indicated by multiple manuscripts[2-4], the TAM clusters express a mixture of both anti-inflammatory and pro-inflammatory genes (new Figure S1H and I). We then used label transfer and a human pan-cancer TiME single cell RNA-seq dataset as a reference to further decipher the composition of macrophage in our TAM clusters which also demonstrate that the TAM clusters contain a mixture of cells that expressed markers consistent with both pro- and anti-inflammatory macrophage characteristics (new Figure S1J). However, remarkably, TAM_C1, TAM_C4 and TAM_C0 are dominated by pro-inflammatory phenotypes and in contrast, TAM_C3 is dominated by markers consistent with anti-inflammatory and pro-tumorigenic functions.

Based on Cell Chat and the ability to transfer resistance to olaparib, a C5aR1 macrophage population appeared to represent the critical mediator of the ability of T127 to render T22 resistant to PARP inhibition. This population was primarily composed of cells from TAM_C3 and importantly expressed almost exclusively markers associated with anti-inflammatory and pro-tumorigenic functions.

2. The Jaccard indices did not support the discrimination of M1/2 either.

As indicated above, we have removed the previous scPred annotation method and the Jaccard indices associated with this approach. We subsequently are-annotated the myeloid clusters with unsupervised leiden clustering and then depicted their phenotype by functional pathway and label transfer approaches. New data was shown in new Figure S1H-J. As noted above, the different clusters express markers of both pro-inflammatory and anti-tumorigenic characteristics as seen in previous publications. However, as noted above, these clusters were dominated by cells with pro-inflammatory or pro-tumorigenic characteristics. This was most clearly manifest in the C5aR1 positive macrophage subset that was associated almost exclusively with markers of pro-tumorigenic function.

3. The visualization approach, which utilizes normalized expression in a heatmap, fails to address inadequate discrimination among cell clusters effectively. The normalization process unavoidably amplifies the mean expression of clusters, regardless of the presence or absence of significant disparities.

As suggested, we have removed the heatmap as the primary visualization approach. We have used violin plots and dot plots to provide a better visualization of differences in gene expression and relative expression levels.

Reviewer #2 (Remarks to the Author):

Li et al present a revised manuscript. Unfortunately significant flaws exist and make the story uninterpretable and

introduce confusion to the field. The major problem, as discussed by several reviewers in the first review is the dependence on using M1/M2; adding "-like" does not help.

As suggested, we have removed the terms M1/M2 from the manuscript. We have revised the clustering approach as described in detail in the response to reviewer #1. Briefly, we used an unsupervised leiden clustering approach the myeloid population in our data set and then used the top DEGs for their annotations. Potential functions of each of the subsequent clusters was depicted based on previously reported gene lists. Label transfer was then used to relate the clusters identified on our data with from previously reported macrophage subtypes[1,3]. This analysis is shown in new Figure S1H-J.

Beyond the over simplification they use Supl. Fig. 1F to support their phenotype calling of either "M1" or "M2". But their canonical classification of M1 and M2 markers in Supl Fig 1F is flawed. There is a mix of markers that would indicate pro-tumor and anti-tumor function within the so called "M1". Also several markers are misclassified; ie Cd36 and others. CSF1R and C5aR1 are missing from this heatmap. The "M1" express just as many markers of suppressive function as they express markers of anti-tumor immunity further highlighting the need to more carefully dissect the diversity. The authors have single cell data and this should be required.

As suggested, we have revised the gene lists that are used to identify characteristics of the macrophage populations in each of the clusters identified in our data. We have used gene lists related to macrophage function as anti- or pro-tumor with minor modifications to related to the breast cancer models in this paper based on a recently published paper in Cancer Cell[1]. As suggested in a prior review, we have completely removed comparisons between CSF1R and C5aR1 macrophages. C5aR1 is added to both Figure 1E and Figure S1H. As described in detail above we clustered the complete data set and then re-clustered the myeloid cells based on unsupervised leiden clusters as suggested another reviewer. Similar to studies by others noted above, the subsequent TAM clusters exhibited a mixture of pro- and anti- inflammatory and anti-tumor phenotypes. However, TAM_C1, TAM_C4 and TAM_C0 were dominated by markers associated with pro-inflammatory functions, while in contrast, TAM_C3 was dominated by markers associated with an anti-inflammatory and anti-tumor function. Based on CellChat and ability to transfer PARP resistance to T22 implicating C5ar1 positive macrophages, we found that the C5ar1_hi macrophage population was associated with an almost exclusive anti-inflammatory function. Indeed, C5aR1epression was able to subset TAM_C3 into populations with dominant markers of anti-inflammatory pro-inflammatory functions. This new analysis is presented in Figure S1H-J, Figure 2F-G and Figure S3F in the current manuscript.

Comments like: "However, consistent with the plasticity of TAM as well as differences in in vitro and in vivo tumor models [39, 42], the M1-like TAM do express high levels of some canonical M2 markers and similarly the M2-like TAM does express high levels of some canonical M1 markers" cause confusion in the field and do not move the field forward. Authors must describe the subsets of macrophages with marker expression using unbiased clustering analysis.

As requested, we have described the subsets of macrophages with marker expression derived from unbiased clustering analysis.

Another speculative statement that includes oversimplification and confusion is: "Our data and that of others suggests that targeting CSF1R would block both M1-like and M2- like macrophages and indeed potentially selectively inhibit M1-like anti-tumor macrophages that could contribute to the limited efficacy of CSF1R inhibitors in the clinic". The authors already commented that CSF1R and C5aR1 are expressed by different subsets of macrophages. These subsets need to be defined using modern analysis tools.

As suggested, have removed comparisons between CSF1R and C5aR1 macrophage populations in the new revision. We have instead focused on the C5aR1 positive population and its potential role in mediating resistance to PARPi and the potential to target this population of macrophages.

Overall, the team's dependence on calling macrophages M1/M2 to characterize macrophage phenotype is not timely and delays the field. The M1/M2 ratios and analysis are meaningless given these populations do not exist in nature. The group has single cell data and it would be very simple to classify the cells using precise marker definitions as all of the most recent papers have.

As detailed above in response to reviewer 1, we have repeated the analysis as requested. This is described again here.
As suggested, we re-did the macrophage analysis by unsupervised clustering of our single cell data. We then re-clustered the myeloid cells identified in the unsupervised clustering to develop leiden clusters of the myeloid population. For monocyte and macrophage annotation, we combined the canonical monocyte markers like Cd14, Fcgr3 (new Figure 1E) with the cluster specific monocyte related genes like Cebpb, S100a6 (new Figure S1G) and identified two different monocyte populations designated Mono_C2 and Mono_C5. We used canonical macrophage markers including Adgre1, Itgam and Cd68 (new Figure 1E) combined with the cluster specific macrophage related genes including Hexba, Il1b, Mrc1 and Lsp1 (new Figure S1G) to identify TAM_C0, TAM_C1, TAM_C3 and TAM_C4. To link the clusters to myeloid functions, we first referred to Wang et al.'s recent work about gastric adenocarcinoma immune and stromal cell evolution for macrophage functional pathway gene lists with a minor modification to link the data to the breast cancer subsets in this study[1]. As previously indicated by multiple manuscripts [2-4], the TAM clusters express a mixture of both anti-inflammatory and pro-inflammatory genes (new Figure S1H and I). We then used label transfer and a human pan-cancer TiME single cell RNA-seq dataset as a reference to further decipher the composition of macrophage in our TAM clusters which also demonstrate that the TAM clusters contain a mixture of cells that expressed markers consistent with both pro- and anti-inflammatory macrophage characteristics (new Figure S1J). However, remarkably, TAM_C1, TAM_C4 and TAM_C0 are dominated by pro-inflammatory phenotypes and in contrast, TAM_C3 is dominated by markers consistent with anti-inflammatory and pro-tumorigenic functions.

Based on Cell Chat and the ability to transfer resistance to olaparib, a C5aR1 macrophage population appeared to represent the critical mediator of the ability of T127 to render T22 resistant to PARP inhibition. This was supported further by the ability of inhibition of C5aR1 to reverse resistance to olaparib in both the intrinsically resistant T127 model as well as T22 in the co-transplantation model. The C5aR1 population was primarily composed of cells from TAM_C3 and importantly expressed almost exclusively markers associated with anti-inflammatory and pro-tumorigenic functions.

I recommend rejecting this paper unless the group would revise ALL of their language to remove all reference of M1 and M2 and describe the cell population more appropriately, which can absolutely be done. For example, they would simply convert this sentence:

C5ar1 and Rps19/C5ar1 signaling are selectively elevated in the M2-like macrophages that are associated with PARPi resistance. Indeed, C5aR1 positive cells were sufficient to transfer resistance to PARPi. Strikingly targeting C5aR1 decreased M2-like macrophage numbers, while sparing M1-like macrophages rendering PARPi-resistant tumors sensitive to PARPi in a CD8 T cell dependent manner.

TO:

C5ar1 and Rps19/C5ar1 signaling are selectively elevated in the macrophages that are associated with PARPi resistance. Indeed, C5aR1 positive cells were sufficient to transfer resistance to PARPi. Strikingly targeting C5aR1 macrophages, while sparing [define macrophages that are spared based on genes they express] macrophages associated with [functional gene signatures] rendering PARPi-resistant tumors sensitive to PARPi in a CD8 T cell dependent manner.

As suggested, we have completely revised the manuscript and have removed all use of the terms M1 and M2. We have re-clustered the macrophages in an unbiased manner and have characterized each of the subsets using a marker set from a recent Cell publication. We have carefully defined the markers used to describe each of the subsets and their potential functional characteristics in the paper.

Reviewer #3 (Remarks to the Author):

The authors have addressed all my comments satisfactorily.

Thank you

Reviewer #4 (Remarks to the Author):

The authors have not addressed the concerns raised by us and other reviewers. There are major problems with this study that render it unsuitable for publication in Nature Communications. These fundamental concerns about the validity of the main conclusions drawn from the work are accompanied by many technical shortcomings in terms of statistical testing, experimental design, and data presentation.

Major concerns

1. The data provided to validate the HR status of cells is overall insufficient and contrary to the author's conclusion. We concur with Reviewer 2 that the model system presented here does not reflect an unmet clinical need since PARPi are only approved for HR deficient cancer. This is because synthetic lethality upon PARPi occurs in HR deficient cells, and this kills tumors. It is true that PARPi resistance commonly emerges, and this is an unmet clinical need.

As described in the manuscript and referenced in detail, we agree that PARP inhibitor monotherapy is active primarily in tumors with HR deficiency. However, multiple clinical trials referenced in the manuscript have demonstrated that PARP inhibitor combination therapy is highly active in HR competent tumors [5-13] (see references 9,11-19 in the manuscript and the reviewer is referred to multiple clinical trials that have demonstrated activity of PARP inhibitor combinations in HR competent tumors: NCT03162627, NCT04197713, NCT02208375, NCT03579316 NCT03801369 amongst others). Further as indicated by the reviewer PARP inhibitor resistance remains a problem. Even in patients with BRCA1/2 mutations and HR deficiency only a sub population of patients demonstrate significant responses to PARP inhibitor monotherapy. Further, a significant number of patients who respond to PARP inhibitors demonstrate acquired resistance. Thus, as indicated by the reviewer *"It is true that PARPi resistance commonly emerges, and this is an unmet clinical need."* The studies in this manuscript provide a potential approach to prevent or reverse PARPi resistance.

LPA1-T22 and LPA1-T27 have been extensively characterized previously. Both are homologous recombination competent based on not demonstrating mutations of 33 genes involved in homologous recombination and by the ability to form RAD51 foci in response to DNA damaging agents including irradiation (IR)[14-16]. This is now included in the revised manuscript.

2. We also strongly concur with the view of the other reviewers on M1/M2 as presented in this paper. It is clear from the analysis presented here as well as many other emerging studies that the artificial grouping of tumor-associated macrophages into M1/M2 is not reflected in vivo. The author's main conclusions are based on this grouping without further stratification and/or functional insight into macrophage populations. Indeed, their own data shows that there are several other groups of macrophages in tumors derived from their mouse models. In patients, CD163 in tumors does not predict survival in their analysis. It is still completely unclear whether C5aR1 is present in macrophages that contribute to PARPi sensitivity.

As suggested, we re-did the macrophage analysis by unsupervised clustering of our single cell data. We then re-clustered the myeloid cells identified in the unsupervised clustering to develop leiden clusters of the myeloid population. For monocyte and macrophage annotation, we combined the canonical monocyte markers like Cd14, Fcgr3 (new Figure 1E) with the cluster specific monocyte related genes like Cebpb, S100a6 (new Figure S1G) and identified two different monocyte populations designated Mono_C2 and Mono_C5. We used canonical macrophage markers including Adgre1, Itgam and Cd68 (new Figure 1E) combined with the cluster specific macrophage related genes including Hexb, Il1b, Mrc1 and Lsp1 (new Figure S1G) to identify TAM_C0, TAM_C1, TAM_C3 and TAM_C4. To link the clusters to myeloid functions, we first referred to Wang et al.'s recent work about gastric adenocarcinoma immune and stromal cell evolution for macrophage functional pathway gene lists with a minor modification to link the data to the breast cancer subsets in this study[1]. As previously indicated by multiple manuscripts[2-4], the TAM clusters express a mixture of both anti-inflammatory and pro-inflammatory genes (new Figure S1H and I). We then used label transfer and a human pan-cancer TiME single cell RNA-seq dataset as a reference to further decipher the composition of macrophage in our TAM clusters which also demonstrate that the TAM clusters contain a mixture of cells that expressed markers consistent with both pro- and anti-inflammatory macrophage characteristics (new Figure S1J). However, remarkably, TAM_C1, TAM_C4 and TAM_C0 are

dominated by pro-inflammatory phenotypes and in contrast, TAM_C3 is dominated by markers consistent with anti-inflammatory and pro-tumorigenic functions.

Based on Cell Chat and the ability to transfer resistance to olaparib, a C5aR1 macrophage population appeared to represent the critical mediator of the ability of T127 to render T22 resistant to PARP inhibition. The ability of a sorted C5aR1 cell population to transfer resistance indicates that this population of cells is sufficient to transfer resistance. Further, we demonstrated that inhibition of C5ar1 was sufficient to reverse resistance to olaparib in both the intrinsically resistant T127 model as well as T22 in the co-transplantation model. Thus, a C5aR1 population appears sufficient and potentially necessary to mediate PARP inhibitor resistance in this model system. The C5ar1 population was primarily composed of cells from TAM_C3 and importantly expressed almost exclusively markers associated with anti-inflammatory and pro-tumorigenic functions providing additional support for a role for C5aR1 positive macrophages in mediating resistance to PARPi. However, we do agree with the reviewer that there are likely additional mechanisms leading to resistance to PARPi as has been described extensively by us and others. Nevertheless, C5aR1 represents an additional target that could potentially prevent acquisition of PARP inhibitor resistance or reverse resistance once it occurs. This will require testing in human clinical trials.

1 Wang, R., Song, S., Qin, J., Yoshimura, K., Peng, F., Chu, Y. *et al.* Evolution of immune and stromal cell states and ecotypes during gastric adenocarcinoma progression. *Cancer Cell* **41**, 1407-1426 e1409, doi:10.1016/j.ccell.2023.06.005 (2023).

2 Bassez, A., Vos, H., Van Dyck, L., Floris, G., Arijs, I., Desmedt, C. *et al.* A single-cell map of intratumoral changes during anti-PD1 treatment of patients with breast cancer. *Nat Med* **27**, 820-832, doi:10.1038/s41591-021-01323-8 (2021).

3 Qian, J., Olbrecht, S., Boeckx, B., Vos, H., Laoui, D., Etlioglu, E. *et al.* A pan-cancer blueprint of the heterogeneous tumor microenvironment revealed by single-cell profiling. *Cell Res* **30**, 745-762, doi:10.1038/s41422-020-0355-0 (2020).

4 Zhang, Y., Chen, H., Mo, H., Hu, X., Gao, R., Zhao, Y. *et al.* Single-cell analyses reveal key immune cell subsets associated with response to PD-L1 blockade in triple-negative breast cancer. *Cancer Cell* **39**, 1578-1593 e1578, doi:10.1016/j.ccell.2021.09.010 (2021).

5 Harter, P., Trillsch, F., Okamoto, A., Reuss, A., Kim, J.-W., Rubio-Pérez, M. J. *et al.* Durvalumab with paclitaxel/carboplatin (PC) and bevacizumab (bev), followed by maintenance durvalumab, bev, and olaparib in patients (pts) with newly diagnosed advanced ovarian cancer (AOC) without a tumor BRCA1/2 mutation (non-tBRCAm): Results from the randomized, placebo (pbo)-controlled phase III DUO-O trial. *Journal of Clinical Oncology*, doi:10.1200/JCO.2023.41.17_suppl.LBA5506 (2023).

6 Gonzalez-Martin, A., Pothuri, B., Vergote, I., Graybill, W., Lorusso, D., McCormick, C. C. *et al.* Progression-free survival and safety at 3.5years of follow-up: results from the randomised phase 3 PRIMA/ENGOT-OV26/GOG-3012 trial of niraparib maintenance treatment in patients with newly diagnosed ovarian cancer. *Eur J Cancer* **189**, 112908, doi:10.1016/j.ejca.2023.04.024 (2023).

7 Banerjee, S., Imbimbo, M., Roxburgh, P., Kim, J.-W., Kim, M. H., Plummer, R. *et al.* Phase II study of olaparib plus durvalumab with or without bevacizumab (MEDIOLA): Final analysis of overall survival in patients with non-germline BRCA-mutated platinum-sensitive relapsed ovarian cancer. *Annals of Oncology*, doi:https://doi.org/10.1016/j.annonc.2022.07.657 (2022).

8 Labrie, M., Li, A., Creason, A., Betts, C., Keck, J., Johnson, B. *et al.* Multiomics analysis of serial PARP inhibitor treated metastatic TNBC inform on rational combination therapies. *NPJ Precis Oncol* **5**, 92, doi:10.1038/s41698-021-00232-w (2021).
9 Chen, Z., Wang, X., Li, X., Zhou, Y. & Chen, K. Deep exploration of PARP inhibitors in breast cancer: monotherapy and combination therapy. *J Int Med Res* **49**, 300060521991019, doi:10.1177/0300060521991019 (2021).
10 Ledermann, J., Harter, P., Gourley, C., Friedlander, M., Vergote, I., Rustin, G. *et al.* Olaparib maintenance therapy in patients with platinum-sensitive relapsed serous ovarian cancer: a preplanned retrospective analysis of outcomes by BRCA status in a randomised phase 2 trial. *Lancet Oncol* **15**, 852-861, doi:10.1016/S1470-2045(14)70228-1 (2014).
11 Poveda, A., Lheureux, S., Colombo, N., Cibula, D., Lindemann, K., Weberpals, J. *et al.* Olaparib maintenance monotherapy in platinum-sensitive relapsed ovarian cancer patients without a germline BRCA1/BRCA2 mutation: OPINION primary analysis. *Gynecol Oncol* **164**, 498-504, doi:10.1016/j.ygyno.2021.12.025 (2022).
12 Lampert, E. J., Zimmer, A., Padget, M., Cimino-Mathews, A., Nair, J. R., Liu, Y. *et al.* Combination of PARP Inhibitor Olaparib, and PD-L1 Inhibitor Durvalumab, in Recurrent Ovarian Cancer: a Proof-of-Concept Phase II Study. *Clin Cancer Res* **26**, 4268-4279, doi:10.1158/1078-0432.CCR-20-0056 (2020).
13 Konstantinopoulos, P. A., Waggoner, S., Vidal, G. A., Mita, M., Moroney, J. W., Holloway, R. *et al.* Single-Arm Phases 1 and 2 Trial of Niraparib in Combination With Pembrolizumab in Patients With Recurrent Platinum-Resistant Ovarian Carcinoma. *JAMA Oncol* **5**, 1141-1149, doi:10.1001/jamaoncol.2019.1048 (2019).
14 Wang, Z., Sun, K., Xiao, Y., Feng, B., Mikule, K., Ma, X. *et al.* Niraparib activates interferon signaling and potentiates anti-PD-1 antibody efficacy in tumor models. *Sci Rep* **9**, 1853, doi:10.1038/s41598-019-38534-6 (2019).
15 Sun, C., Fang, Y., Yin, J., Chen, J., Ju, Z., Zhang, D. *et al.* Rational combination therapy with PARP and MEK inhibitors capitalizes on therapeutic liabilities in RAS mutant cancers. *Sci Transl Med* **9**, doi:10.1126/scitranslmed.aal5148 (2017).
16 Federico, L., Chong, Z., Zhang, D., McGrail, D. J., Zhao, W., Jeong, K. J. *et al.* A murine preclinical syngeneic transplantation model for breast cancer precision medicine. *Sci Adv* **3**, e1600957, doi:10.1126/sciadv.1600957 (2017).

REVIEWER COMMENTS

Reviewer #1 (Remarks to the Author):

Generally, the revised manuscript has been improved. The authors have performed unbiased clustering of their single-cell dataset, as I and other reviewers required. Specifically, they improved the characterization of monocyte/macrophage populations and linked these cell subsets with those from human samples. They also highlighted the relatively high expression of C5aR1 in the TAM_C3 cell subset, which showed a high fraction of tumors resistant to PARP inhibition.

Here are minor points for further improving the readability of figures:

1. Densely packed labels should be adjusted, e.g., Fig. 1E, Fig. S1H, S1G, Fig. 2F, etc.
2. The resolution of some figures is too low, such as Fig.7 and gene labels in supplementary figures.
3. A suspiciously red boxed cross seems to be misplaced in Fig. 1D.
4. Some abbreviations, such as “T127_co” and “T22_co,” could be in full length to improve readability.
5. Please check the consistency of the phrases. For instance, “C5aR1_hi”, “C5aR1+” and “C5aR1hi”.

Reviewer #2 (Remarks to the Author):

Authors have refined their myeloid clusters but still rely on a dichotomy of macrophage phenotype (now using pro- and anti-tumor/ proinflammatory and anti-inflammaotry), which is no better than M1/M2. This dichotomy does not exist in nature. When looking at the genes expressed by each cluster there are features that could resemble both pro or anti-tumor functions. Defining the clusters by function is the most appropriate way to call macrophage subsets which might suggest a phenotype that can drive a pro or anti-tumor phenotype. Since no mechanistic studies were performed to understand each cluster it is only an association with known gene functions. Their continued reliance on the dichotomy of splitting TAMs suggests ignorance in their understanding of myeloid biology.

For example, this whole paragraph is incorrect and there is no reason that it needs to be

included. Instead they need to state that TAMs have a complex phenotype and multiple subsets exist in which they can contain features of both tumor promoting and antitumor phenotypes.

For ease of presentation, we will use the terminology pro- inflammatory and pro-tumor to refer to TAMs that have characteristics associated with antitumor and pro-tumor macrophages, respectively. We realize that this split is simplistic and that there are clearly additional important macrophage subpopulations and further that plasticity can result in challenges in defining the functional characteristics of specific macrophage subpopulations. Thus to improve clarity, the process used to define the pro-inflammatory and pro-tumor subsets of particular TAM populations will be indicated throughout the text. TAMs, particularly pro-inflammatory macrophages, can also serve as a bridge between innate and adaptive immunity through antigen presentation associated with elevated MHC-II gene expression compared to other TAM subtypes [24]. Pro-inflammatory and pro-tumor TAMs mediate many of their pro and anti-tumor activities through production of mutually exclusive sets of cytokines. Pro-inflammatory TAMs express TH1 response-inducing cytokines such as IL1a, IL1ß, IL12 and TNFa while pro-tumor TAMs express IL10, CCL8 and CCL22 that are tumor promoting [25].

Another example is: “Through single cell analysis, we demonstrated that the PARPi (olaparib)-sensitive T22 has substantial pro-inflammatory macrophage content resulting in anti-tumor immune activity and that PARPi increase this population.” As already provided in a previous critique gene that suggest their function need to be included here. The reader has no idea what they mean by pro/anti since there is not definition to these terms.

The way that the authors have written the abstract and other parts of the manuscript seem to contradict each other.

For example, in the abstract they write:

Our data indicate that the PARPi-sensitive tumor model compared to PARPi-resistant tumor model has a higher fraction of macrophages dominated with pro-tumor manner with the fraction of these macrophages being increased by PARPi. In contrast, the PARPi-resistant tumor model has low levels of macrophages dominated with pro-inflammatory manner that are not altered by PARPi. Transplantation of the PARPi-sensitive and the PARPi-resistant

tumor in opposite mammary fat pads, mimicking cross talk between metastatic sites, results in accumulation of pro-tumor macrophages in the sensitive tumor (NOTE: previous text indicates this occurs...so are they sensitive or resistant?), rendering the sensitive tumor PARPi resistant suggesting that transit of pro-tumor macrophages could contribute to resistance across distant sites both within and between tumors.

But in the introduction they write:

Through single cell analysis, we demonstrated that the PARPi (olaparib)-sensitive T22 has substantial pro-inflammatory macrophage content resulting in anti-tumor immune activity and that PARPi increase this population. In contrast, the PARPi resistant tumor model has very low levels of macrophages dominated with pro-inflammatory manner along with a high level of TAM dominated with pro-tumor manner that were not altered by PARPi. By co-transplanting T22 and T127 on opposite mammary fat pads in the same mouse to mimic the effects of different metastatic sites, we observed that the PARPi resistant T127 abolished T22 olaparib sensitivity by remodeling the TAM phenotype in T22 from a pro-inflammatory dominated to a pro-tumor dominated phenotype. Furthermore, C5aR1 macrophages were sufficient to transfer PARPi resistance to the T22 model.

Other concerns:

- I am not able to find DEG files of genes for each macrophage cluster from GSE215908. So I am not able to verify how they are calling macrophages. Based on what they show (a few genes/cluster) would not be sufficient to identify subsets.
- I still have some skepticisms about the use of the T22 and T127 tumors as PARPi sensitive and resistant given they are BRCA and HRD WT.
- Supl. figures are not interpretable because axis are blury.
- The title to Fig S6 needs to be edited. I don’t see data with anti-CSF-1R.

Reviewer #1 (Remarks to the Author):

Generally, the revised manuscript has been improved. The authors have performed unbiased clustering of their single-cell dataset, as I and other reviewers required. Specifically, they improved the characterization of monocyte/macrophage populations and linked these cell subsets with those from human samples. They also highlighted the relatively high expression of C5aR1 in the TAM_C3 cell subset, which showed a high fraction of tumors resistant to PARP inhibition.

Here are minor points for further improving the readability of figures:
1. Densely packed labels should be adjusted, e.g., Fig. 1E, Fig. S1H, S1G, Fig. 2F, etc.
We thank the reviewer for acknowledging the approach and conclusions. We have carefully reviewed the figures and have clarified the figure labels as required.
2. The resolution of some figures is too low, such as Fig.7 and gene labels in supplementary figures.
We thank the reviewer for acknowledging the approach and conclusions. We have carefully reviewed the figures and have upgraded the figure resolutions as required.
3. A suspiciously red boxed cross seems to be misplaced in Fig. 1D.
We thank the reviewer for acknowledging the approach and conclusions. We have carefully reviewed the figures and have removed the boxed cross as required.
4. Some abbreviations, such as “T127_co” and “T22_co,” could be in full length to improve readability.
We thank the reviewer for acknowledging the approach and conclusions. We have carefully considered the comments and have clarified figures the text as required. However, considering T127_co and T22_co appear also frequently in the figures, using full length might make the figure look squeezing, therefore, we decided to stay with the short version in the figures.
5. Please check the consistency of the phrases. For instance, “C5aR1_hi”, “C5aR1+” and “C5aR1hi”.
We thank the reviewer for acknowledging the approach and conclusions. We have carefully reviewed the comments and have clarified figures the text as required.

Reviewer #2 (Remarks to the Author):

Authors have refined their myeloid clusters but still rely on a dichotomy of macrophage phenotype (now using pro- and anti-tumor/ proinflammatory and anti-inflammaotry), which is no better than M1/M2. Defining the clusters by function is the most appropriate way to call macrophage subsets which might suggest a phenotype that can drive a pro or anti-tumor phenotype. Since no mechanistic studies were performed to understand each cluster it is only an association with known gene functions. Their continued reliance on the dichotomy of splitting TAMs suggests ignorance in their understanding of myeloid biology.
For example, this whole paragraph is incorrect and there is no reason that it needs to be included. Instead they need to state that TAMs have a complex phenotype and multiple subsets exist in which they can contain features of both tumor promoting and antitumor phenotypes.

For ease of presentation, we will use the terminology pro- inflammatory and pro-tumor to refer to TAMs that have characteristics associated with antitumor and pro-tumor macrophages, respectively. We realize that this split is simplistic and that there are clearly additional important macrophage subpopulations and further that plasticity can result in challenges in defining the functional characteristics of specific macrophage subpopulations. Thus to improve clarity, the process used to define the pro-inflammatory and pro-tumor subsets of particular TAM populations will be indicated throughout the text. TAMs, particularly pro-inflammatory macrophages, can also serve as a bridge between innate and adaptive immunity through antigen presentation associated with elevated MHC-II gene expression compared to other TAM subtypes [24]. Pro-inflammatory and pro-tumor TAMs mediate many of their pro and anti-tumor activities through production of mutually exclusive sets of cytokines. Pro-inflammatory TAMs express TH1 response-inducing cytokines such as IL1a, IL1ß, IL12 and TNFa while pro-tumor TAMs express IL10, CCL8 and CCL22 that are tumor promoting [25]. Another example is: "Through single cell analysis, we demonstrated that the PARPi (olaparib)-sensitive T22 has substantial pro-inflammatory macrophage content resulting in anti-tumor immune activity and that PARPi increase this population." As already provided in a previous critique gene that suggest their function need to be included here. The reader has no idea what they mean by pro/anti since there is not definition to these terms.

As suggested we have revised these statements as suggested.

We understand the concern with dichotomizing macrophage populations. This is why we have clarified in the text what information is being used in the description of macrophages and their potential functions. We now define macrophages by their leiden cluster ID and indicate where they express genes that could relate to potential functions and use GSVA analysis with KEGG, reactome and GO genesets to reveal potential biological functions (new Figure S2A). We have also explored functional impact by exploring association with outcomes in the TCGA basal breast cancer datasets (new Figure S2B). This analysis provided further support for TAM_C3 having relatively higher lipid metabolism activity and the association of TAM_C3 with an unfavorable outcome in basal breast cancer. Based on this information as well as the dominant expression of RNAs associated with an anti-inflammatory function, TAM_C3 appears to contribute to PARP resistance and poor outcome. These results suggest that TAM_C3 represents a relevant macrophage subtype with this information helping to understand the role of macrophages and particular TAM_C3 in response to PARP inhibitors.

Genes associated with pro- and anti-inflammatory function as defined in a major recent publication (PMID: 37419119) are presented in Figure S1H as well as their association with different TAM subtypes defined in this manuscript. A summary of scores of the different functions and how they relate to the different TAM subtypes defined in this manuscript are present in Figure S1L.

The way that the authors have written the abstract and other parts of the manuscript seem to contradict each other.

For example, in the abstract they write:
Our data indicate that the PARPi-sensitive tumor model compared to PARPi-resistant tumor model has a higher fraction of macrophages dominated with pro-tumor manner with the fraction of these macrophages being increased by PARPi. In contrast, the PARPi-resistant tumor model has low levels of macrophages dominated with pro-inflammatory manner that are not altered by PARPi. Transplantation of the PARPi-sensitive and the PARPi-resistant tumor in opposite mammary fat pads, mimicking cross talk between metastatic sites, results in accumulation of pro-tumor macrophages in the sensitive tumor (NOTE: previous text indicates this occurs...so are they sensitive or resistant?), rendering the sensitive tumor PARPi resistant suggesting that transit of pro-tumor macrophages could contribute to resistance across distant sites both within and between tumors.
But in the introduction they write:
Through single cell analysis, we demonstrated that the PARPi (olaparib)-sensitive T22 has substantial pro-inflammatory macrophage content resulting in anti-tumor immune activity and that PARPi increase this population. In contrast, the PARPi resistant tumor model has very low levels of macrophages dominated with pro-inflammatory manner along with a high level of TAM dominated with pro-tumor manner that were not altered by PARPi. By co-transplanting T22 and T127 on opposite mammary fat pads in the same mouse to mimic the effects of different metastatic sites, we observed that the PARPi resistant T127 abolished T22 olaparib sensitivity by remodeling the TAM phenotype in T22 from a pro-inflammatory dominated to a pro-tumor dominated phenotype. Furthermore, C5aR1 macrophages were sufficient to transfer PARPi resistance to the T22 model.

Based on the concerns about clarity, we have modified the abstract and results to increase the ease of understanding. The concern arises from whether the basal state of T22 (intrinsically sensitive) and T127 (intrinsically resistant) is being considered as compared to the state of the tumors when transplanted on opposite sides of the mouse. This co-transplantation results in the intrinsically PARPi sensitive T22 becoming PARPi resistant. It also arises from the description of the relative numbers of different types of macrophages. We have revised the two sections to make the reading more clear. Importantly, we have now used the macrophage subtypes identified in this manuscript as the basis for these two sections.

We have revised the abstract to:
Although Poly (ADP-ribose) polymerase (PARP) inhibitors (PARPi) have been approved in multiple diseases, including BRCA1/2 mutant breast cancer, responses are usually transient requiring the deployment of combination therapies that can prevent or reverse PARPi resistance. We thus explored mechanisms underlying sensitivity and resistance to PARPi using two intrinsically PARPi sensitive (T22) and resistant (T127) syngeneic murine breast cancer models. Our data identified tumor associated macrophages (TAM)

as potentially contributing to the differential PARPi sensitivity in T22 and T127. TAM_C0 (with a mixed expression of pro- and anti-inflammatory genes) and TAM_C1 (with a dominant expression of pro-inflammatory genes) were markedly higher in the intrinsically PARPi-sensitive T22 compared to the PARPi-resistant T127. TAM_C3 that expressed genes implicated in anti-inflammatory activity was lower in T22 than T127 and was markedly decreased by PARPi in T22. C5aR1 and Rps19/C5aR1 signaling are selectively elevated in TAM_C3. TAM_C3 could be subdivided into two subsets C5aR1 high (with a dominant expression of anti-inflammatory/pro-tumor genes) and C5aR1 low (without significant expression of anti-or pro-inflammatory genes). Indeed, C5aR1 high cells were sufficient to transfer PARPi resistance to the intrinsically PARPi-sensitive T22 model. Strikingly targeting C5aR1 rendered PARPi-resistant tumors sensitive to PARPi in a CD8 T cell dependent manner. Consistent with the murine data, high C5aR1 levels in human breast cancers are associated with a poor response to immune checkpoint blockade. Thus, targeting C5aR1 may represent an approach to selectively deplete pro-tumor macrophages and engender sensitivity to PARPi and potentially other therapies.

And the relevant part of the introduction to:
Single cell RNA analysis identified 4 tumor associated macrophage (TAM) subtypes: TAM_C0, TAM_C1, TAM_C3 and TAM_C4 in the T22 and T127 models. TAM_C0 that had a mixed expression of genes associated pro- and anti-inflammatory states (PMID: 37419119) were present at elevated levels in the T22 intrinsically PARPi tumors. TAM_C3 that mainly expressed genes associated with a pro-inflammatory state (PMID: 37419119) was also elevated in T22. TAM_C3 that mainly expressed genes associated with an anti-inflammatory/protumor state was lower in the intrinsically PARPi sensitive T22 than in the PARPi resistant T127 and was markedly decreased by PARPi in T22. To mimic metastatic states, we implanted T22 and T127 in opposite mammary fat pads. This resulted in the intrinsically PARPi sensitive T22 becoming PARPi resistant. C5aR1 positive macrophages that represent TAM_C3 were sufficient to render T22 resistant to PARPi. Strikingly targeting C5aR1 decreased $CD206^{+}$ macrophages in the PARPi-resistant models while sparing $MHCII^{high}$ macrophages leading to both models becoming sensitive to PARPi.

Other concerns:

• I am not able to find DEG files of genes for each macrophage cluster from GSE215908. So I am not able to verify how they are calling macrophages. Based on what they show (a few genes/cluster) would not be sufficient to identify subsets.
As suggested, we added list of DEGs from GSE215908 that were used to define each macrophage cluster to supplementary Table S3.

• I still have some skepticisms about the use of the T22 and T127 tumors as PARPi sensitive and resistant given they are BRCA and HRD WT.
As indicated in multiple references quoted in the manuscript as well as in multiple clinical trials PARPi does demonstrate activity in BRCA and HRDS WT tumors

particularly when used in combinations. T22 is intrinsically sensitive to PARPi and T127 is intrinsically resistant. They are thus relevant models to understand why PARPi are active in a subset of BRCA and HRD WT tumors. While these are not perfect models, they have elucidated an ability of PARPi resistance to be transferred from T127 to T22 tumors that led to an exploration of a C5aR1 positive macrophage subtype as a therapeutically relevant macrophage subtype.

• Supl. figures are not interpretable because axis are blury.
As suggested, the resolution of figures has been approved. We apologize for the "blurry" nature of the supplemental figures that happened during uploading

• The title to Fig S6 needs to be edited. I don't see data with anti-CSF-1R.
As suggested, anti-CSF-1R was deleted and we have now revised the title as requested. Thank you for pointing out this error.

REVIEWERS' COMMENTS

Reviewer #1 (Remarks to the Author):

The authors properly addressed all my remaining concerns and comments. Regarding the classification and annotation of TAM subsets, the authors have leveraged a published study of gastric cancer to generate the scores of different functions of TAMs. However, I did not find Figure S1L as the authors indicated for such a comparative analysis. The authors should check the figures and citations in the manuscript.

Reviewer #2 (Remarks to the Author):

Authors have satisfactorily addressed concerns.

Reviewer #1 (Remarks to the Author):

Reviewer #1 (Remarks to the Author):

The authors properly addressed all my remaining concerns and comments. Regarding the classification and annotation of TAM subsets, the authors have leveraged a published study of gastric cancer to generate the scores of different functions of TAMs. However, I did not find Figure S1L as the authors indicated for such a comparative analysis. The authors should check the figures and citations in the manuscript.

Thank you for pointing this out. We apologize for the typo in our last point-to-point response. In last rebuttal letter, we wrote "*A summary of scores of the different functions and how they relate to the different TAM subtypes defined in this manuscript are present in Figure S1L.*" which is s typo. We have checked the figure indices in the text and the supplementary information data, the one we were referring should be Figure S1I.

Reviewer #2 (Remarks to the Author):

Authors have satisfactorily addressed concerns.

Thank you.
